# Supplementary material for: Coumarinolignans with Reactive Oxygen Species (ROS) and NF-κB Inhibitory Activities from the Roots of Waltheria indica
Source: Molecules. 2022 May 19;27(10):3270. doi: 10.3390/molecules27103270 (PMC9147481; doi:10.3390/molecules27103270)
Supplement: Supplementary file 1 [file molecules-27-03270-s001.zip › molecules-1655366-supplementary.pdf]

Coumarinolignans with Reactive Oxygen Species (ROS) and NF-κB Inhibitory Activities from the Roots of *Waltheria indica*

Feifei Liu [1,2], Sudipta Mallick [1,†], Timothy J. O'Donnell [3,†], Ruxianguli Rouzimaimaiti [1], Yuheng Luo [3], Rui Sun [3], Marisa Wall [4], Supakit Wongwiwatthananukit [5], Abhijit Date [1,6], Dane Kaohelani Silva [7], Philip G. Williams [3] and Leng Chee Chang [1,*]

1 Department of Pharmaceutical Sciences, Daniel K. Inouye College of Pharmacy, University of Hawaiʻi at Hilo, Hilo, HI 96720, USA; feifeiliu@jsnu.edu.cn (F.L.); sudipta1787@gmail.com (S.M.); ruxian.love@163.com (R.R.); dateabhi@hawaii.edu (A.D.)

2 School of Life Sciences, Jiangsu Normal University, Xuzhou 221116, China

3 Department of Chemistry, 2545 McCarthy Mall, University of Hawaiʻi at Manoa, Honolulu, HI 96822, USA; tjod@hawaii.edu (T.J.O.); yuheng@hawaii.edu (Y.L.); ruisun@hawaii.edu (R.S.); philipwi@hawaii.edu (P.G.W.)

4 Daniel K. Inouye U.S. Pacific Basin Agricultural Research Center, USDA-ARS, Hilo, HI 96720, USA; marisa.wall@usda.gov

5 Department of Pharmacy Practice, Daniel K. Inouye College of Pharmacy, University of Hawaiʻi at Hilo, Hilo, HI 96720, USA; supakit@hawaii.edu

6 Department of Pharmacology and Toxicology, R. K. Coit College of Pharmacy, University of Arizona, Tucson, AZ 85715, USA.

7 Hale Ola Pono, LLC, Keaau, HI 96749, USA; lomidoc@gmail.com

* Correspondence: lengchee@hawaii.edu; Tel.: +1-(808)-981-8018

† These authors contributed equally to this work.

Contents

Figure S1. HRESI-MS spectrum of the new compound **1a** 1

Figure S2. IR spectrum of the new compound **1a** 2

Figure S3. ^{1}H NMR (400 MHz, $CDCl_3$) spectrum of the new compound **1a** 3

Figure S4. ^{13}C NMR (100 MHz, $CDCl_3$) spectrum of the new compound **1a** 4

Figure S5. HSQC spectrum of the new compound **1a** 5

Figure S6. HMBC spectrum of the new compound **1a** 6

Figure S7. COSY spectrum of the new compound **1a** 7

Figure S8. NOESY spectrum of the new compound **1a** 8

Figure S9. HRESI-MS spectrum of the new compound **1b** 9

Figure S10. IR spectrum of the new compound **1b** 10

Figure S11. ^{1}H NMR (400 MHz, $CDCl_3$) spectrum of the new compound **1b** 11

Figure S12. ^{13}C NMR (100 MHz, $CDCl_3$) spectrum of the new compound **1b** 12

Figure S13. HSQC spectrum of the new compound **1b** 13

Figure S14. HMBC spectrum of the new compound **1b** 14

Figure S15. COSY spectrum of the new compound **1b** 15

Figure S16. NOESY spectrum of the new compound **1b** 16

Figure S17. HRESI-MS spectrum of the new compound **2** 17

Figure S18. IR spectrum of the new compound **2** 18

Figure S19. ^{1}H NMR (400 MHz, MeOD) spectrum of the new compound **2** 19

Figure S20. ^{13}C NMR (100 MHz, MeOD) spectrum of the new compound **2** 20

Figure S21. HSQC spectrum of the new compound **2** 21

Figure S22. HMBC spectrum of the new compound **2** 22

Figure S23. COSY spectrum of the new compound **2** 23

Figure S24. NOESY spectrum of the new compound **2** 24

Figure S25. Images of conformers (>1%) for the new compound **2** 25

Figure S26. CD spectrum (experimental to calculated comparison by SpecDis) of the new compound **2** 26

Figure S27. HRESI-MS spectrum of the new compound **3** 27

Figure S28. IR spectrum of the new compound **3** 28

Figure S29. ^{1}H NMR (400 MHz, $CDCl_3$) spectrum of the new compound **3** 29

Figure S30. ^{13}C NMR (100 MHz, $CDCl_3$) spectrum of the new compound **3** 30

Figure S31. HSQC spectrum of the new compound **3** 31

Figure S32. HMBC spectrum of the new compound **3** 32

Figure S33. COSY spectrum of the new compound **3** 33

Figure S34. NOESY spectrum of the new compound **3** 34

Figure S35. Images of conformers (>1%) for the new compound **3** 35

Figure S36. CD spectrum (experimental to calculated comparison by SpecDis) of the new compound **3** 36
Figure S37. HRESI-MS spectrum of the new compound **4** 37
Figure S38. IR spectrum of the new compound **4** 38
Figure S39. ^{1}H NMR (400 MHz, MeOD) spectrum of the new compound **4** 39
Figure S40. ^{13}C NMR (100 MHz, MeOD) spectrum of the new compound **4** 40
Figure S41. HSQC spectrum of the new compound **4** 41
Figure S42. HMBC spectrum of the new compound **4** 42
Figure S43. COSY spectrum of the new compound **4** 43
Figure S44. NOESY spectrum of the new compound **4** 44
Figure S45. HRESI-MS spectrum of the new compound **5** 45
Figure S46. IR spectrum of the new compound **5** 46
Figure S47. ^{1}H NMR (400 MHz, $CDCl_3$) spectrum of the new compound **5** 47
Figure S48. ^{13}C NMR (100 MHz, $CDCl_3$) spectrum of the new compound **5** 48
Figure S49. HSQC spectrum of the new compound **5** 49
Figure S50. HMBC spectrum of the new compound **5** 50
Figure S51. COSY spectrum of the new compound **5** 51
Figure S52. NOESY spectrum of the new compound **5** 52
Figure S53. HRESI-MS spectrum of the new compound **7** 53

Figure S54. IR spectrum of the new compound **7** 54

Figure S55. ^{1}H NMR (400 MHz, $CDCl_3$) spectrum of the new compound **7** 55

Figure S56. ^{13}C NMR (100 MHz, $CDCl_3$) spectrum of the new compound **7** 56

Figure S57. HSQC spectrum of the new compound **7** 57

Figure S58. HMBC spectrum of the new compound **7** 58

Figure S59. COSY spectrum of the new compound **7** 59

Figure S60. NOESY spectrum of the new compound **7** 60

Figure S61. Images of conformers (>1%) for the new compound **7** 61

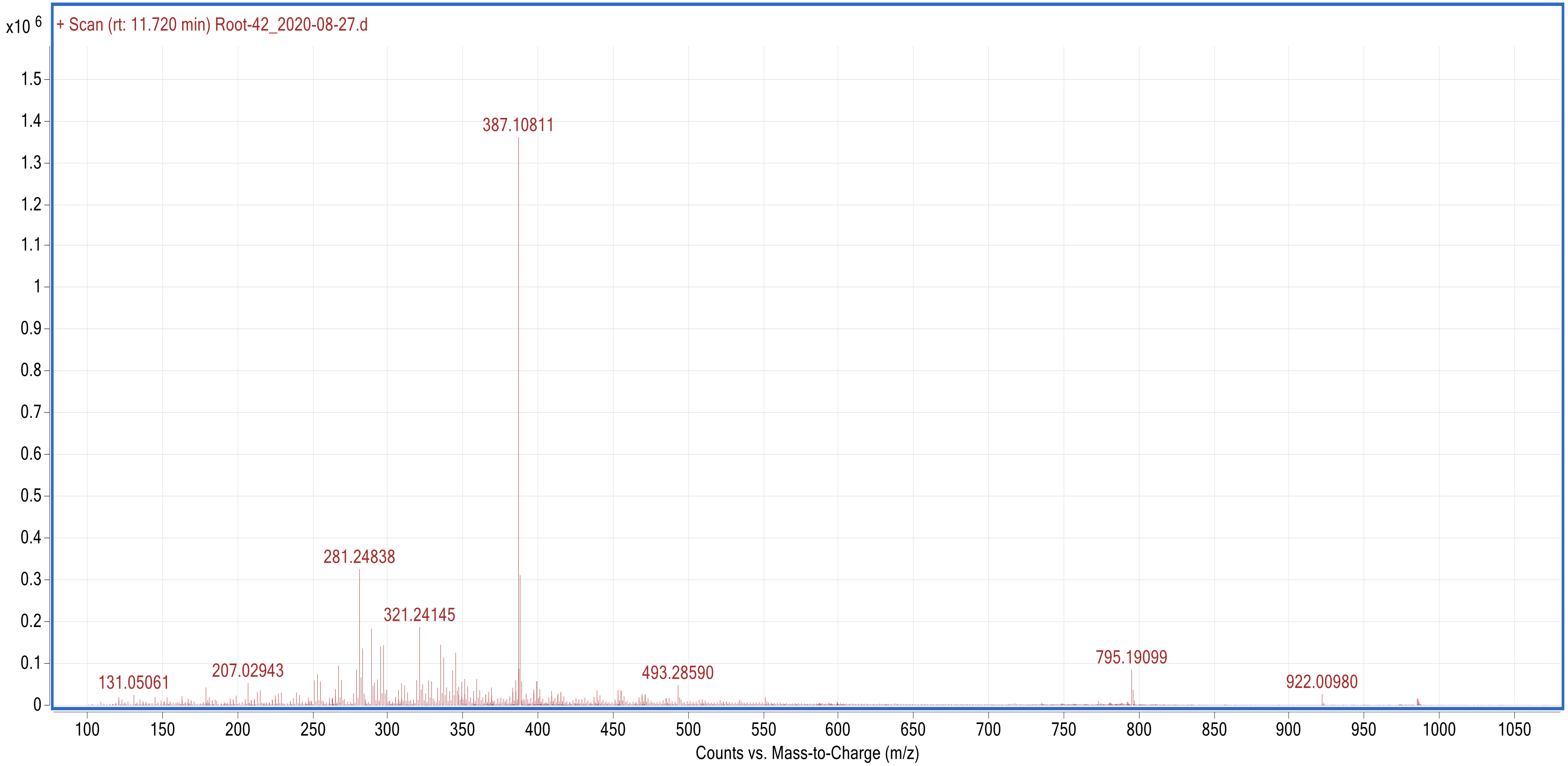

Figure S1. HRESI-MS spectrum of the new compound **1a**

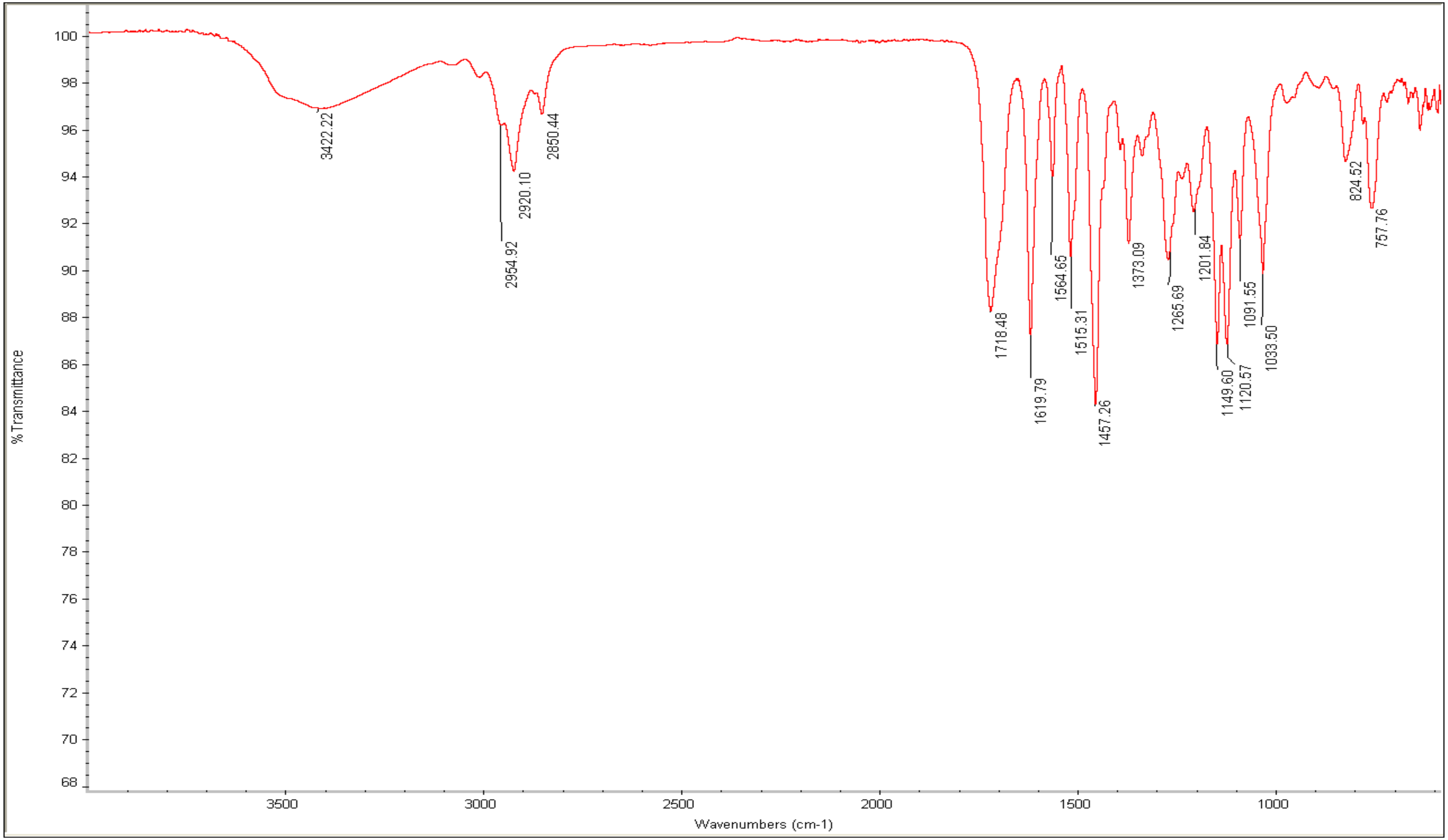

Figure S2. IR spectrum of the new compound **1a**

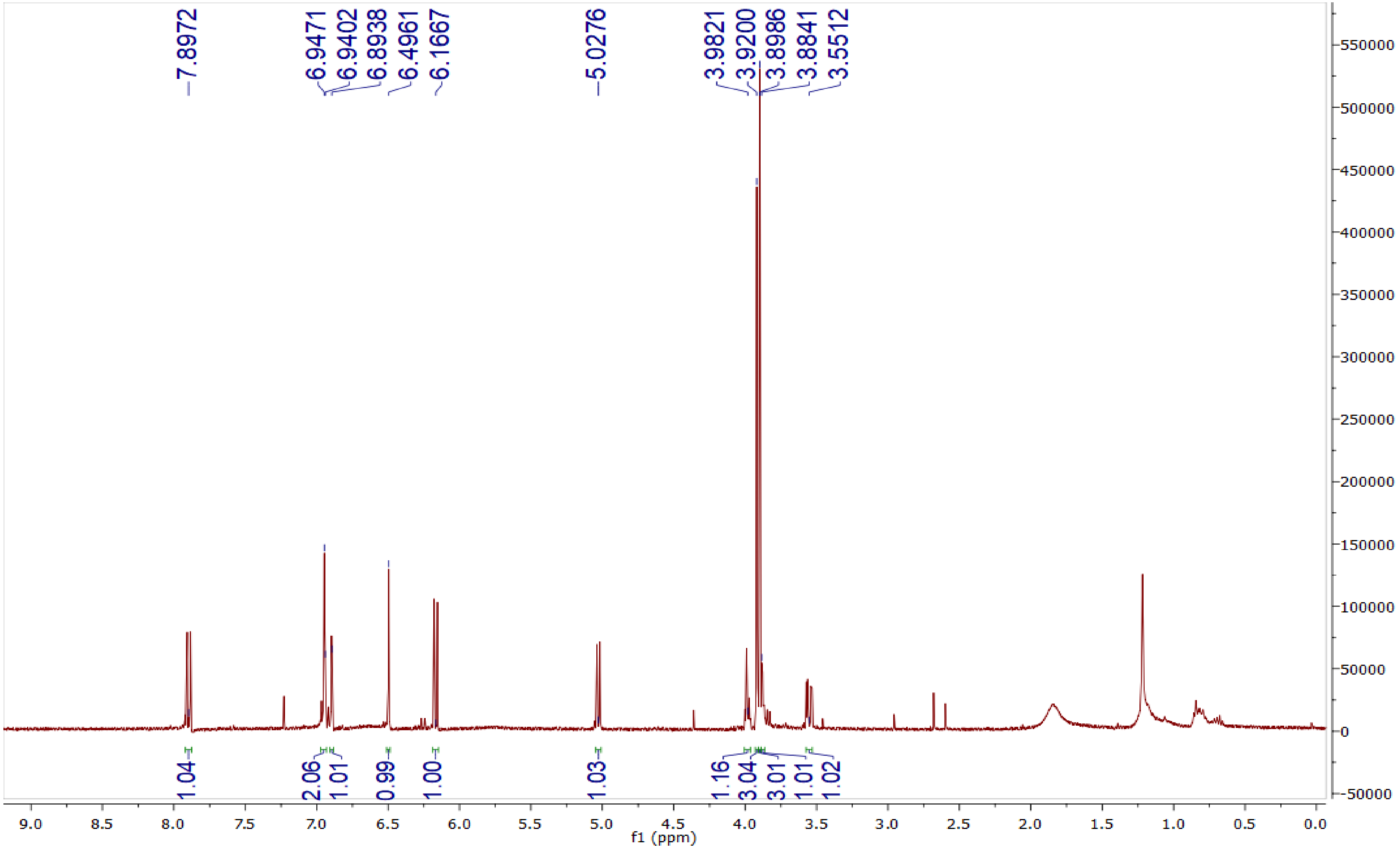

Figure S3. ^{1}H NMR (400 MHz, $CDCl_3$) spectrum of the new compound **1a**

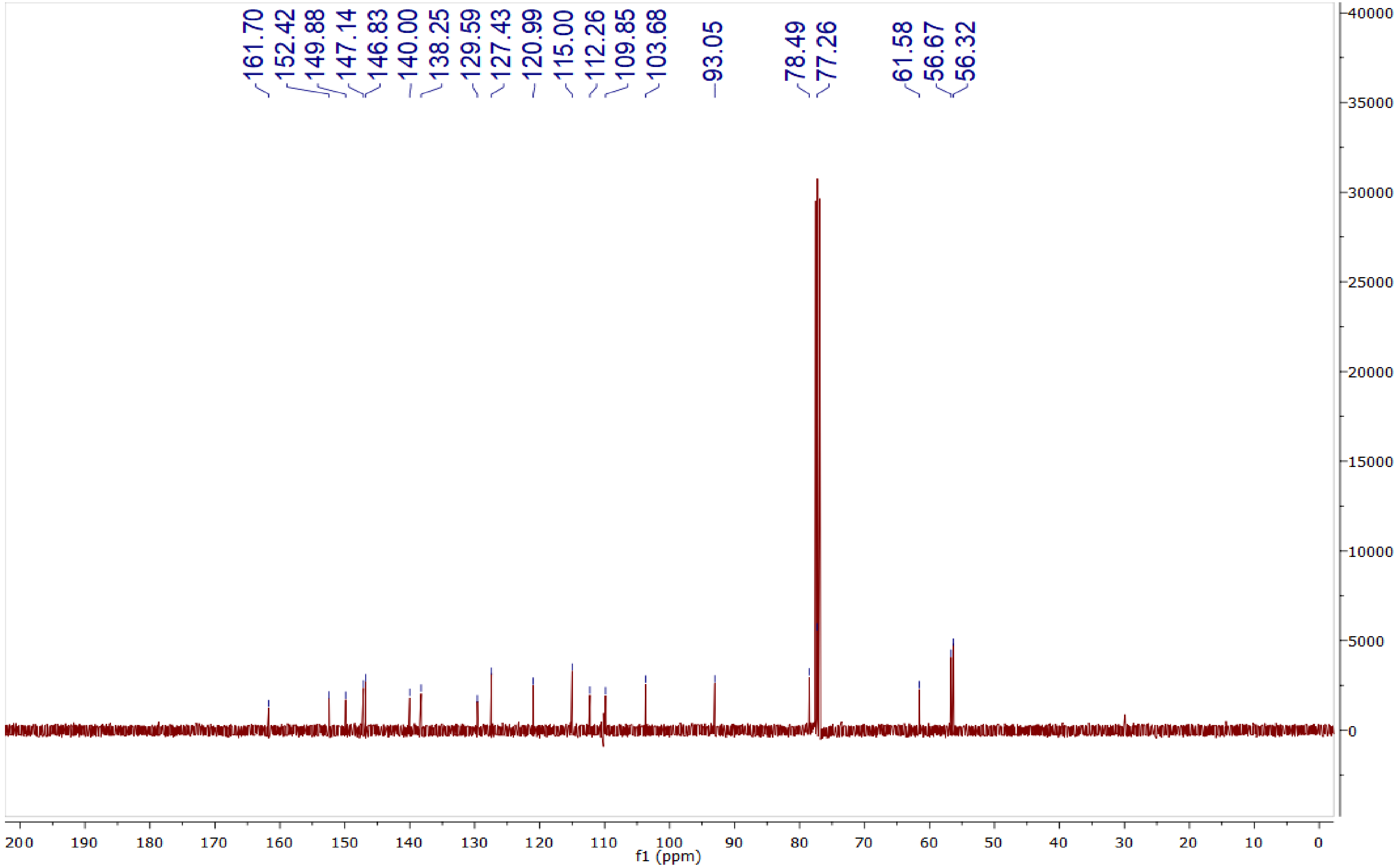

Figure S4. ^{13}C NMR (100 MHz, $CDCl_3$) spectrum of the new compound **1a**

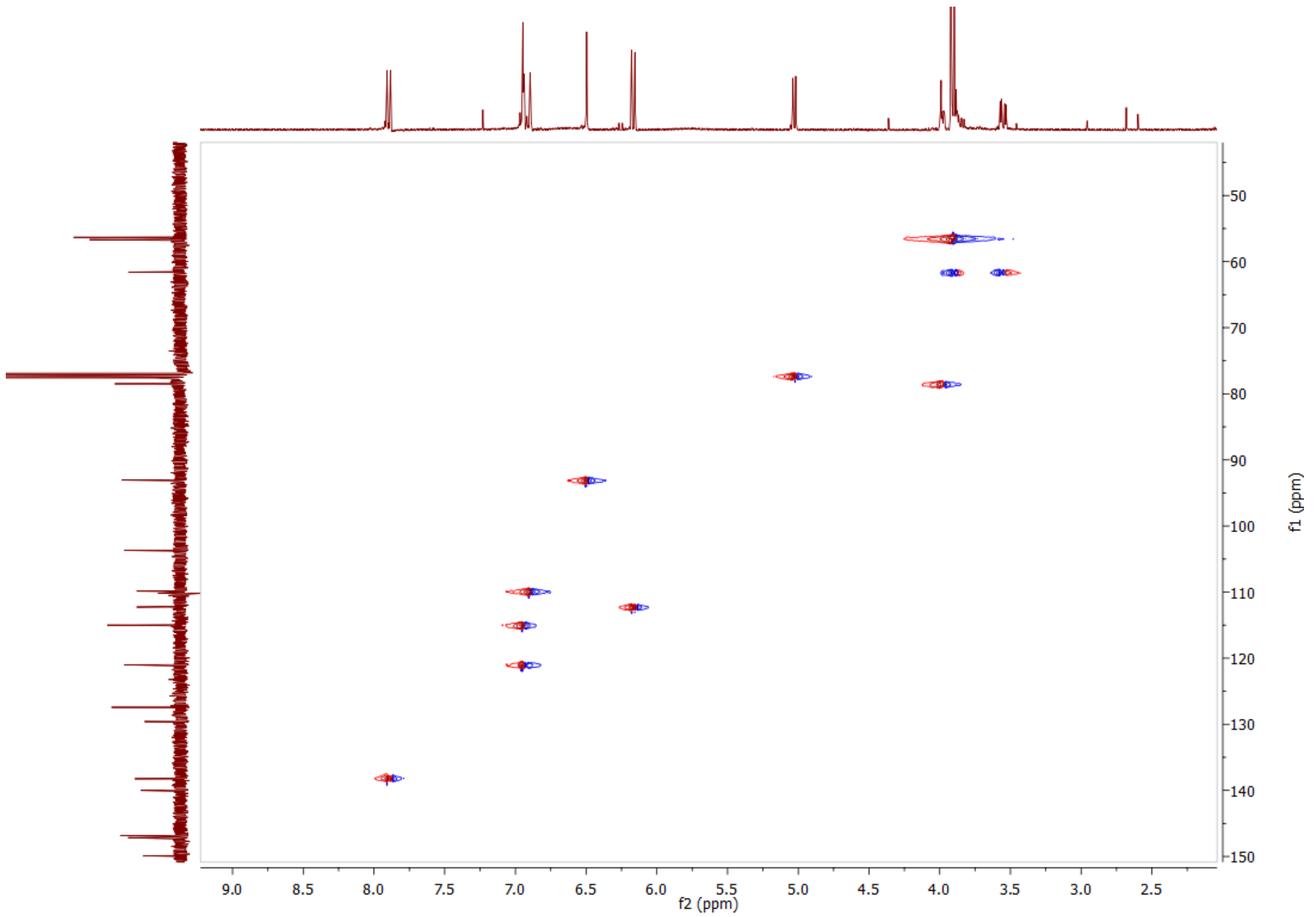

Figure S5. HSQC spectrum of the new compound **1a**

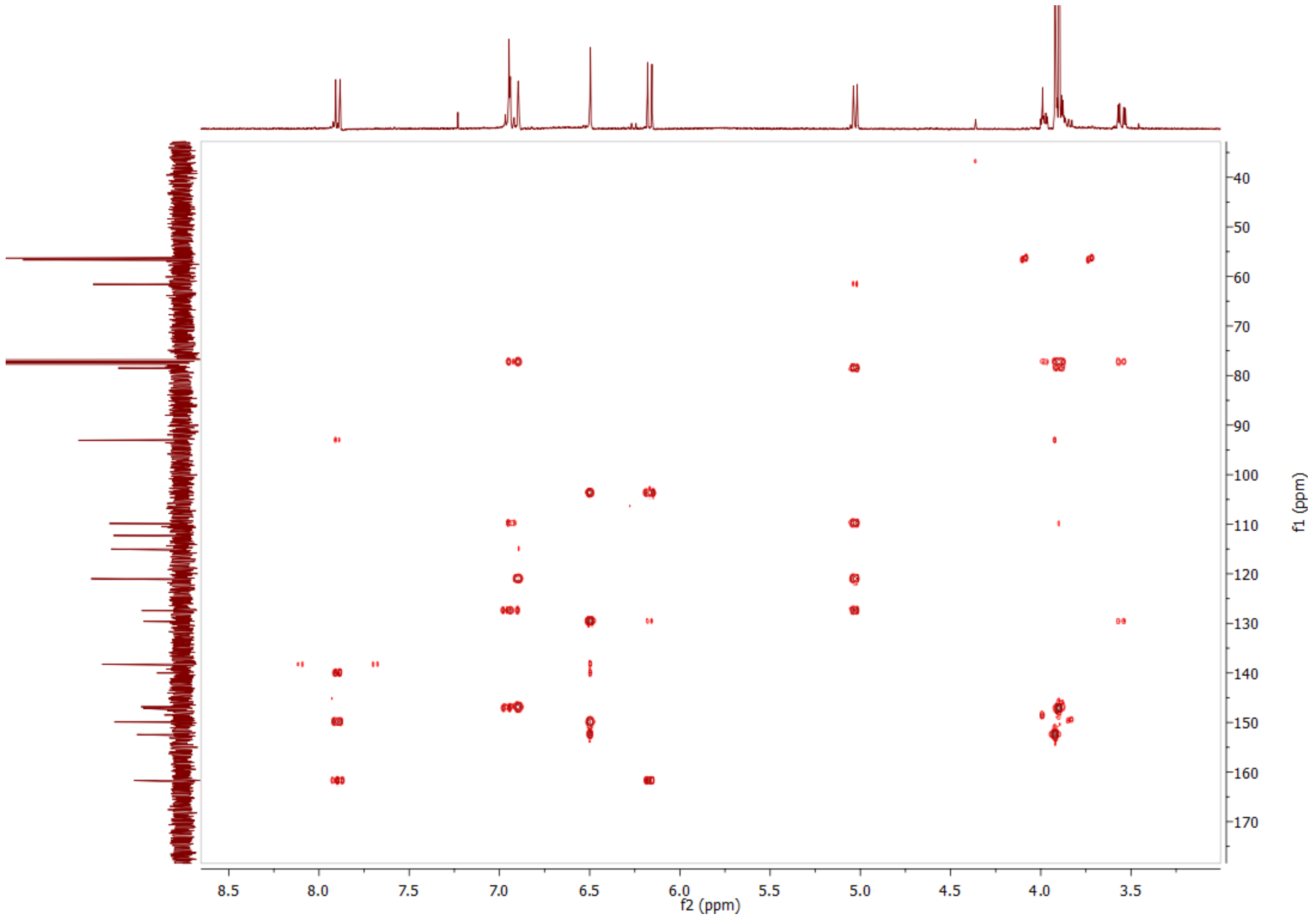

Figure S6. HMBC spectrum of the new compound **1a**

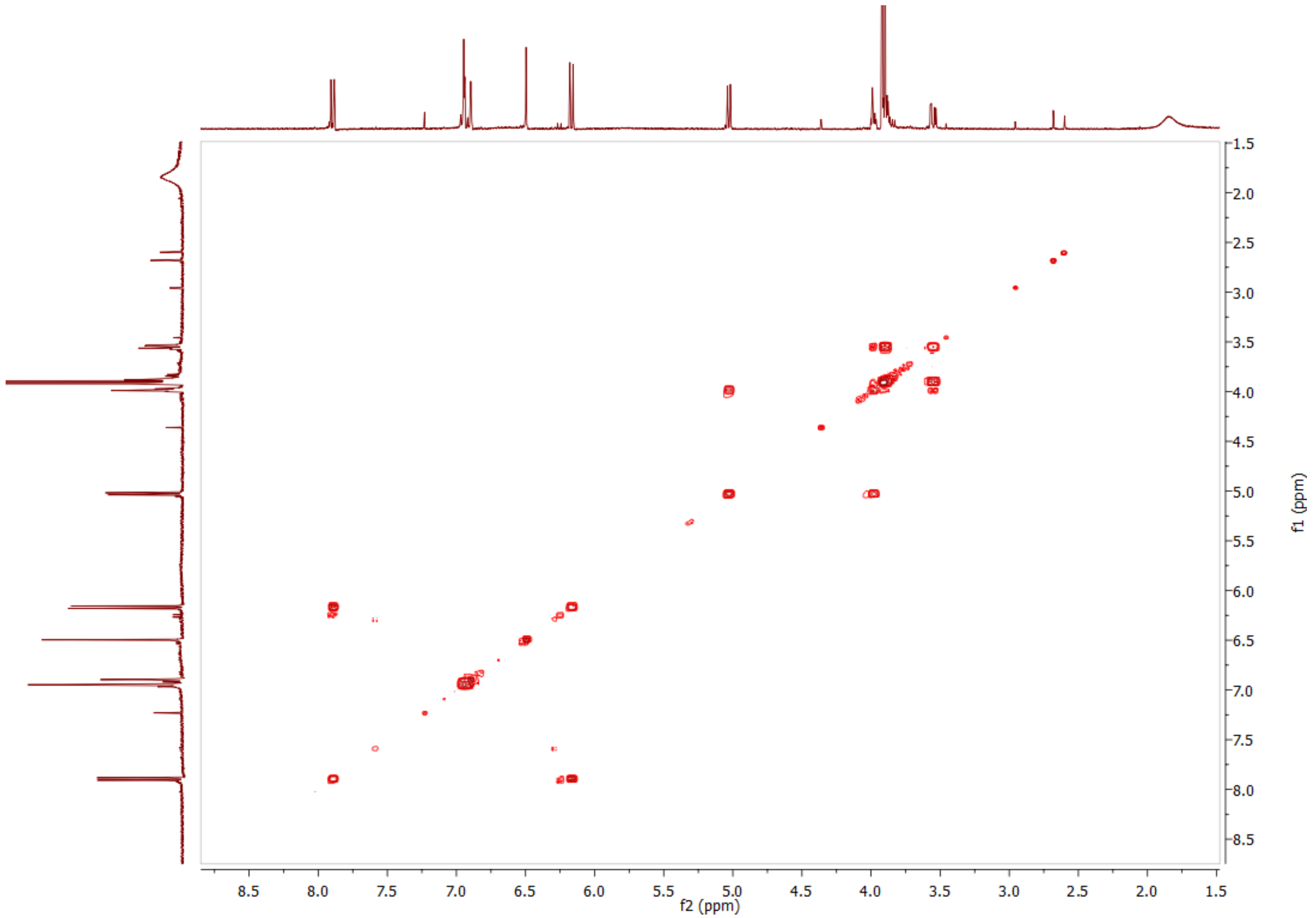

Figure S7. COSY spectrum of the new compound **1a**

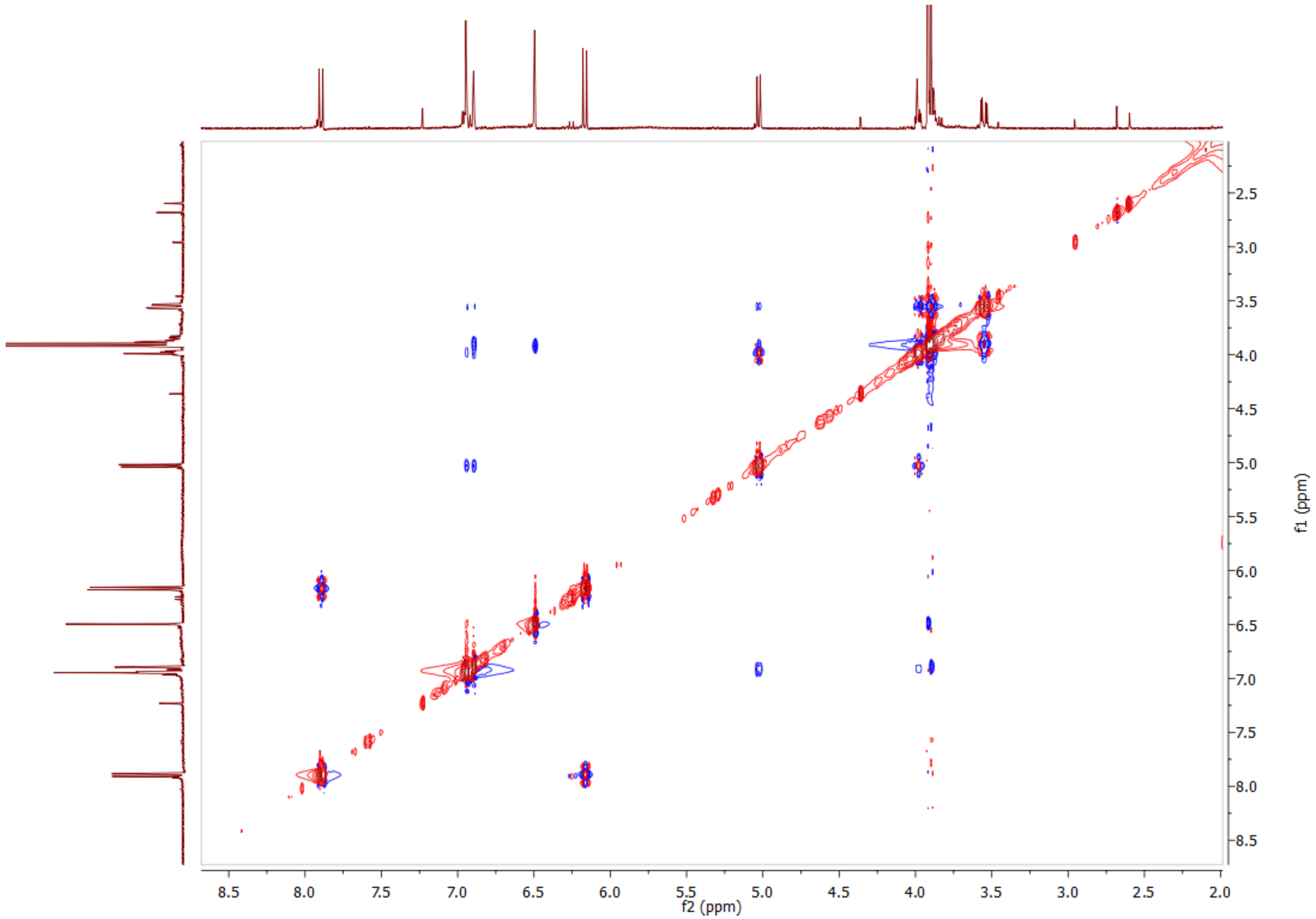

Figure S8. NOESY spectrum of the new compound **1a**

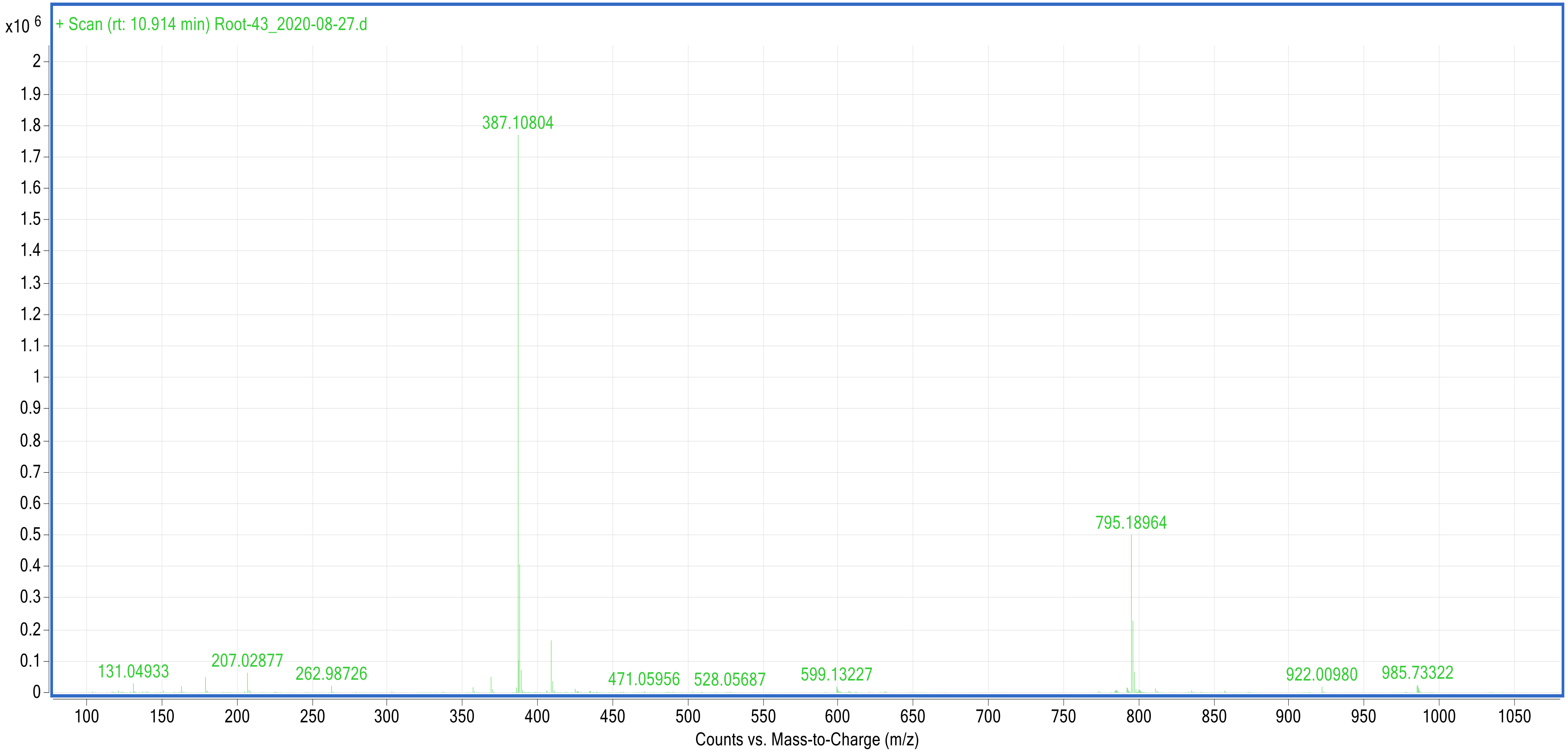

Figure S9. HRESI-MS spectrum of the new compound **1b**

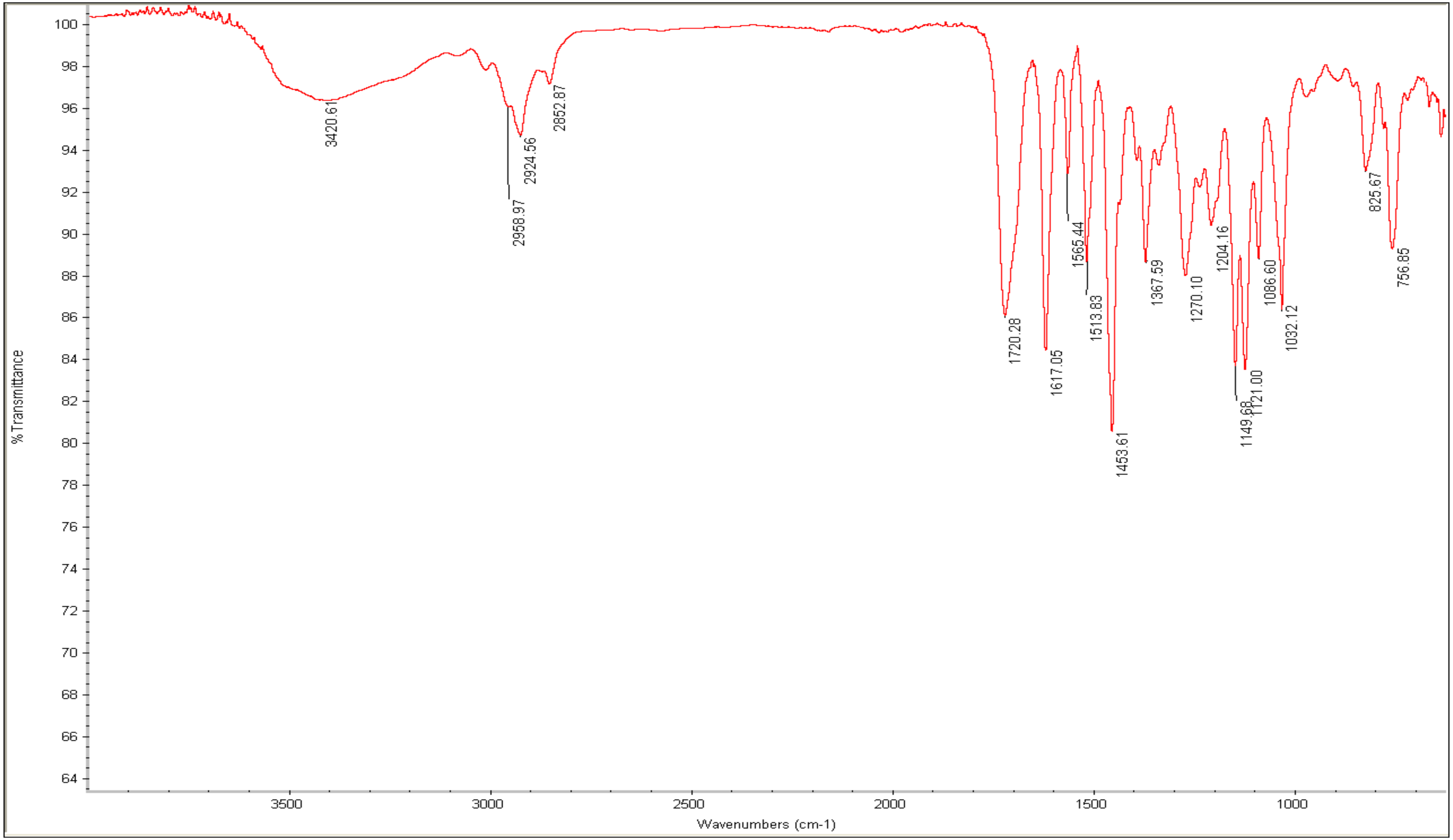

Figure S10. IR spectrum of the new compound **1b**

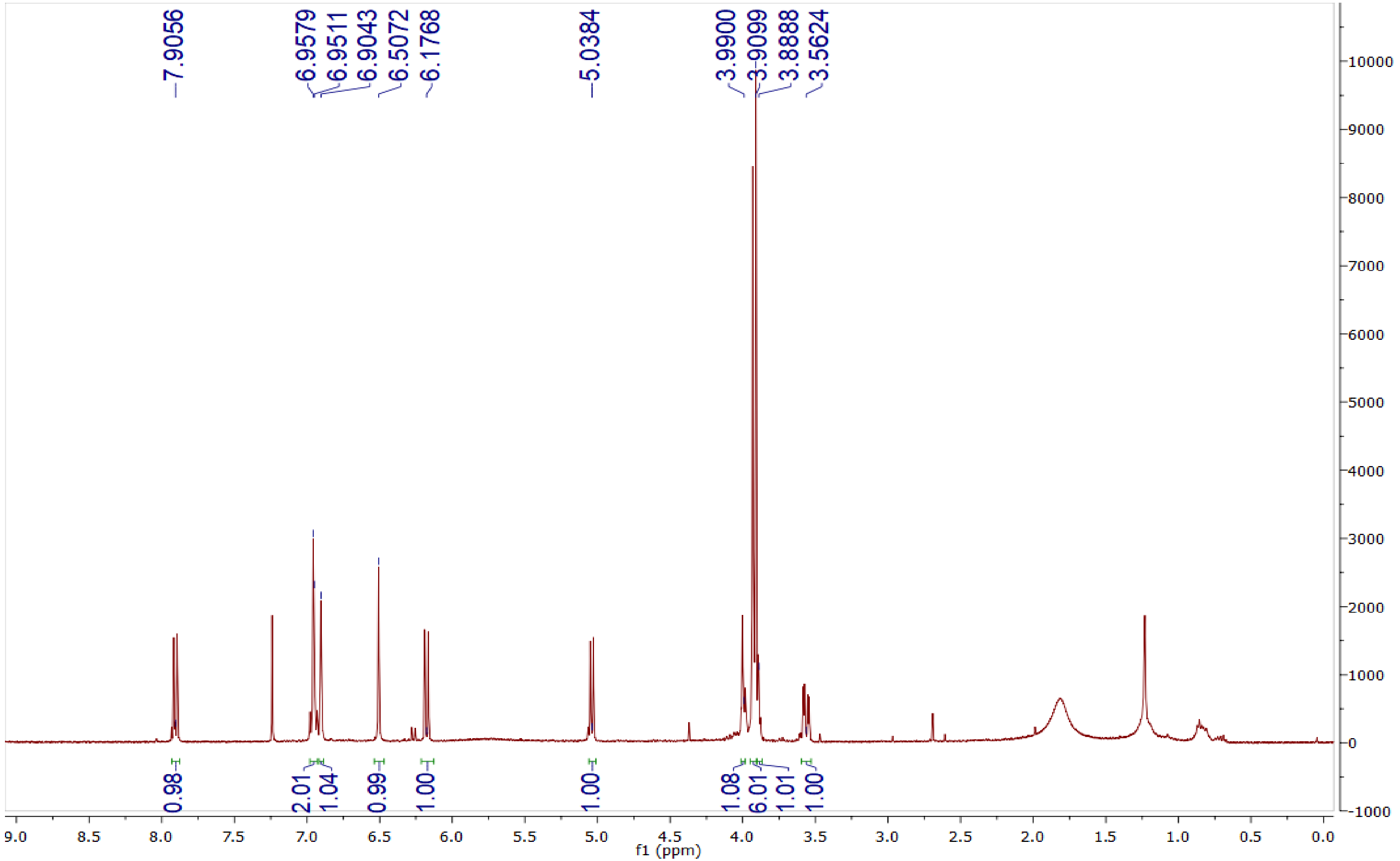

Figure S11. ^{1}H NMR (400 MHz, $CDCl_3$) spectrum of the new compound **1b**

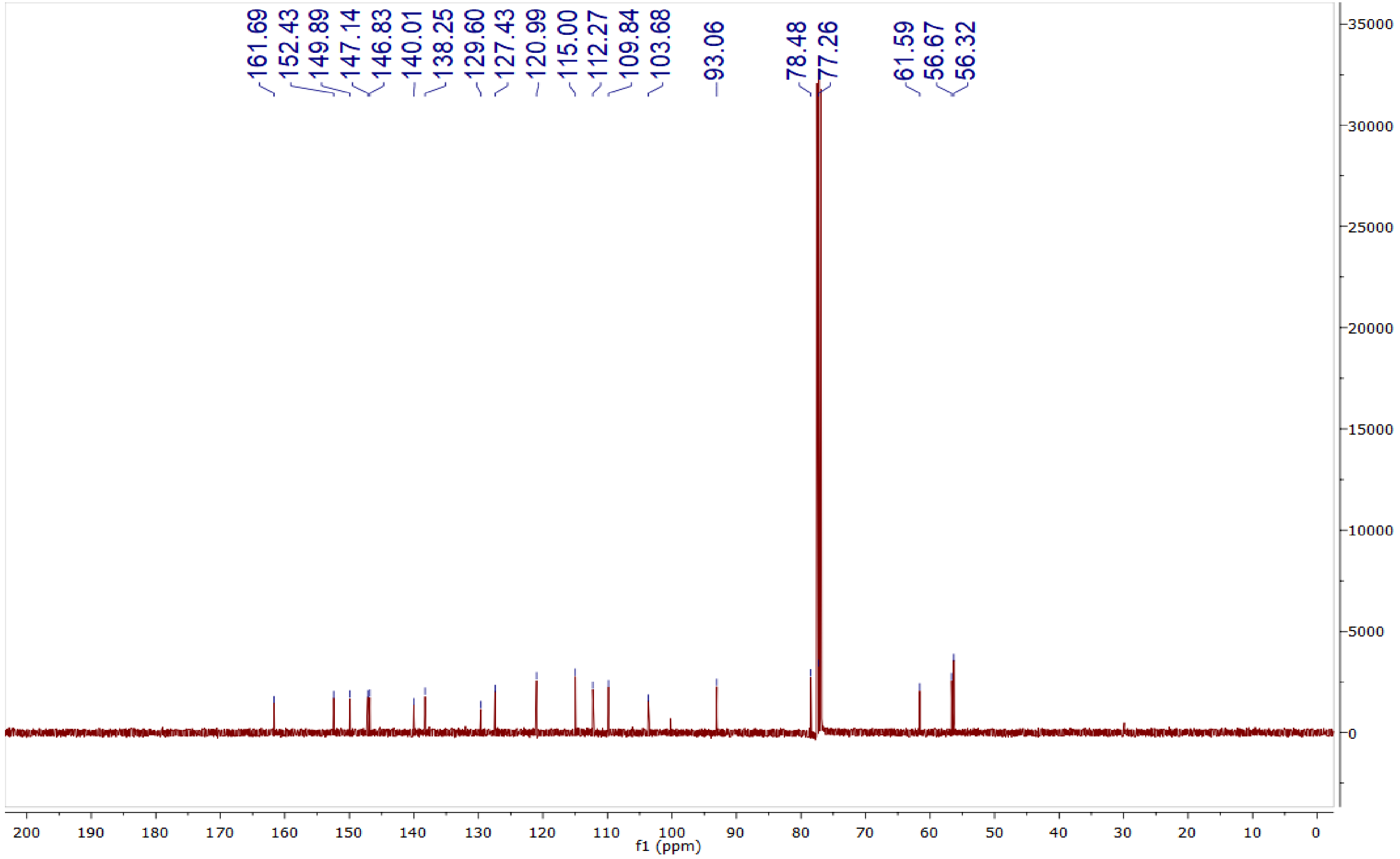

Figure S12. ^{13}C NMR (100 MHz, $CDCl_3$) spectrum of the new compound **1b**

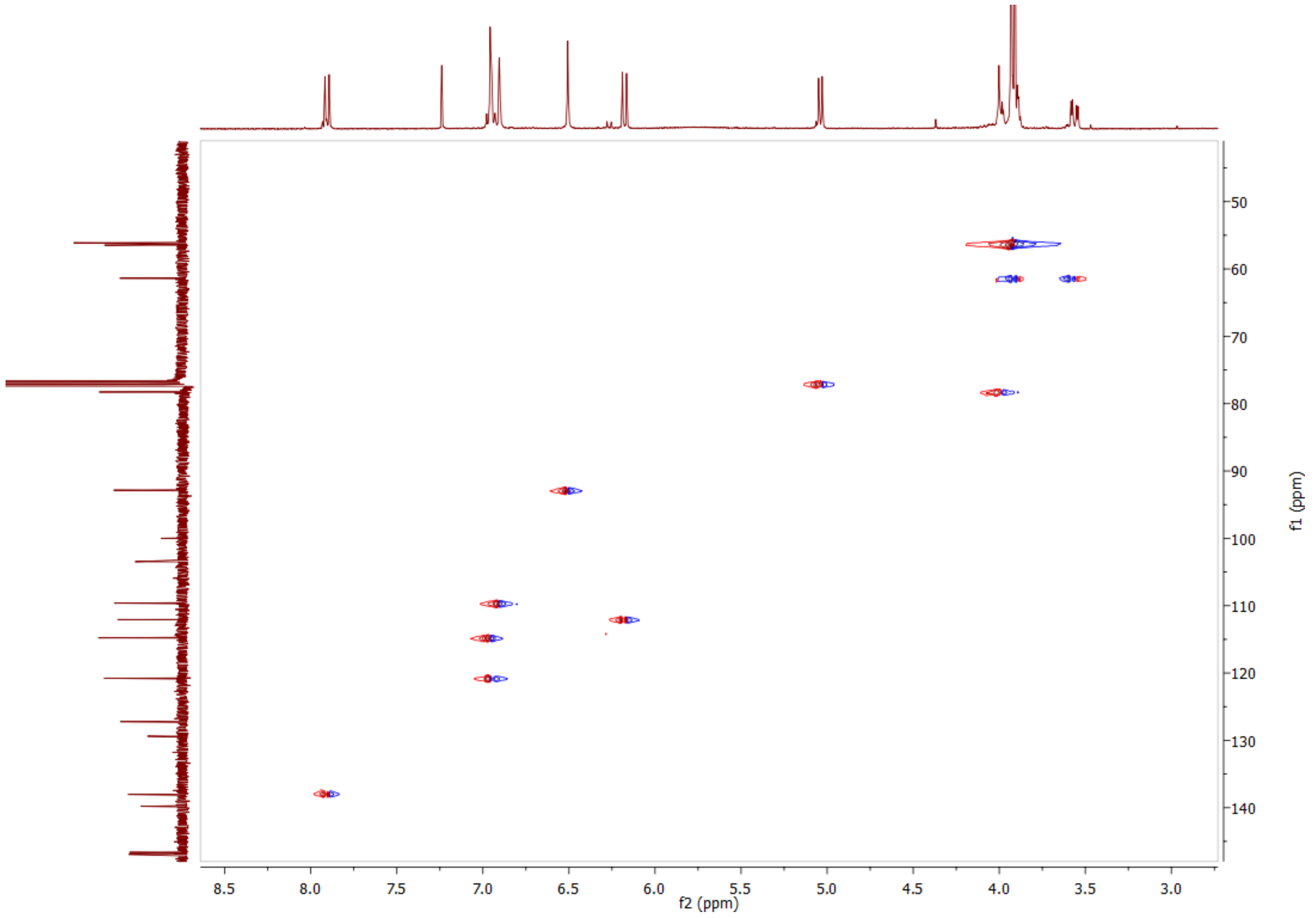

Figure S13. HSQC spectrum of the new compound **1b**

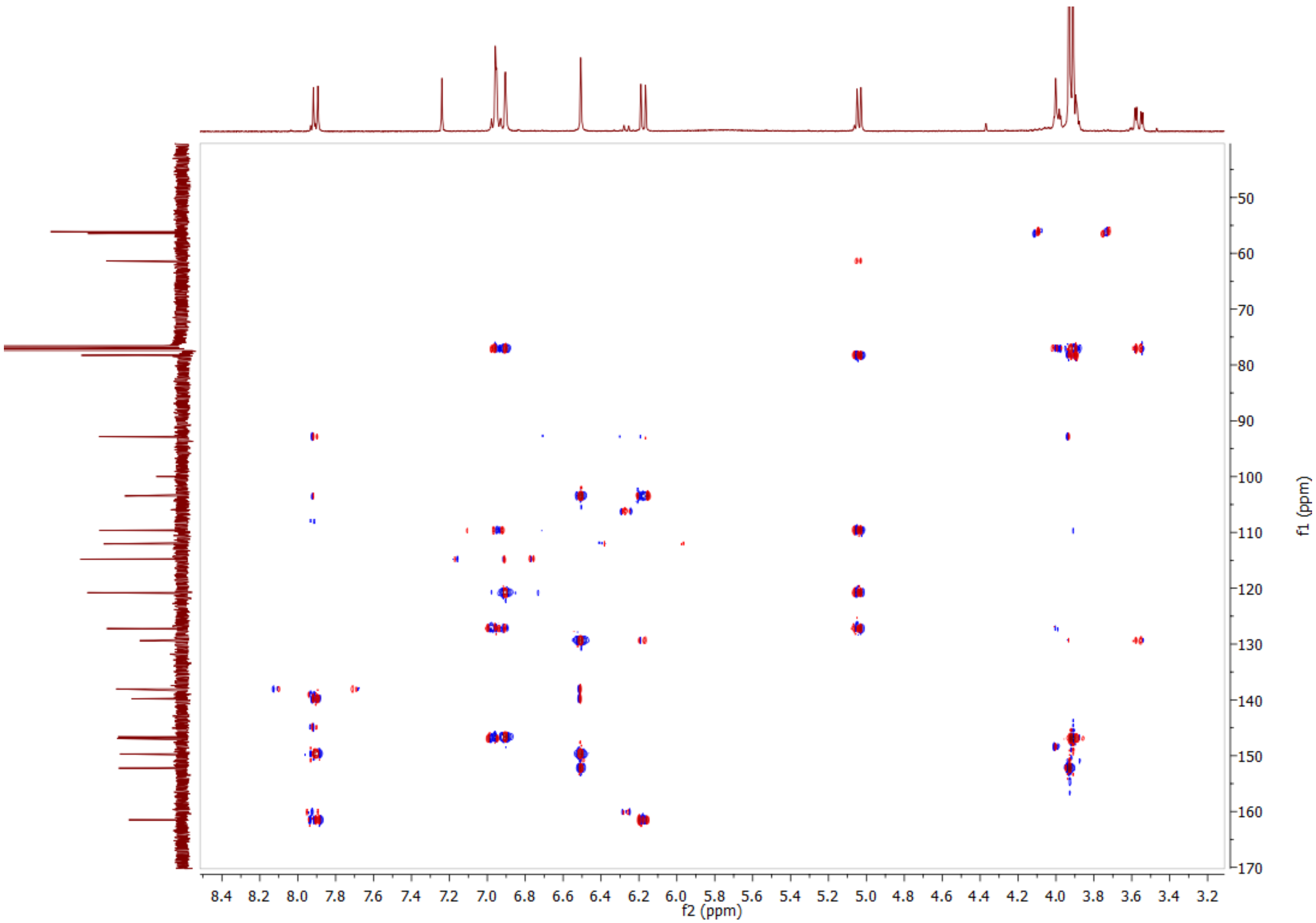

Figure S14. HMBC spectrum of the new compound **1b**

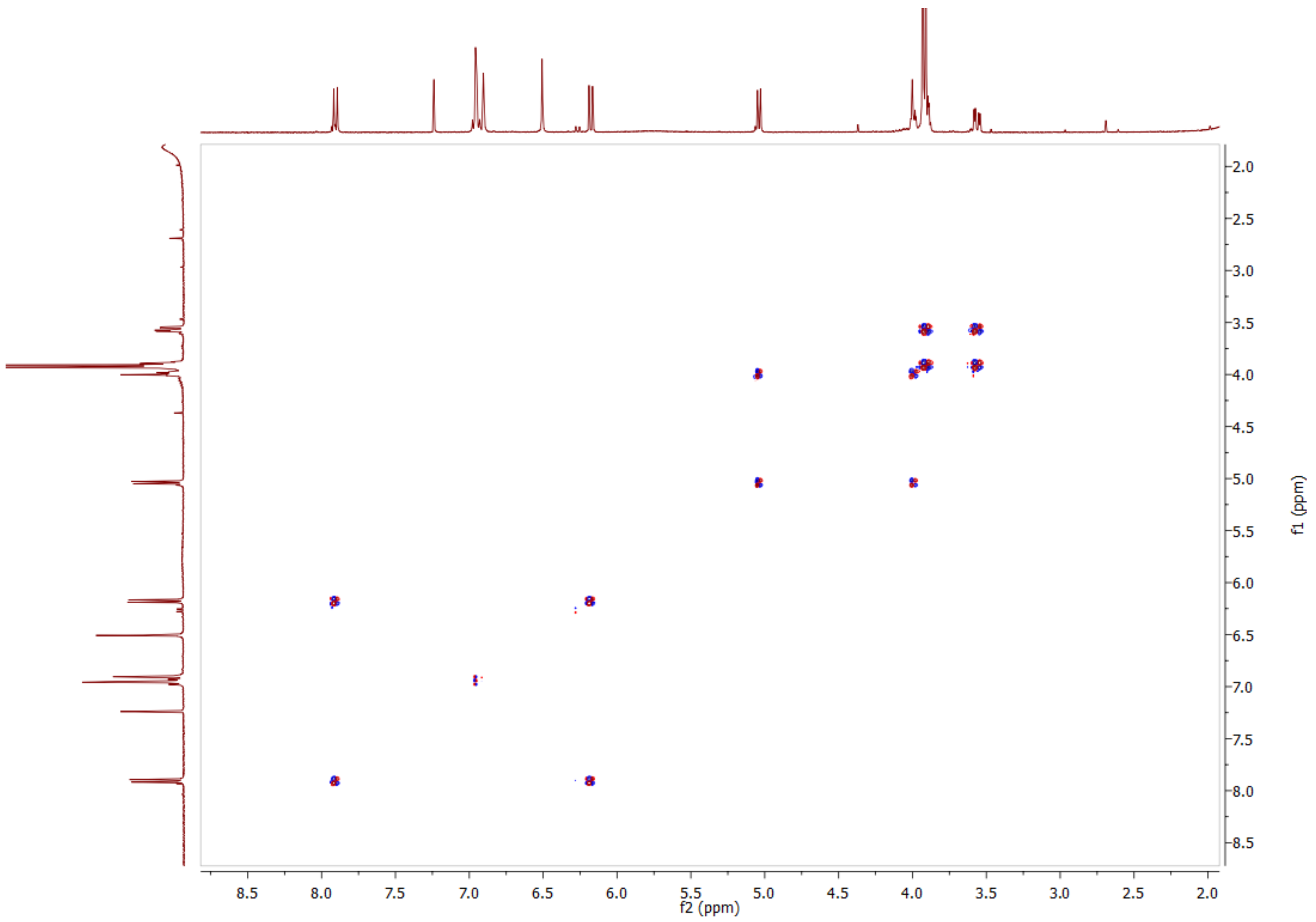

Figure S15. COSY spectrum of the new compound **1b**

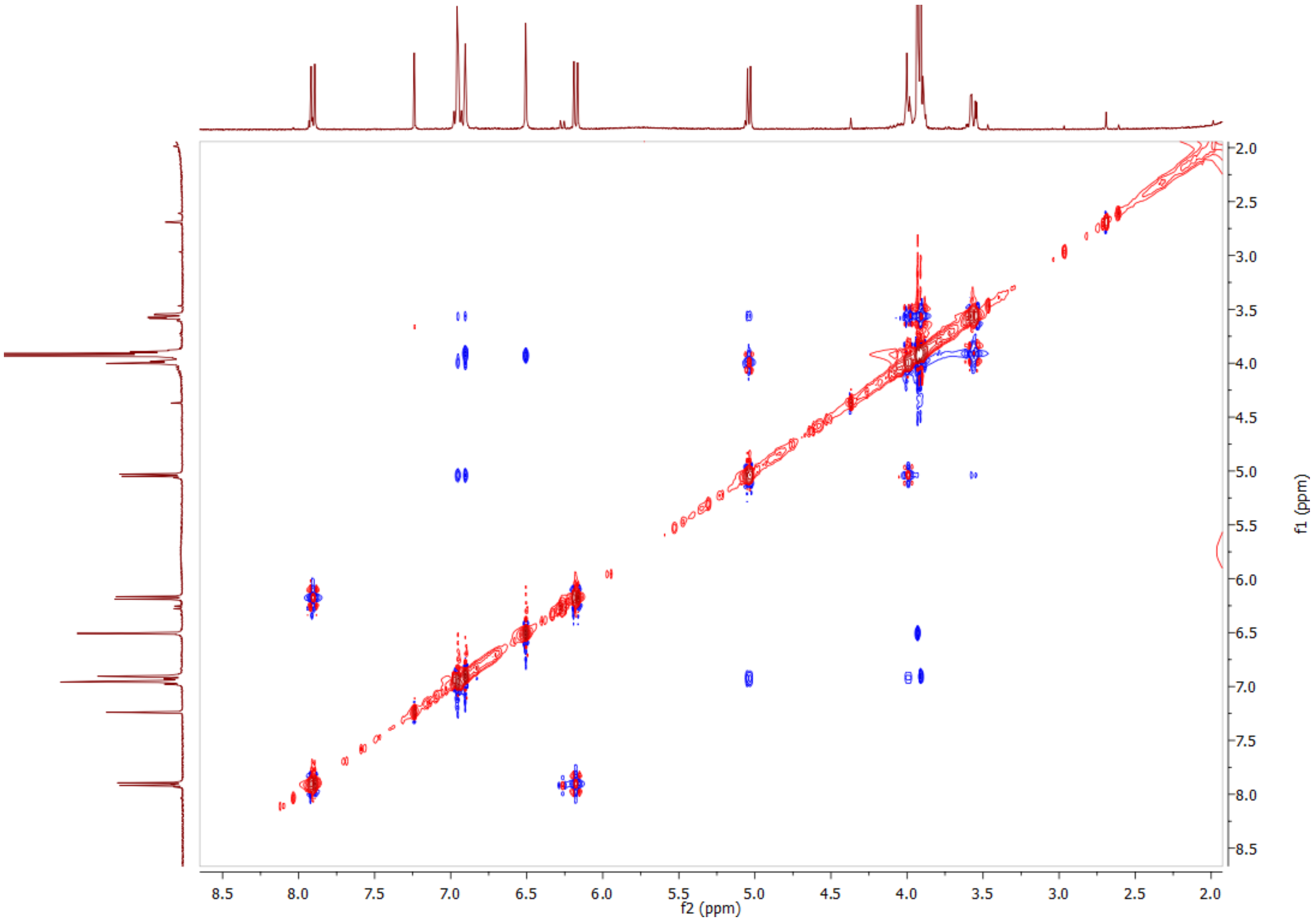

Figure S16. NOESY spectrum of the new compound **1b**

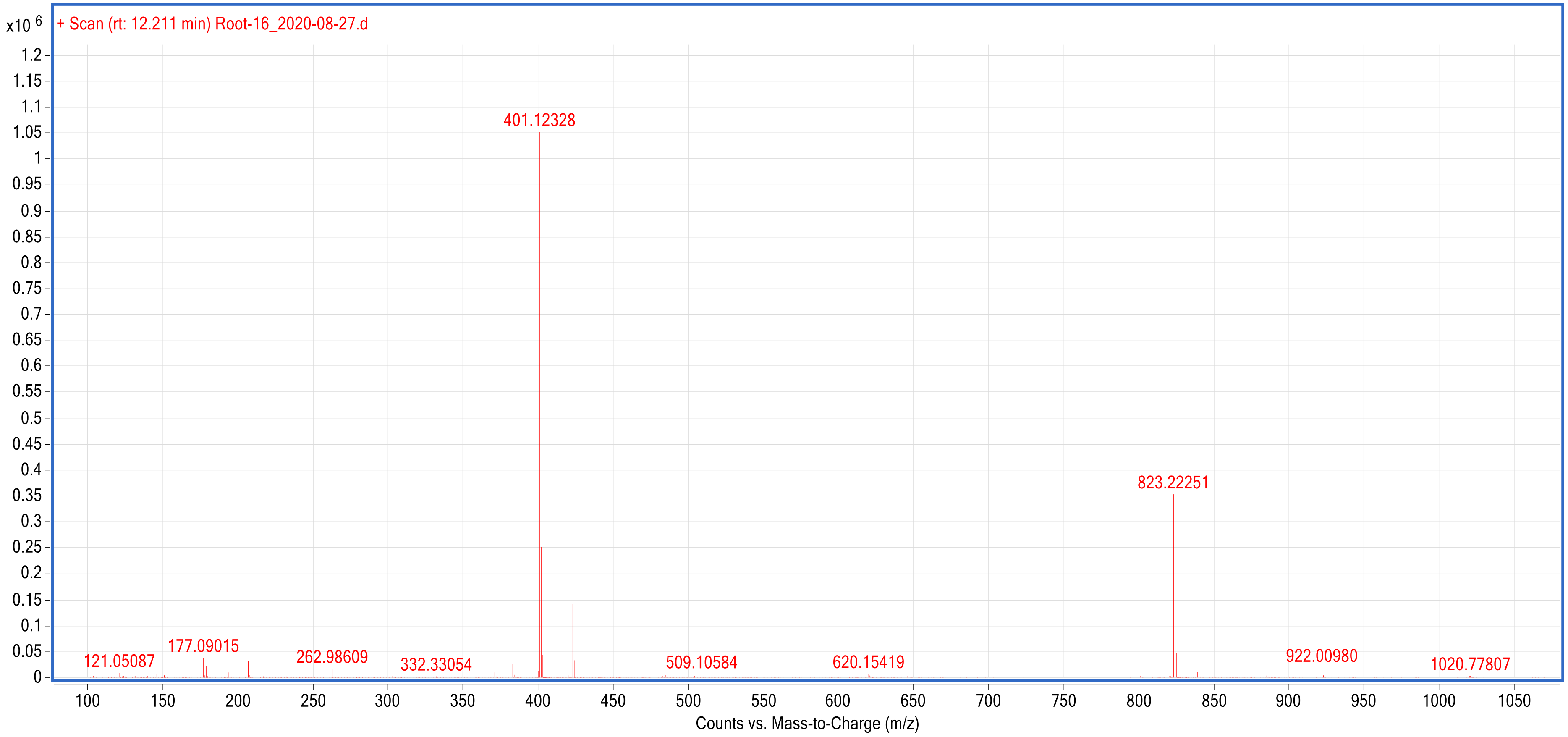

Figure S17. HRESI-MS spectrum of the new compound **2**

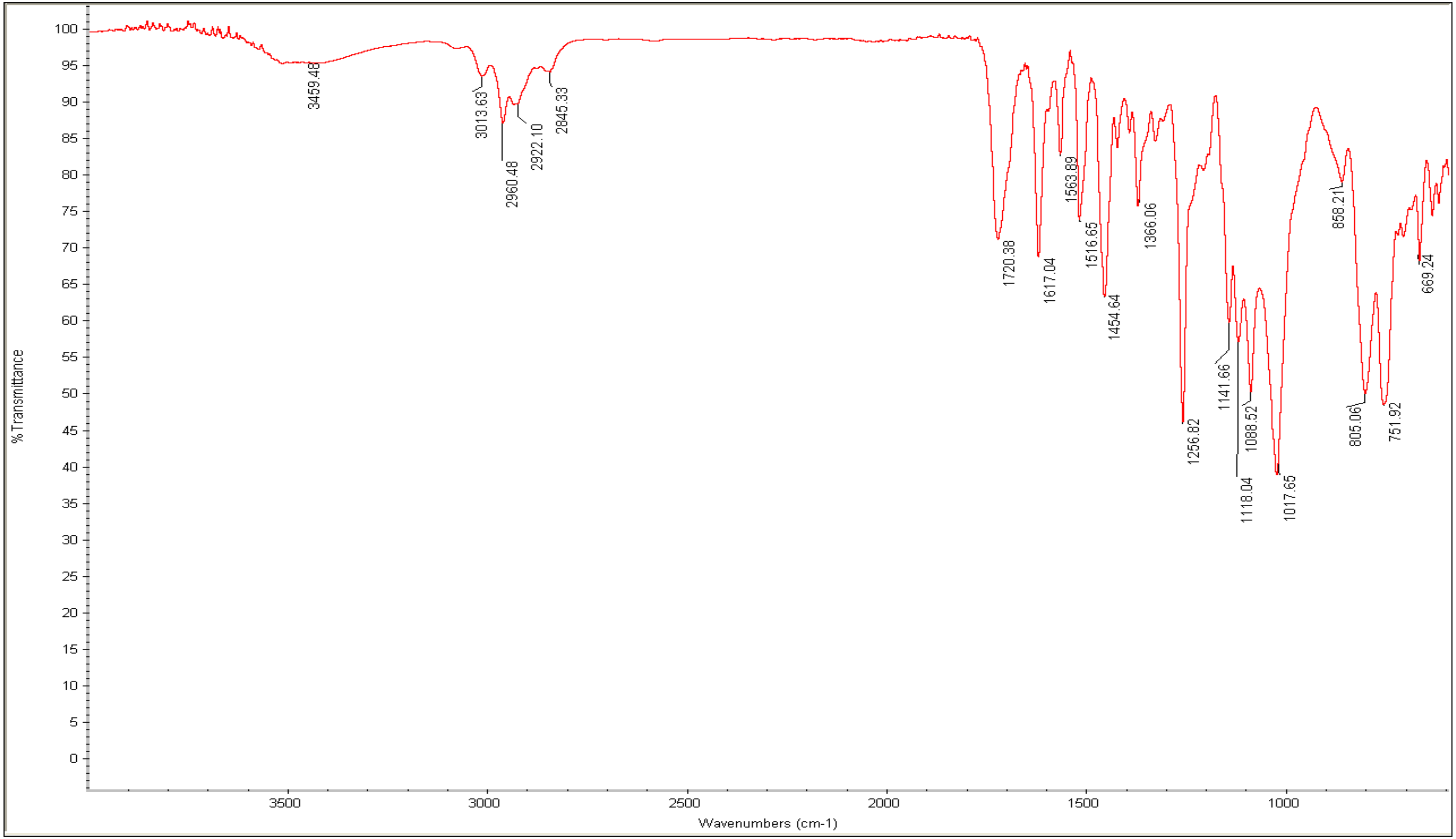

Figure S18. IR spectrum of the new compound **2**

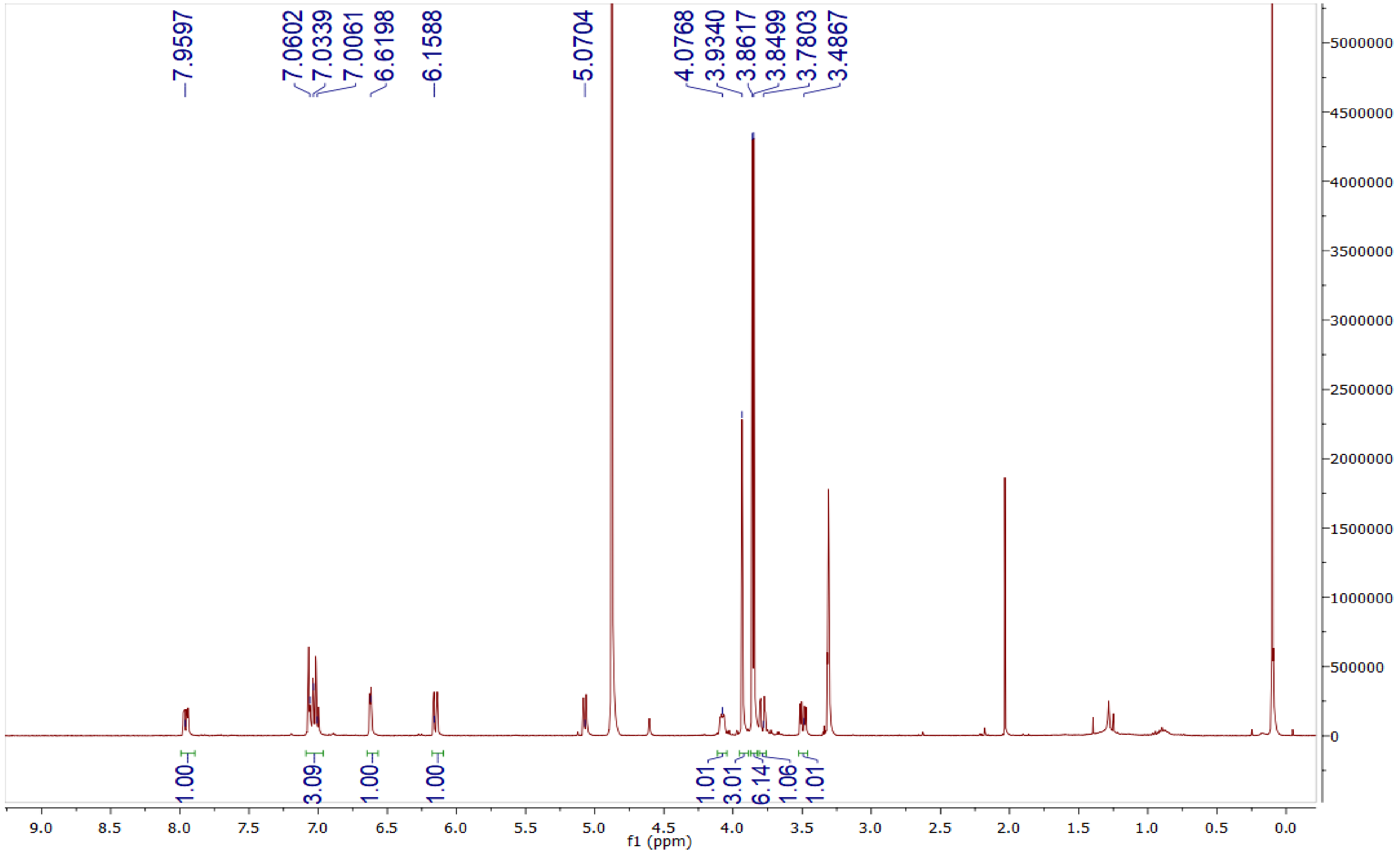

Figure S19. ^{1}H NMR (400 MHz, MeOD) spectrum of the new compound **2**

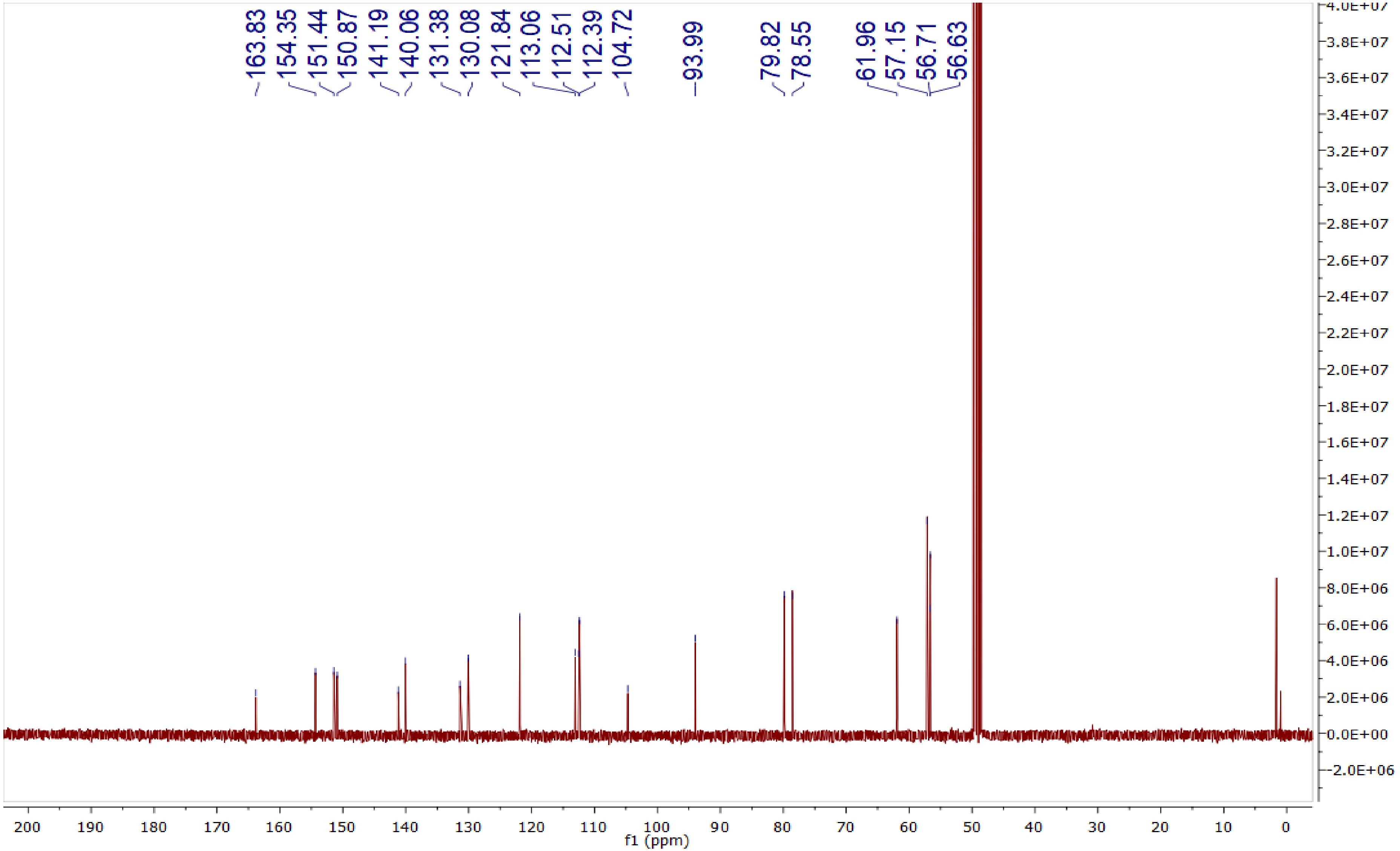

Figure S20. ^{13}C NMR (100 MHz, MeOD) spectrum of the new compound **2**

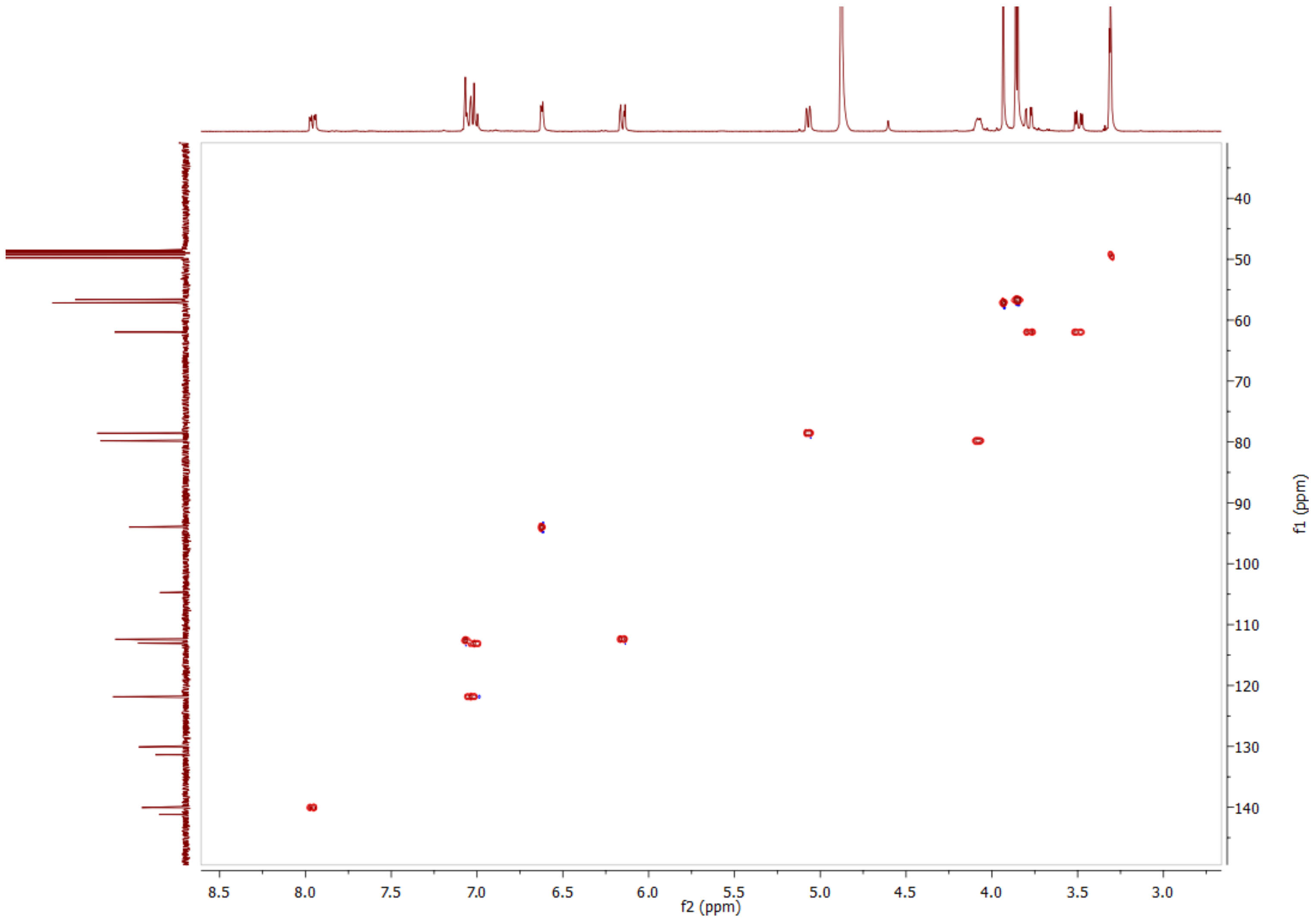

Figure S21. HSQC spectrum of the new compound **2**

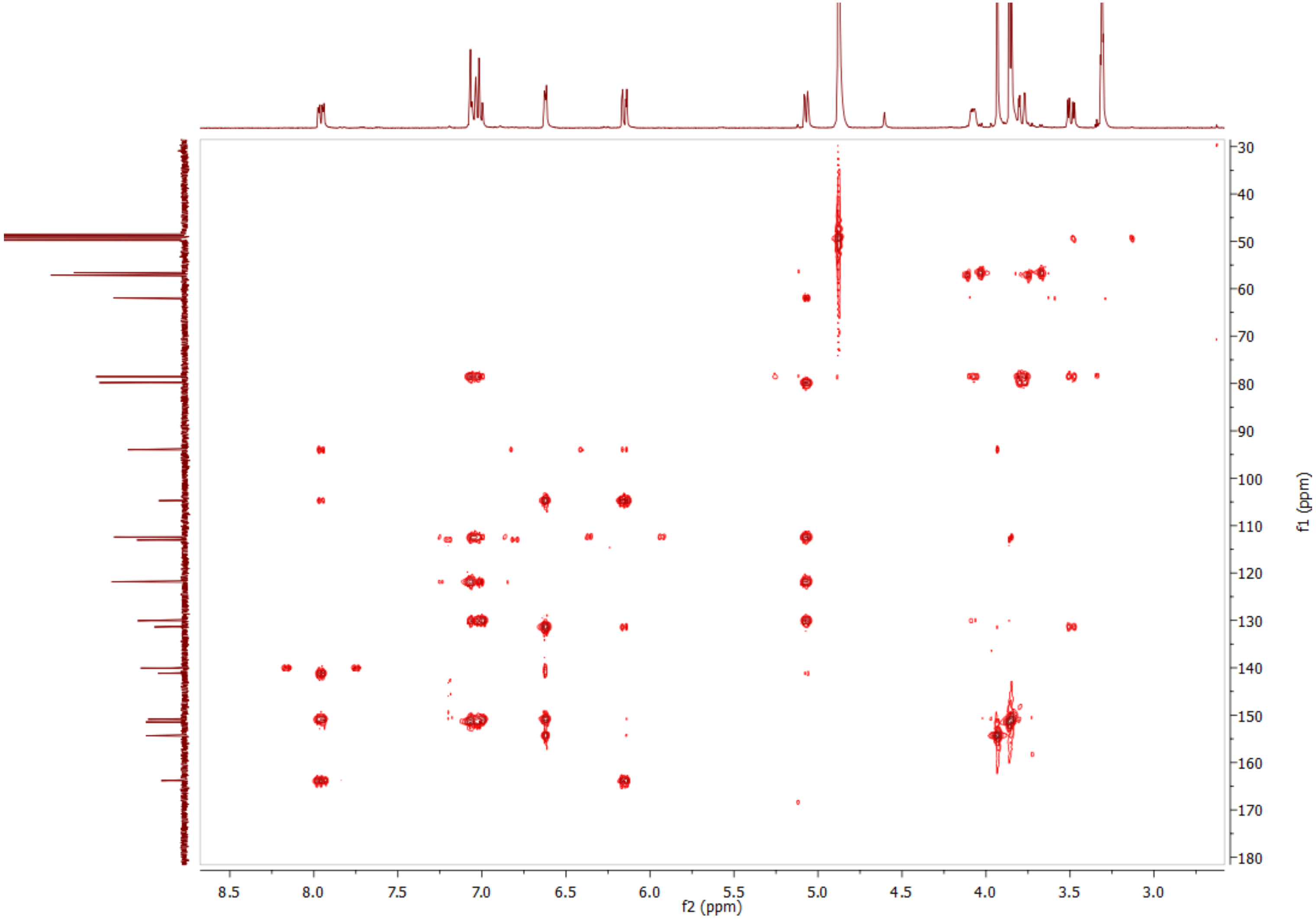

Figure S22. HMBC spectrum of the new compound **2**

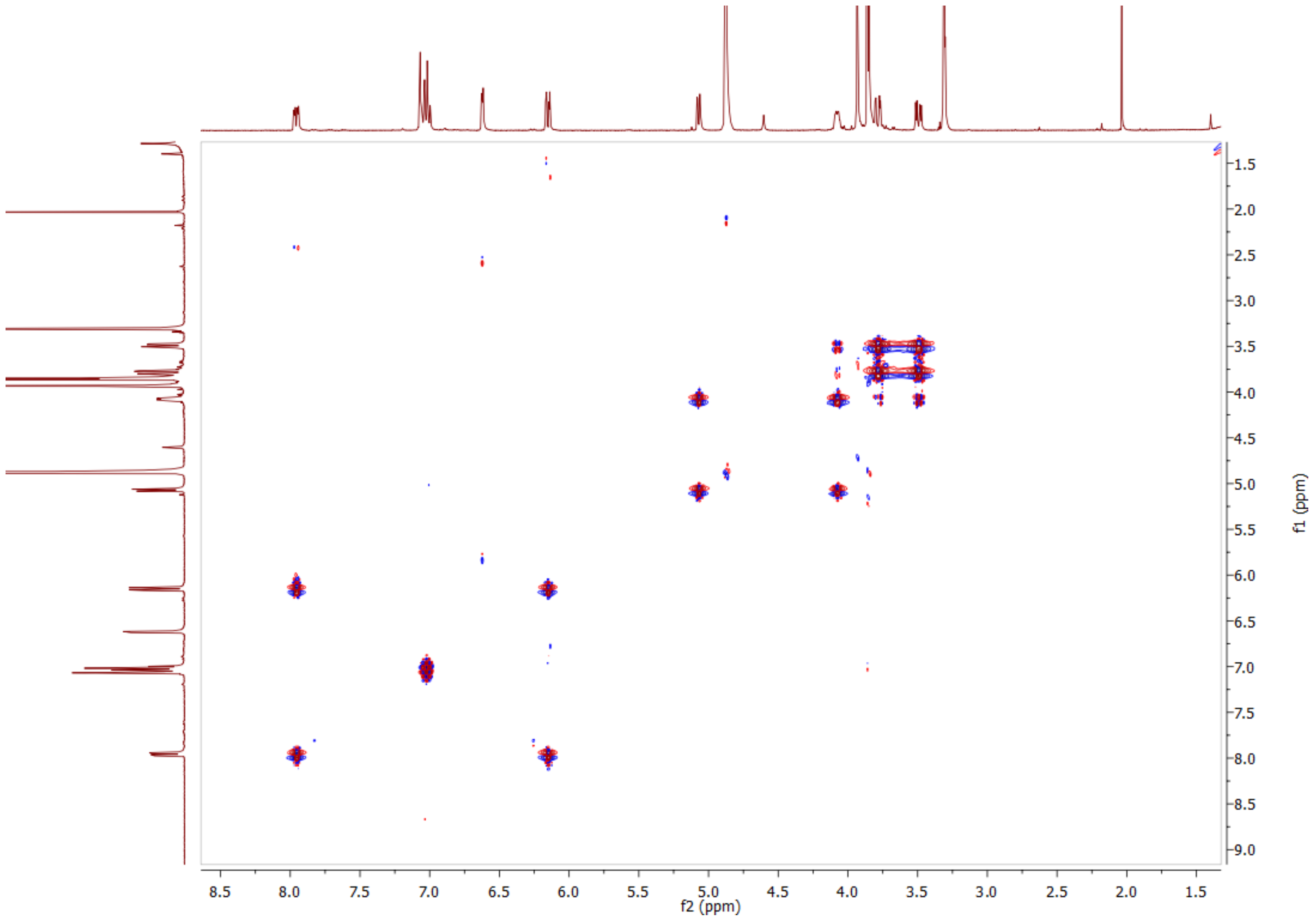

Figure S23. COSY spectrum of the new compound **2**

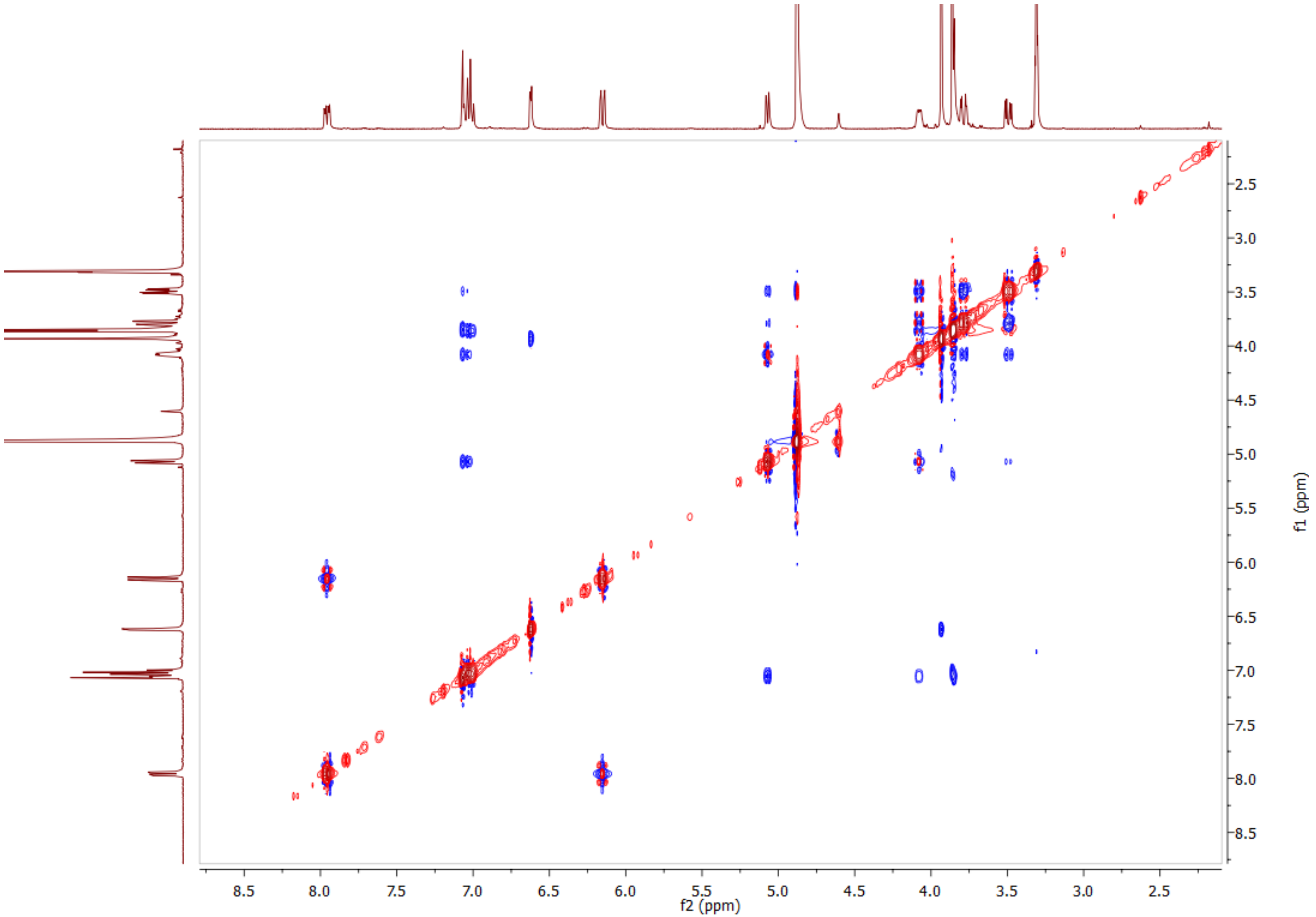

Figure S24. NOESY spectrum of the new compound **2**

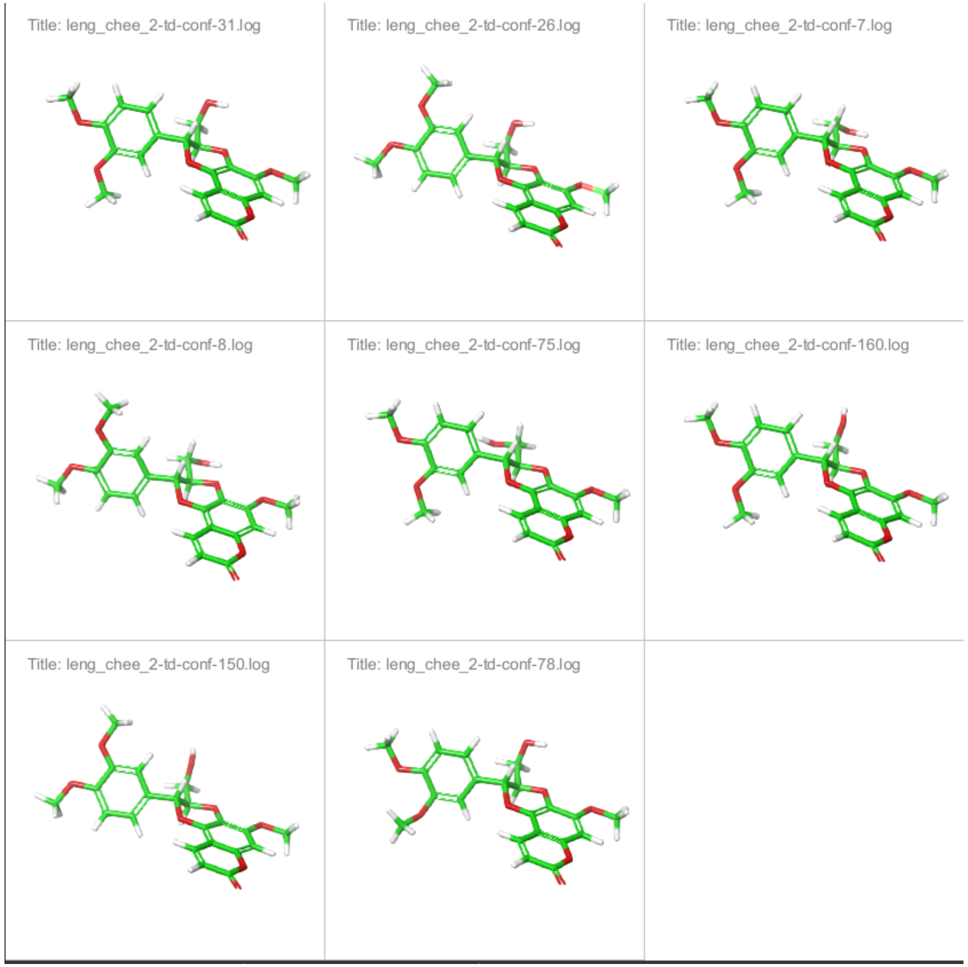

Figure S25. Images of conformers (>1%) for the new compound **2**

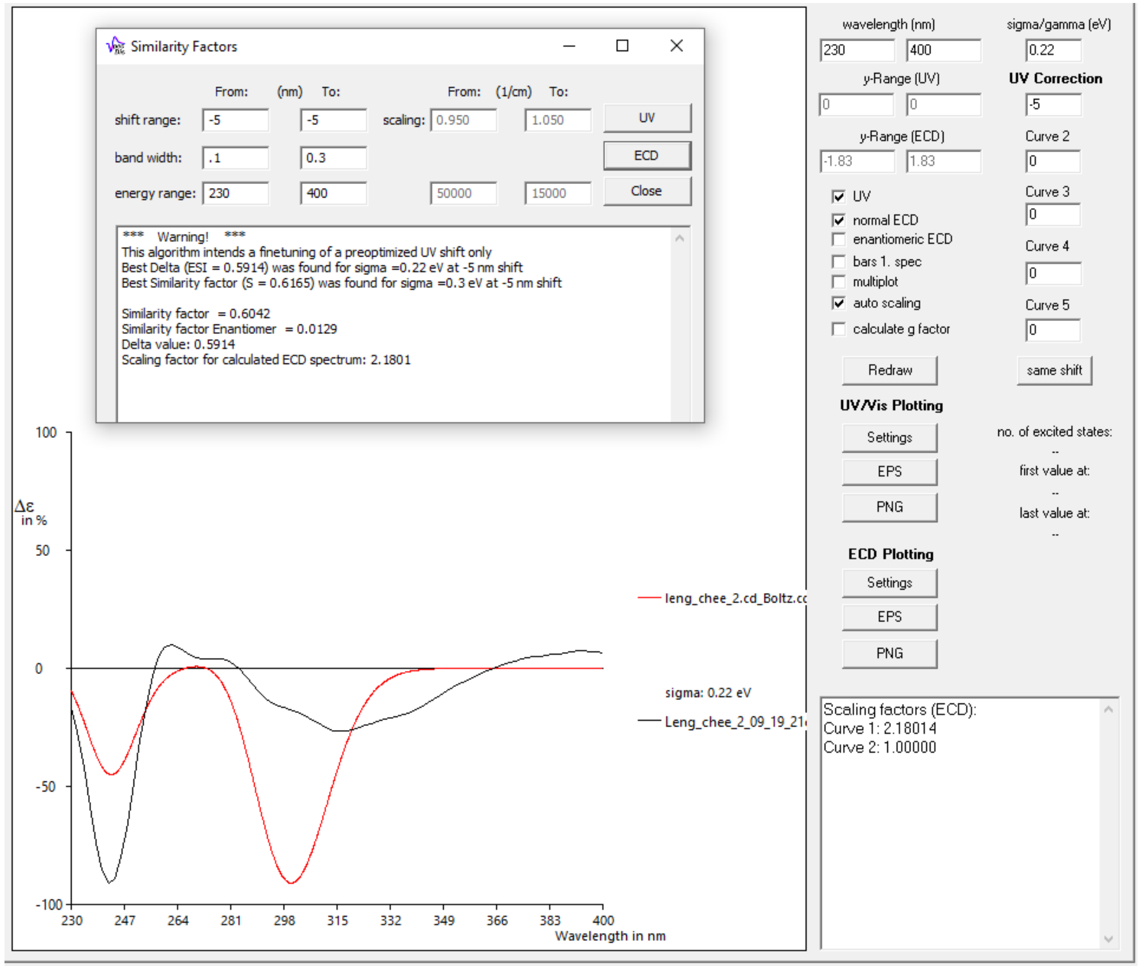

Figure S26. CD spectrum (experimental to calculated comparison by SpecDis) of the new compound **2**

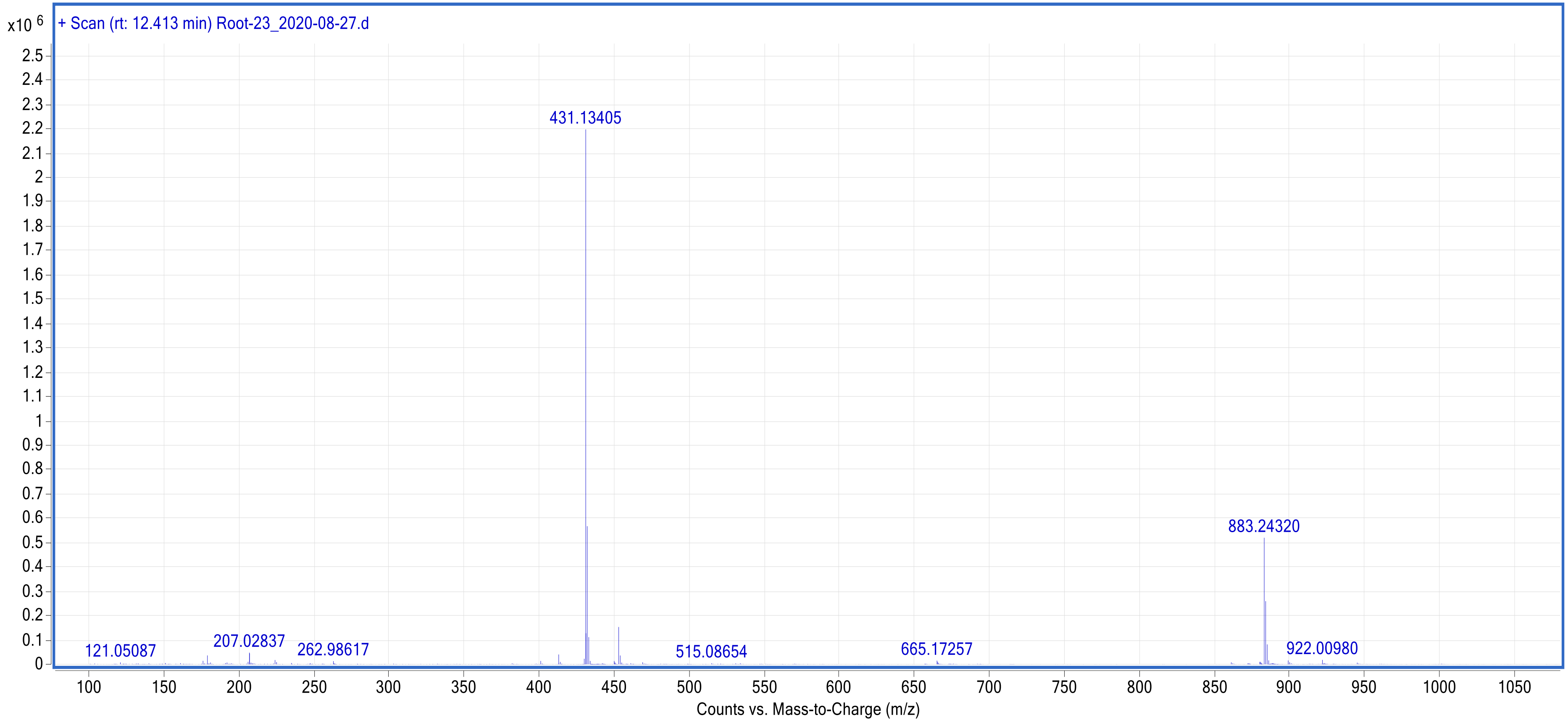

Figure S27. HRESI-MS spectrum of the new compound **3**

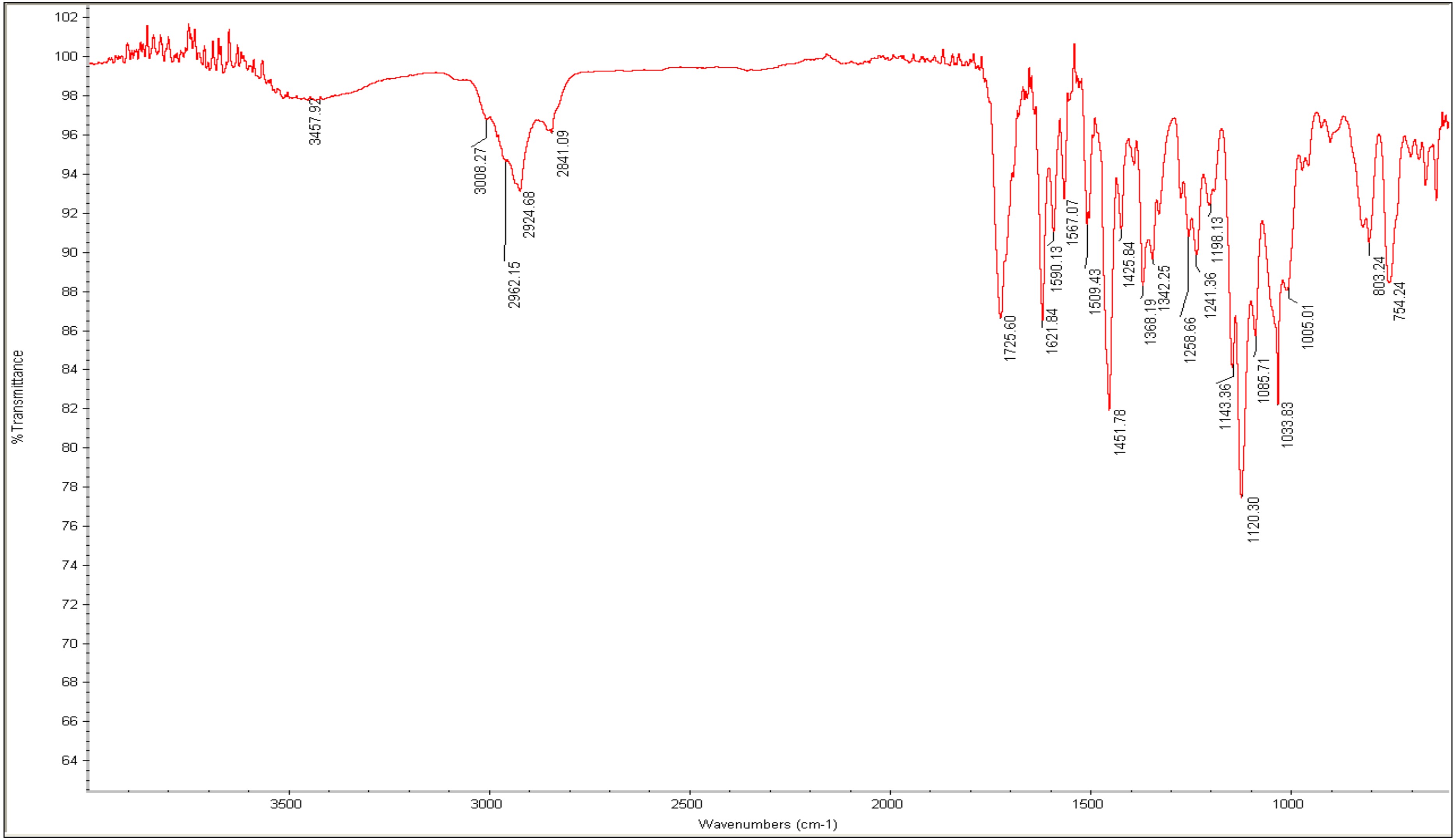

Figure S28. IR spectrum of the new compound **3**

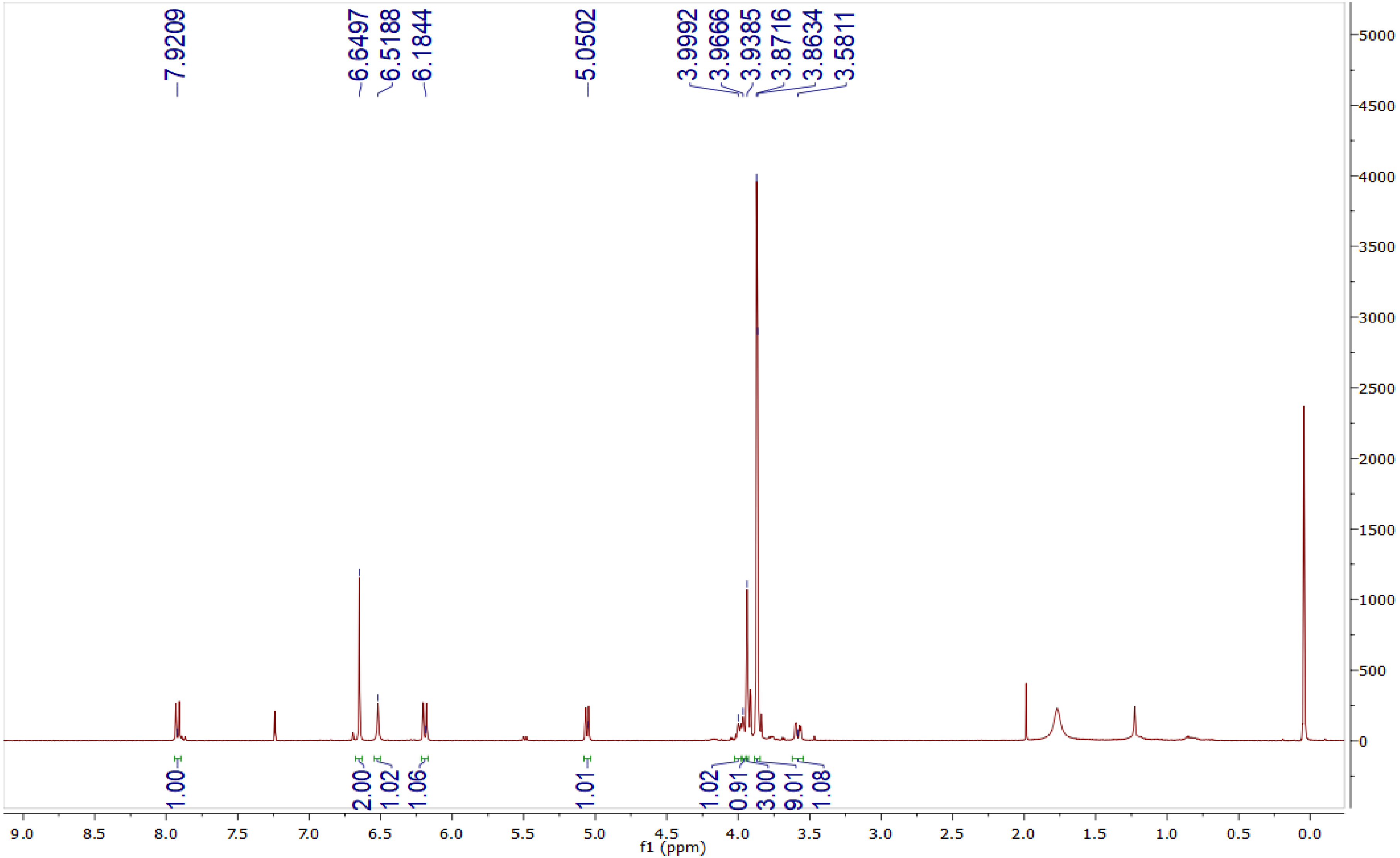

Figure S29. ^{1}H NMR (400 MHz, $CDCl_3$) spectrum of the new compound **3**

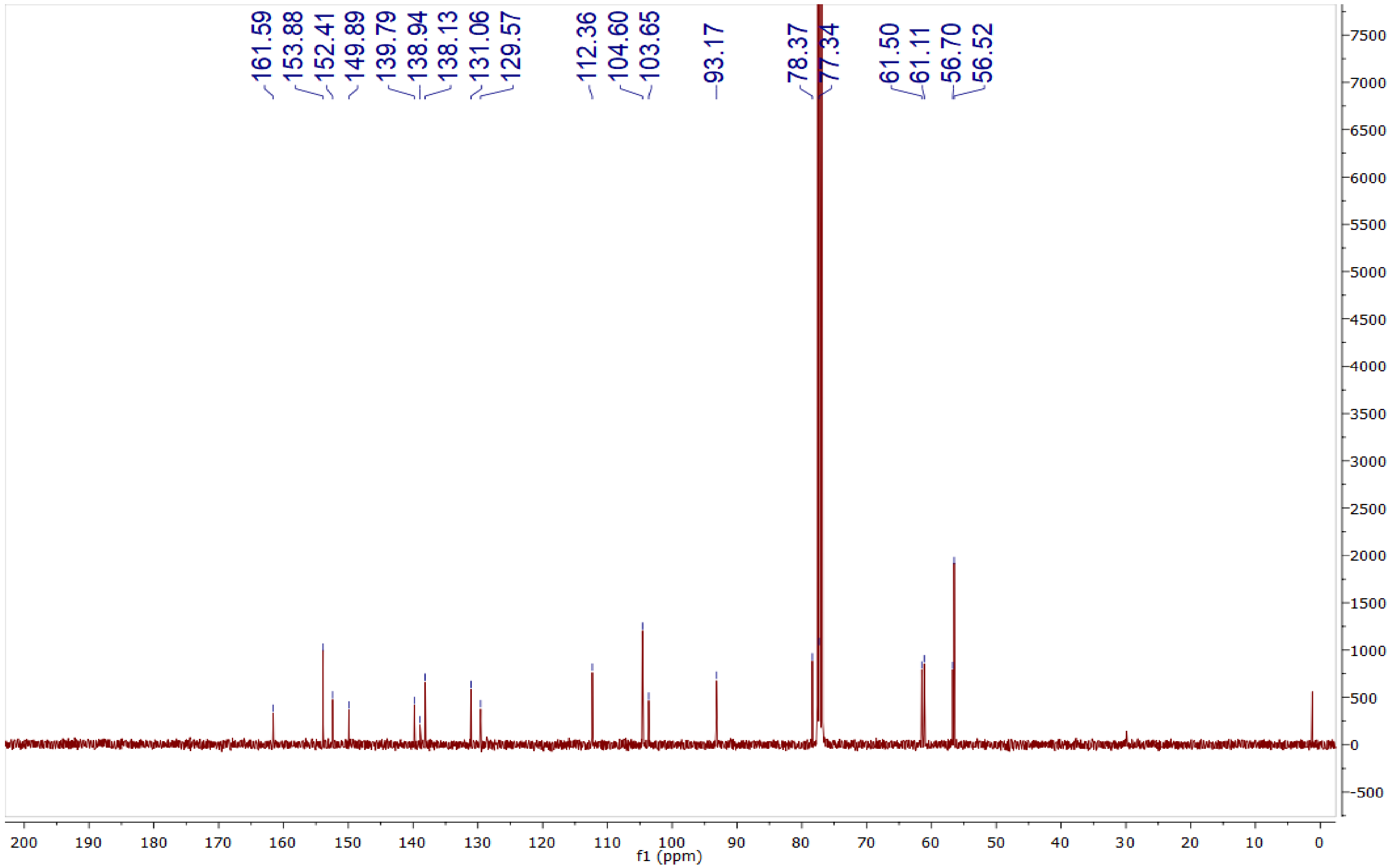

Figure S30. ^{13}C NMR (100 MHz, $CDCl_3$) spectrum of the new compound **3**

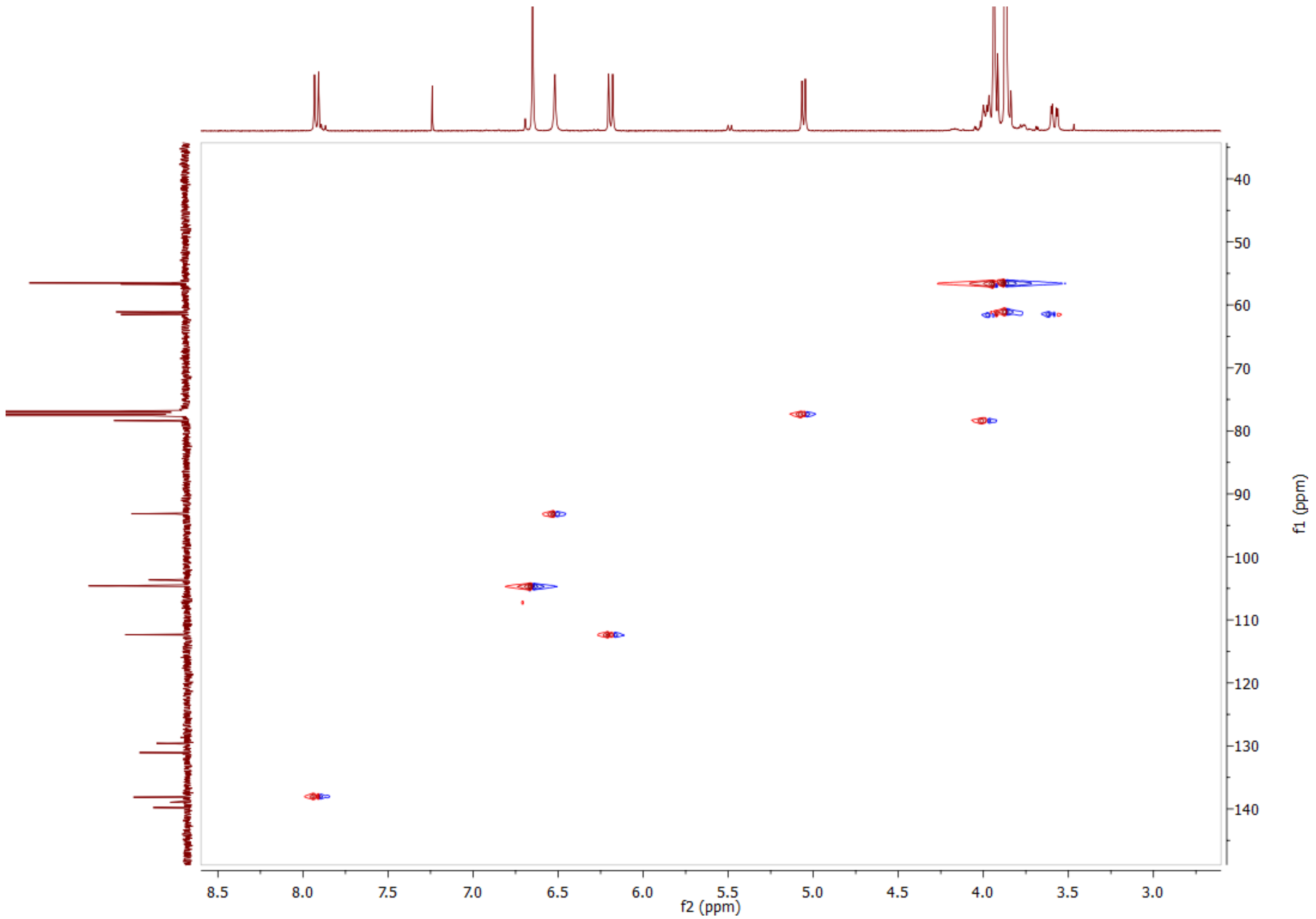

Figure S31. HSQC spectrum of the new compound **3**

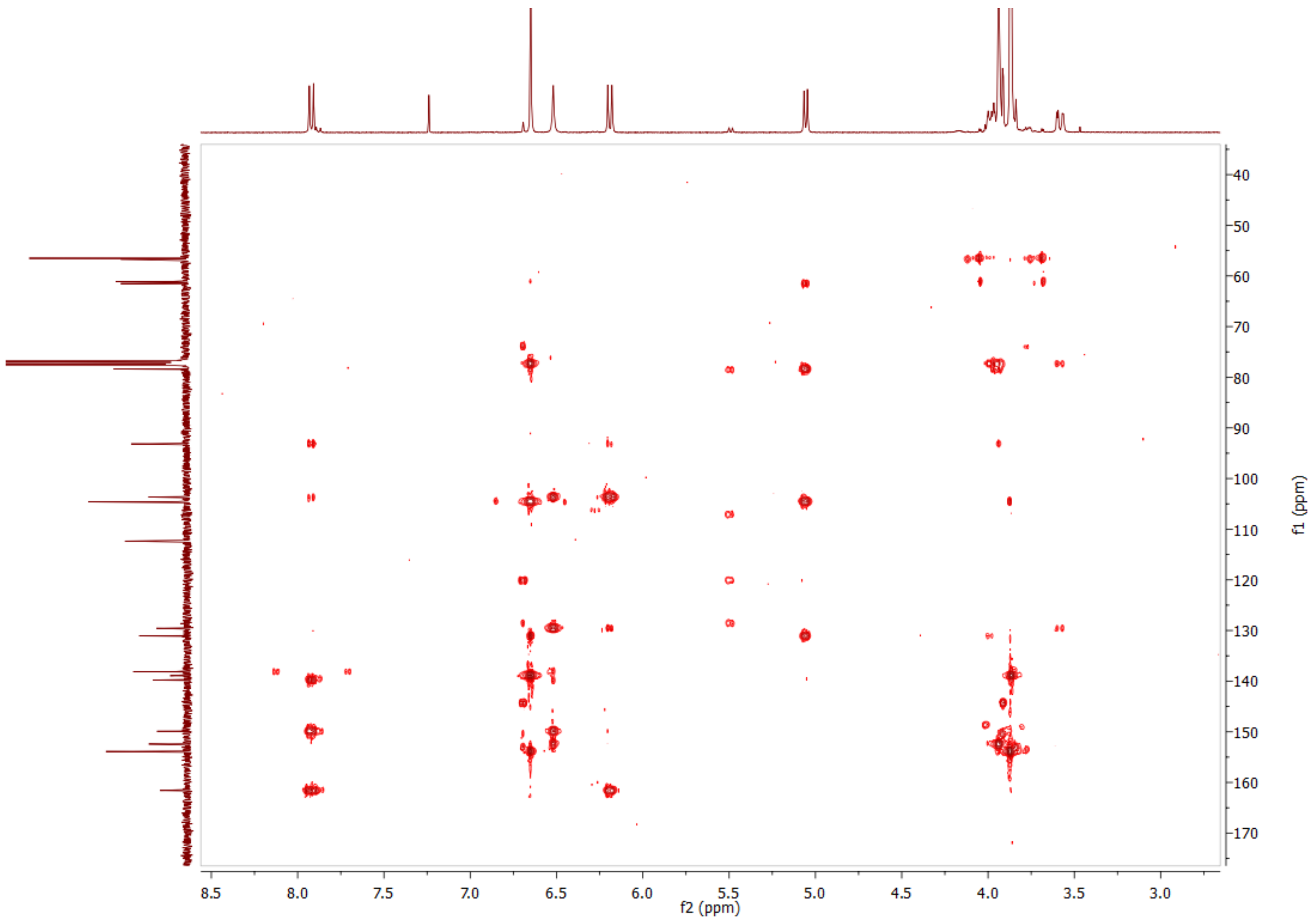

Figure S32. HMBC spectrum of the new compound **3**

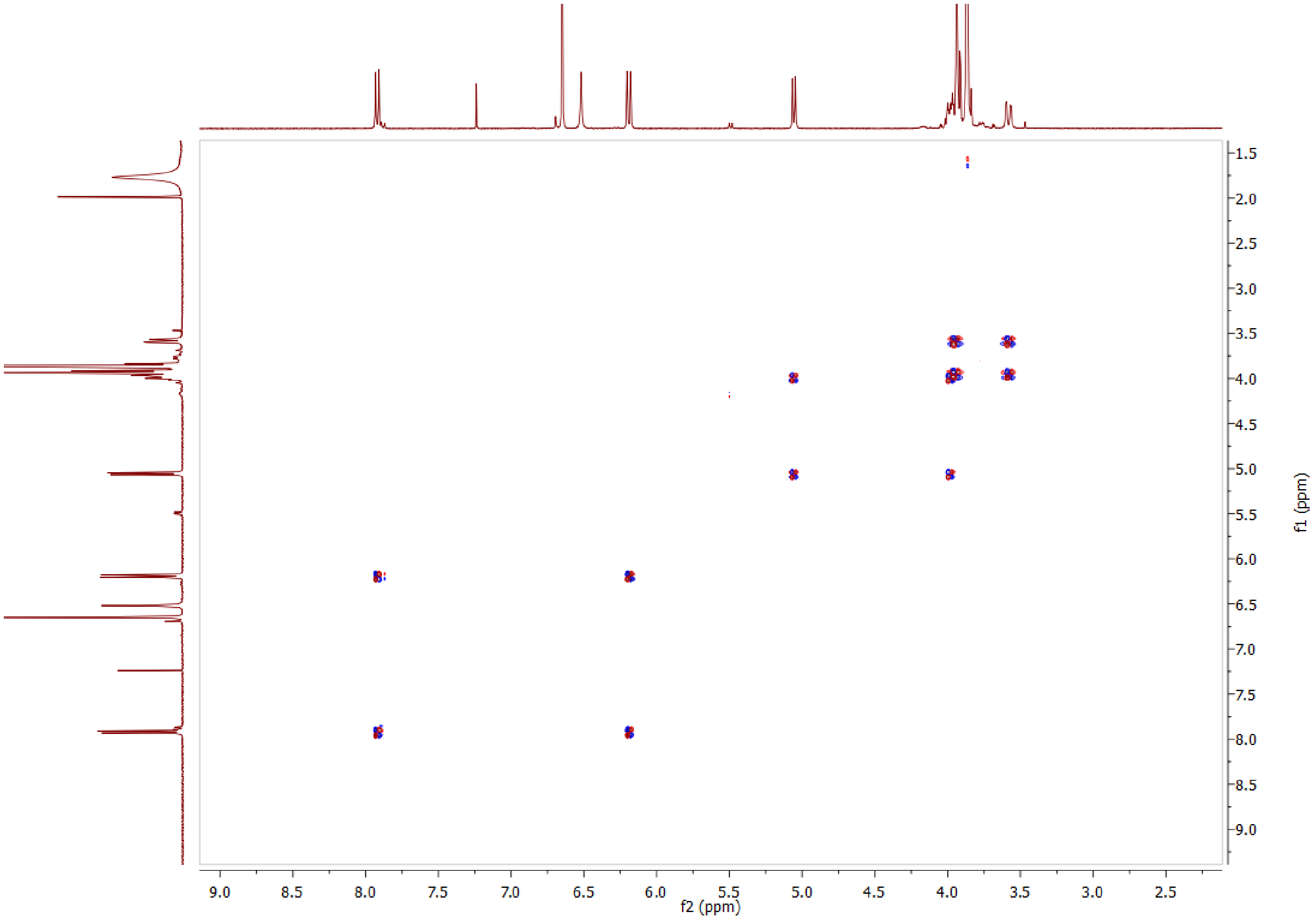

Figure S33. COSY spectrum of the new compound **3**

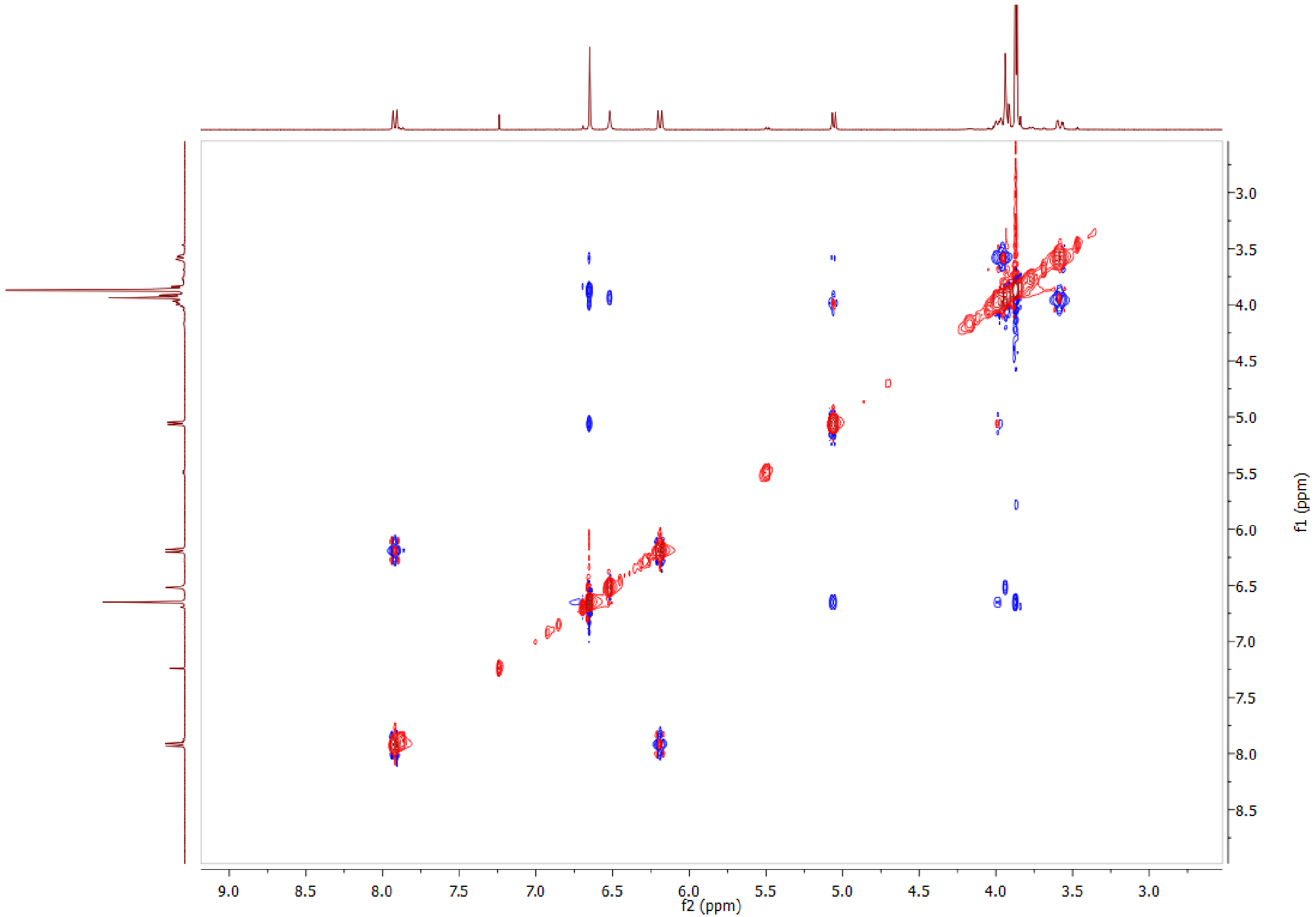

Figure S34. NOESY spectrum of the new compound **3**

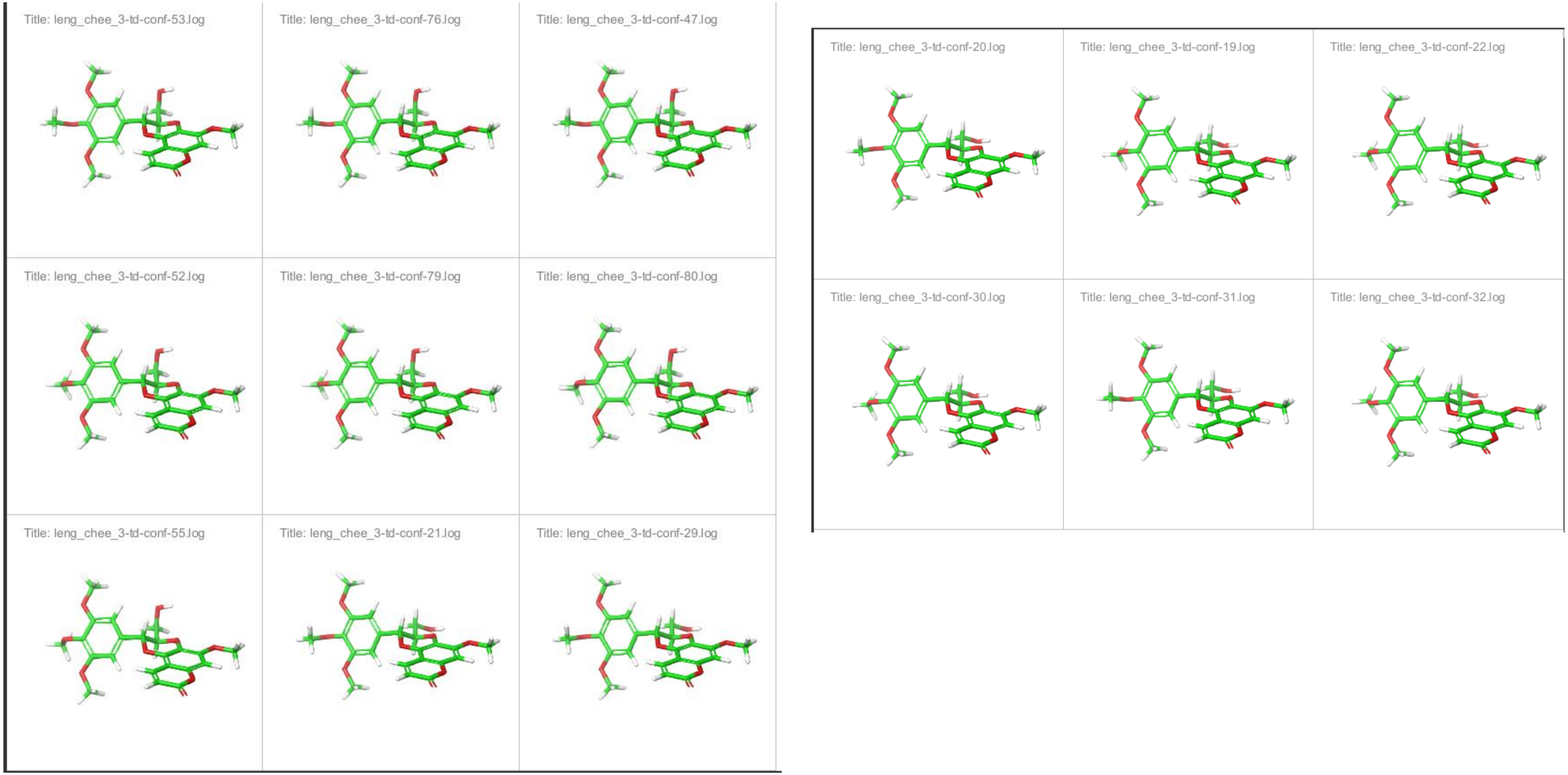

Figure S35. Images of Conformers (>1%) for the new compound **3**

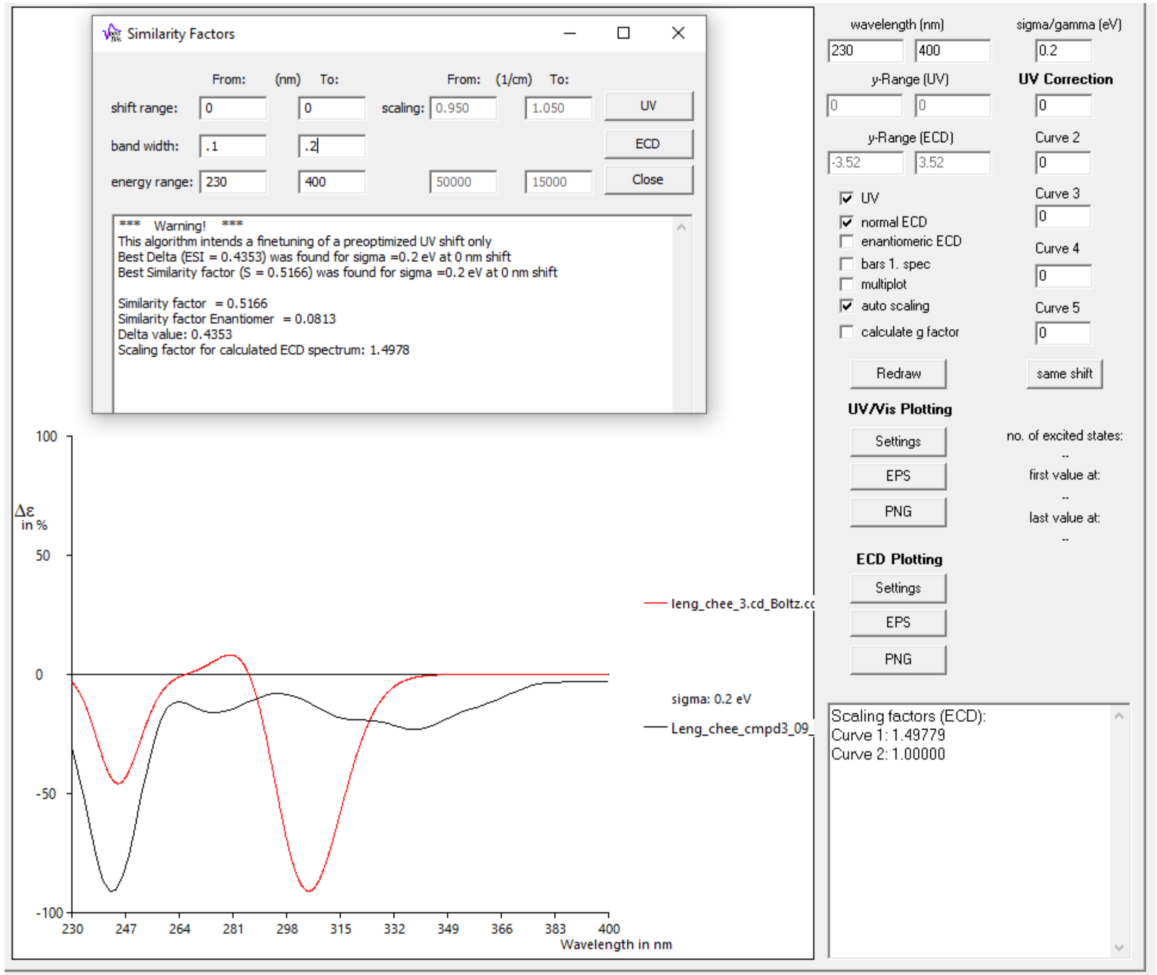

Figure S36. CD spectrum (experimental to calculated comparison by SpecDis) of the new compound **3**

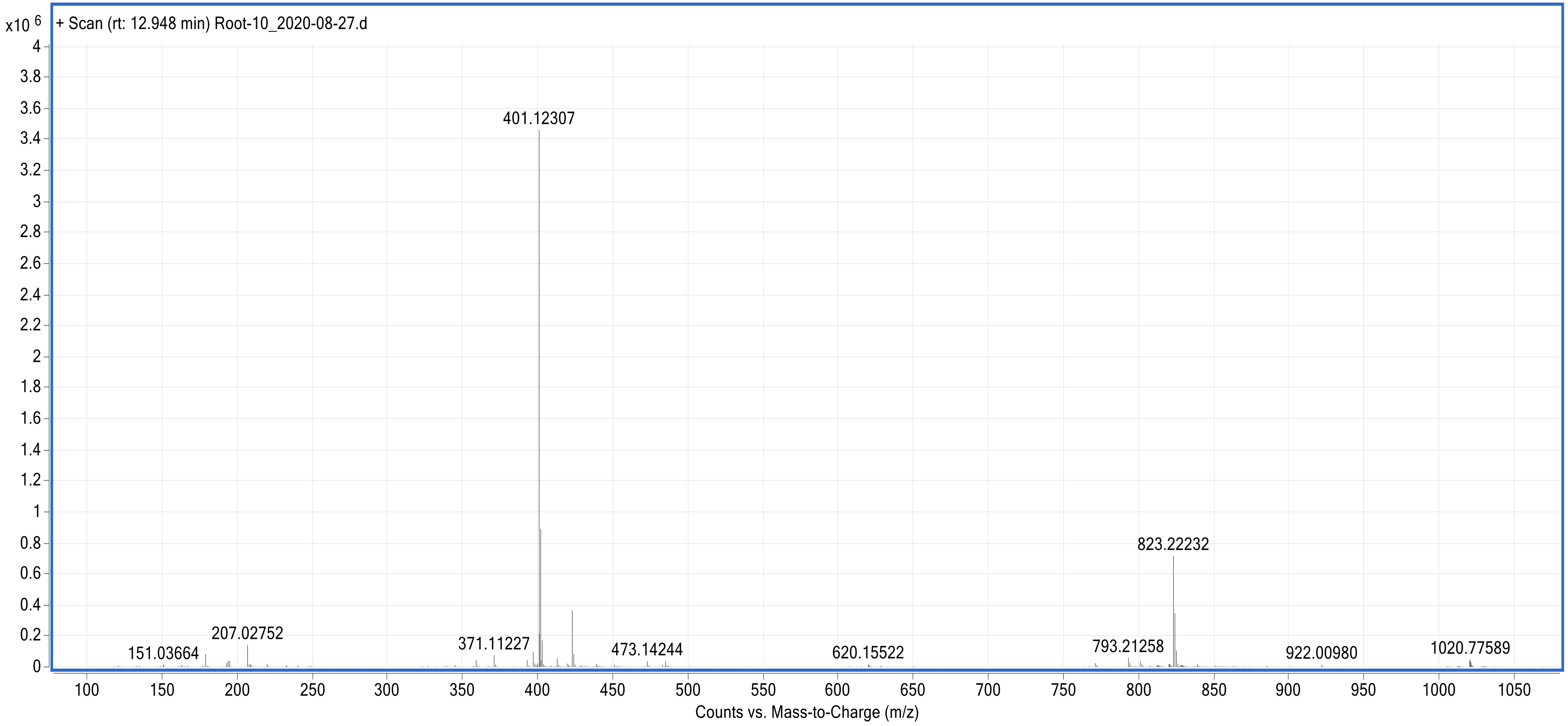

Figure S37. HRESI-MS spectrum of the new compound **4**

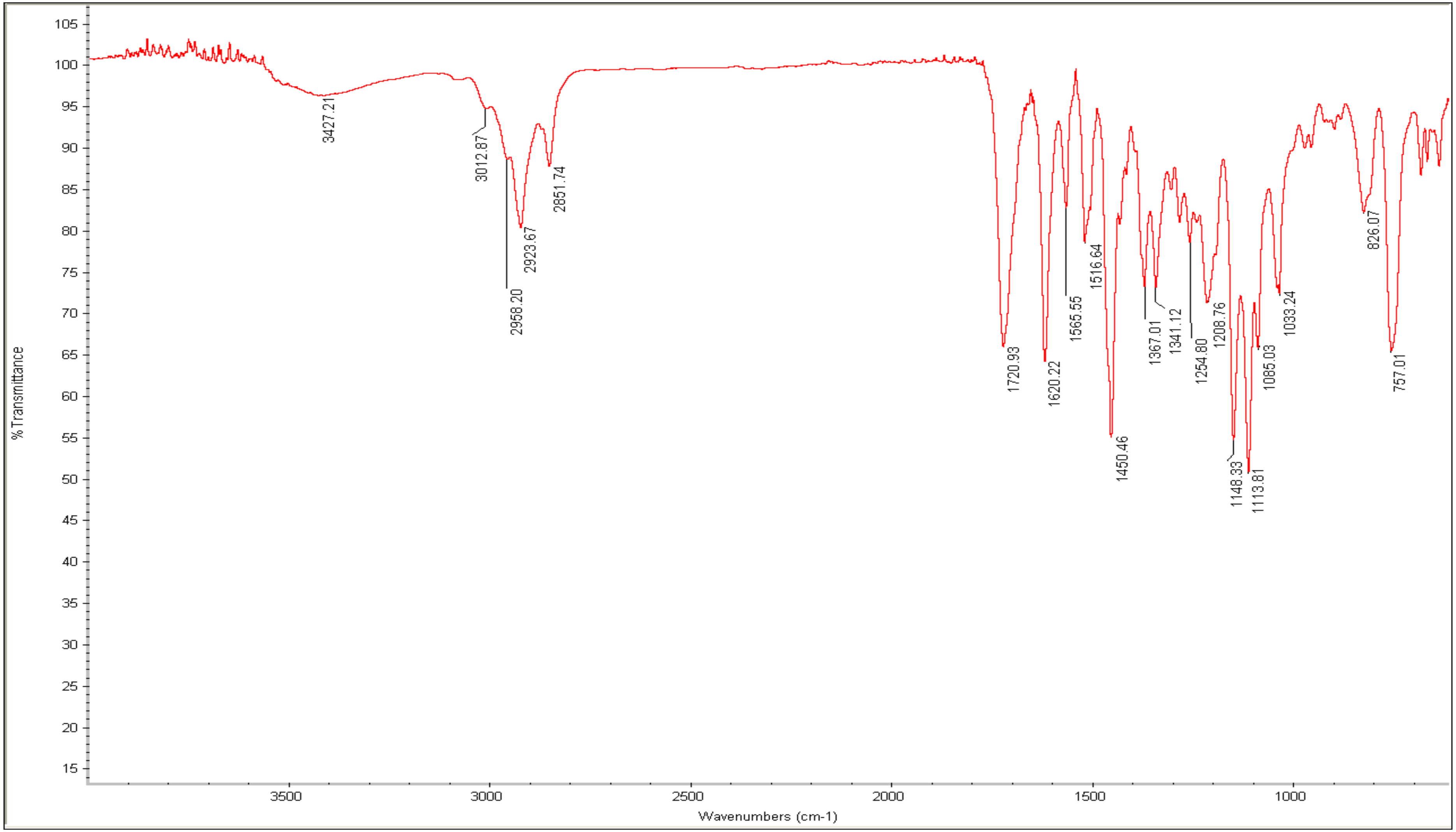

Figure S38. IR spectrum of the new compound **4**

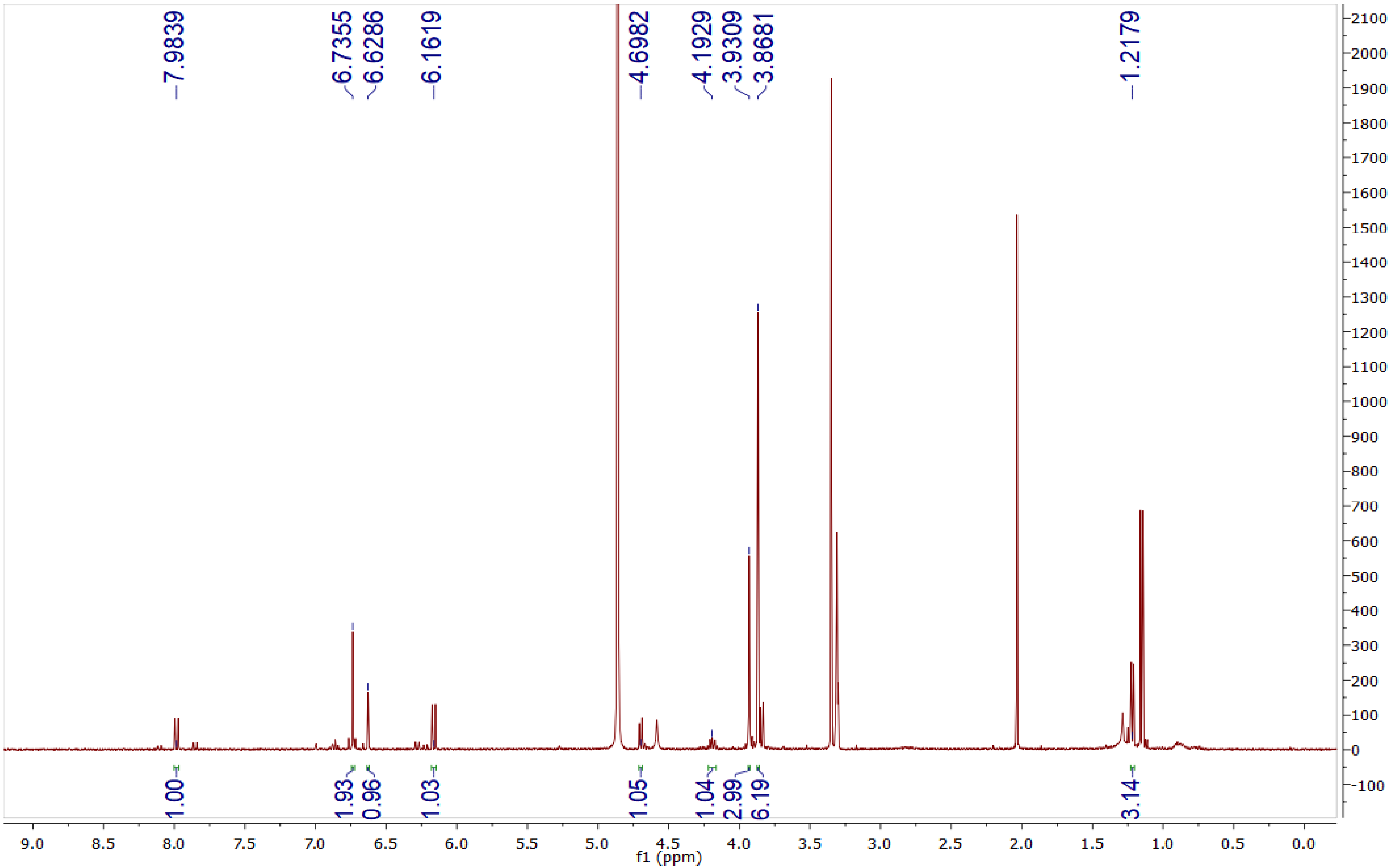

Figure S39. ^{1}H NMR (400 MHz, MeOD) spectrum of the new compound **4**

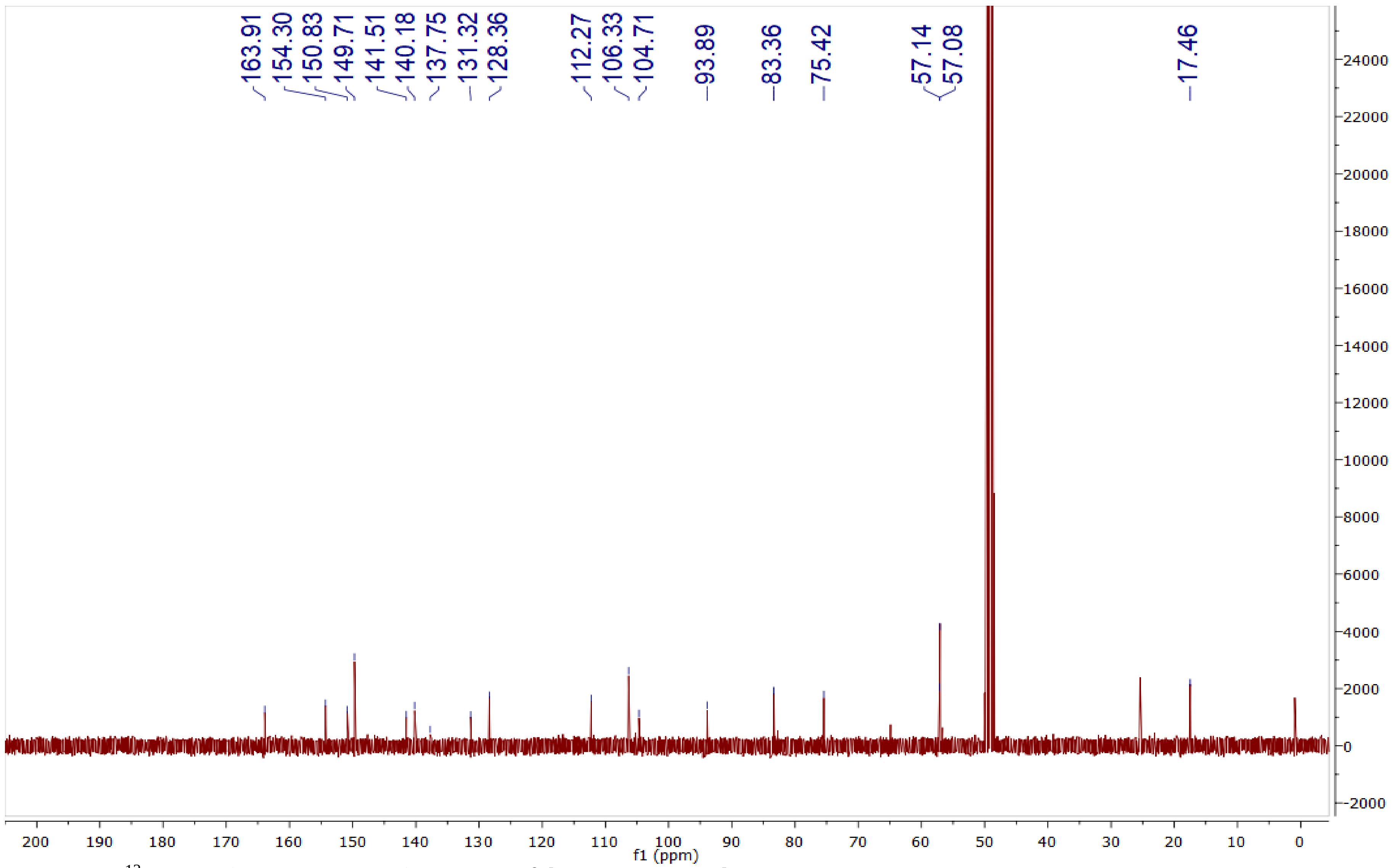

Figure S40. ^{13}C NMR (100 MHz, MeOD) spectrum of the new compound **4**

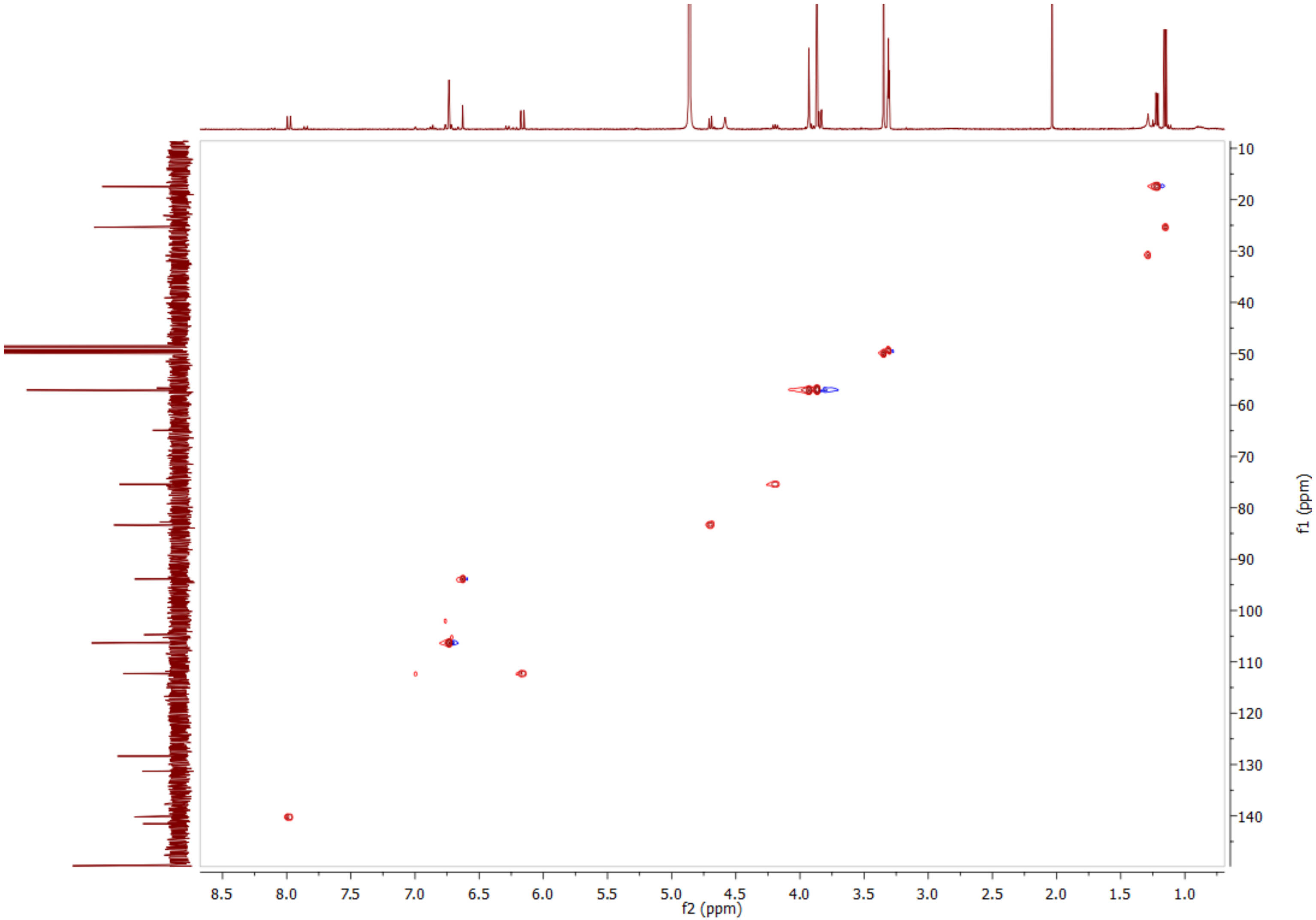

Figure S41. HSQC spectrum of the new compound **4**

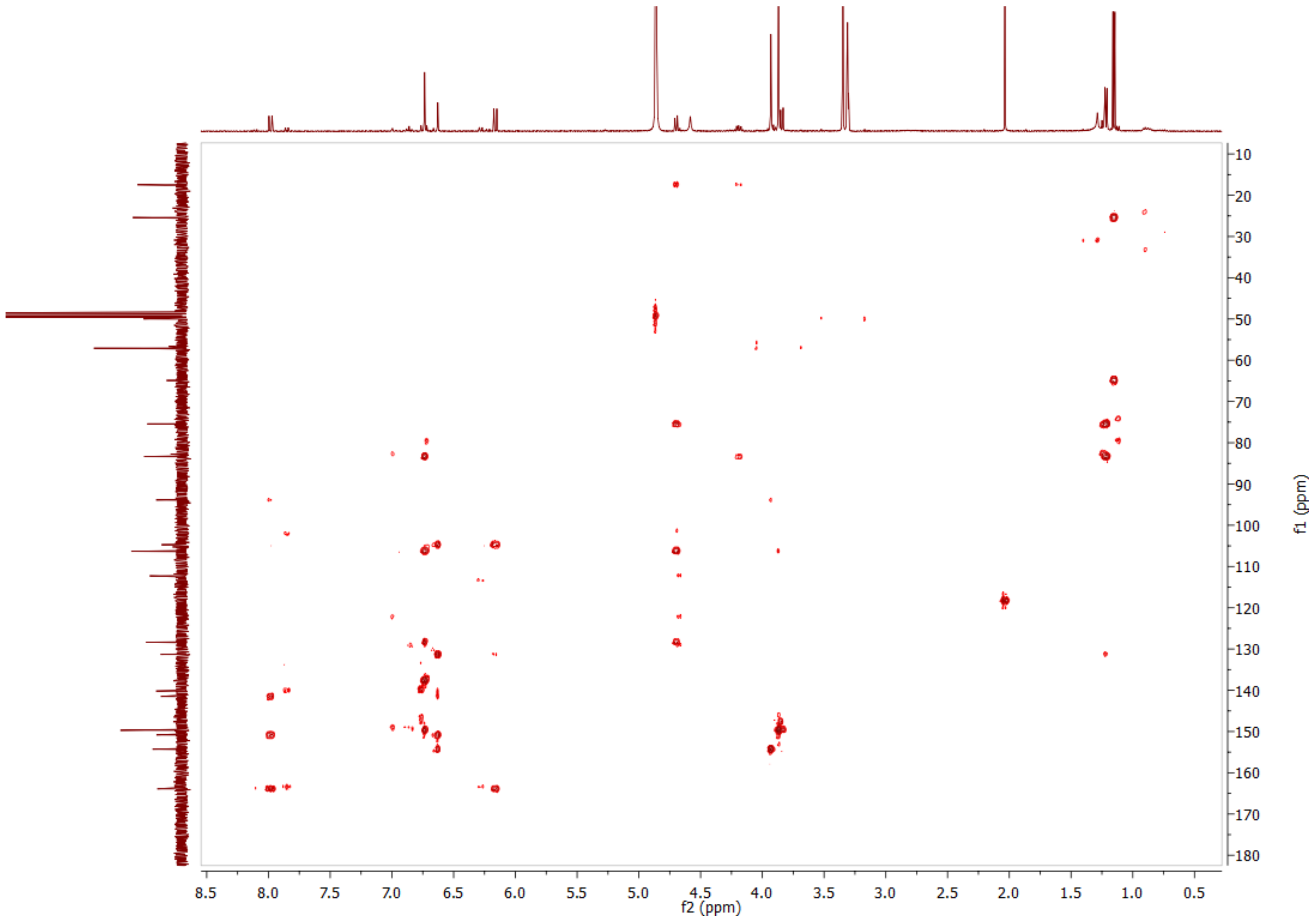

Figure S42. HMBC spectrum of the new compound **4**

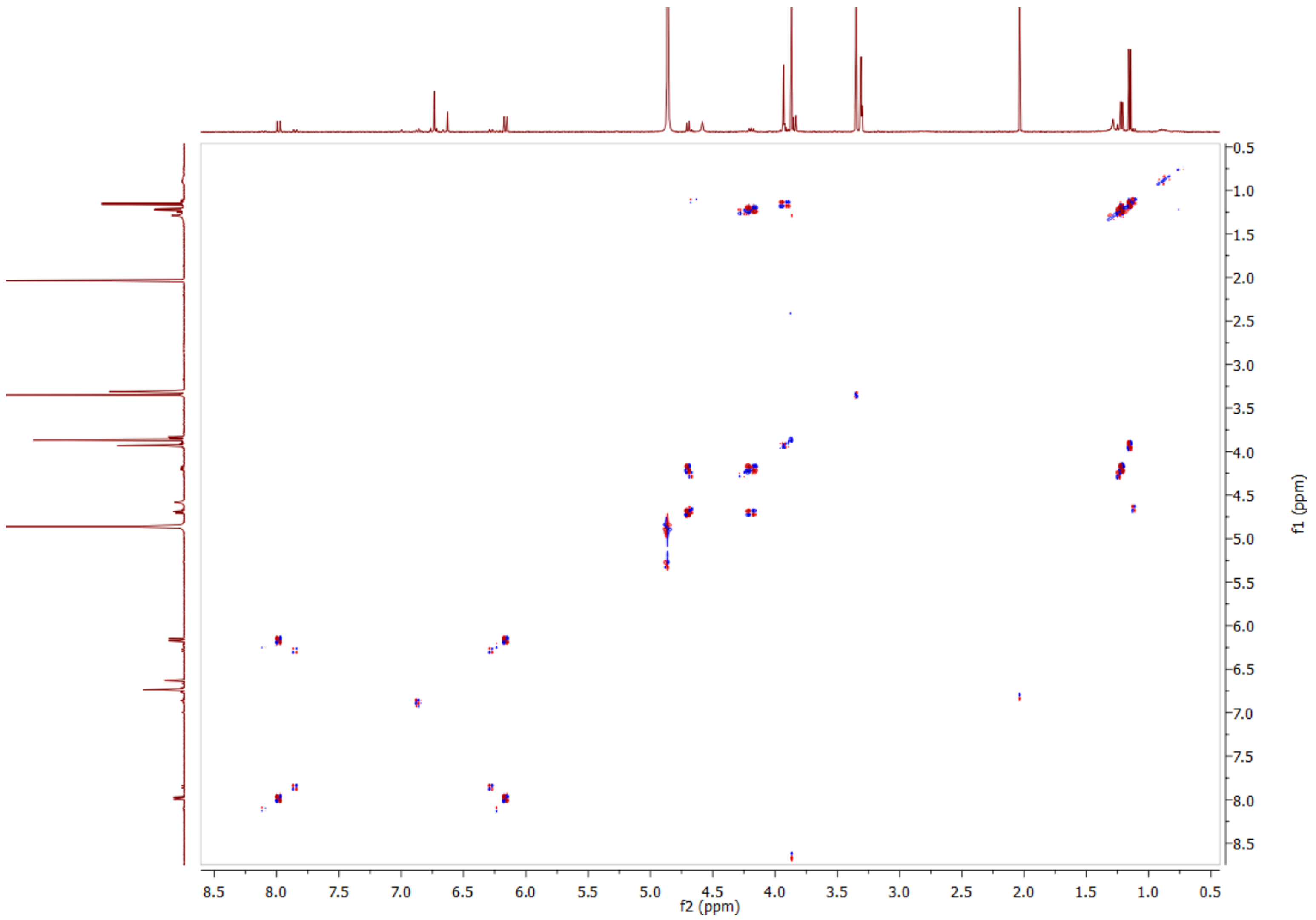

Figure S43. COSY spectrum of the new compound **4**

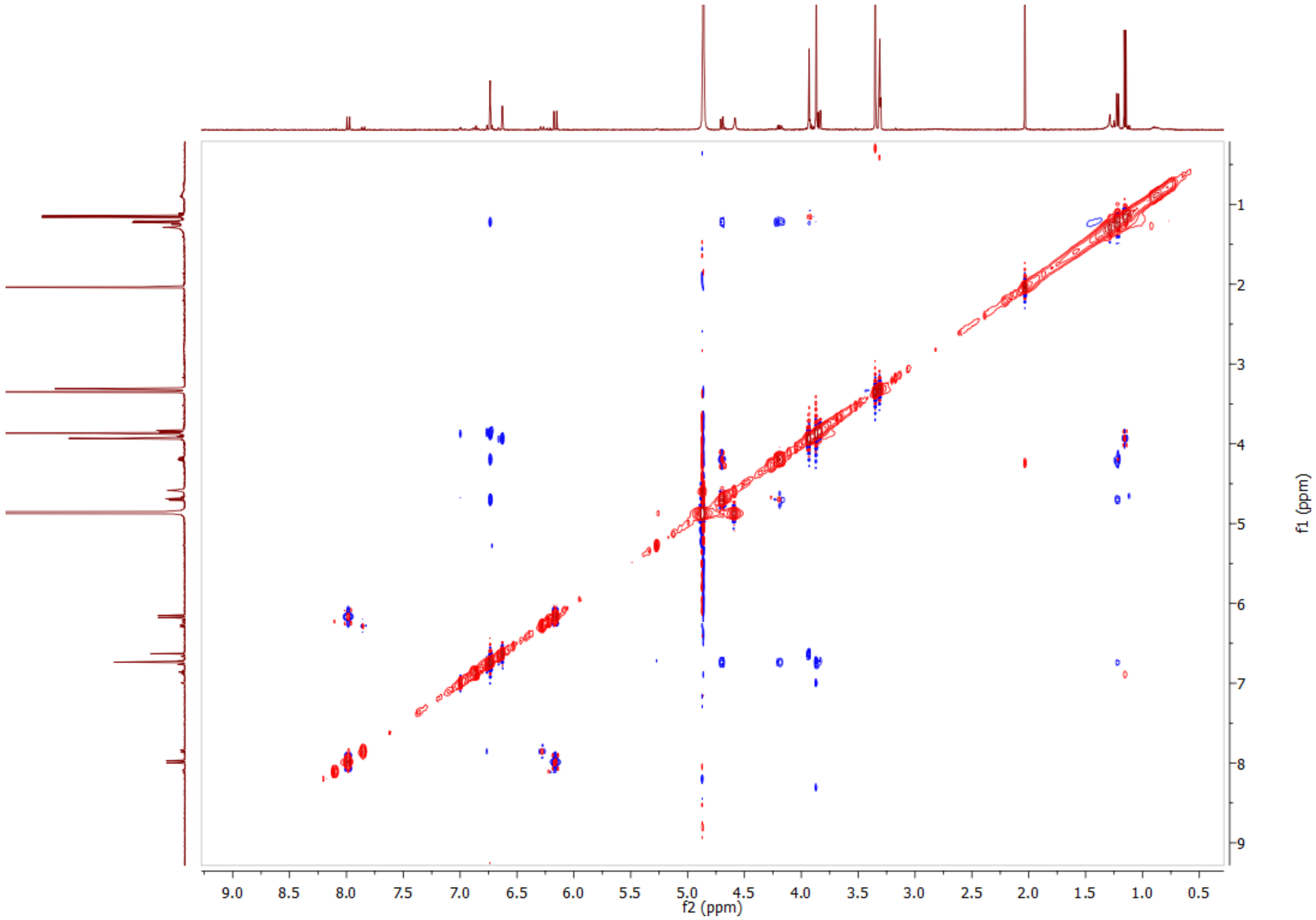

Figure S44. NOESY spectrum of the new compound **4**

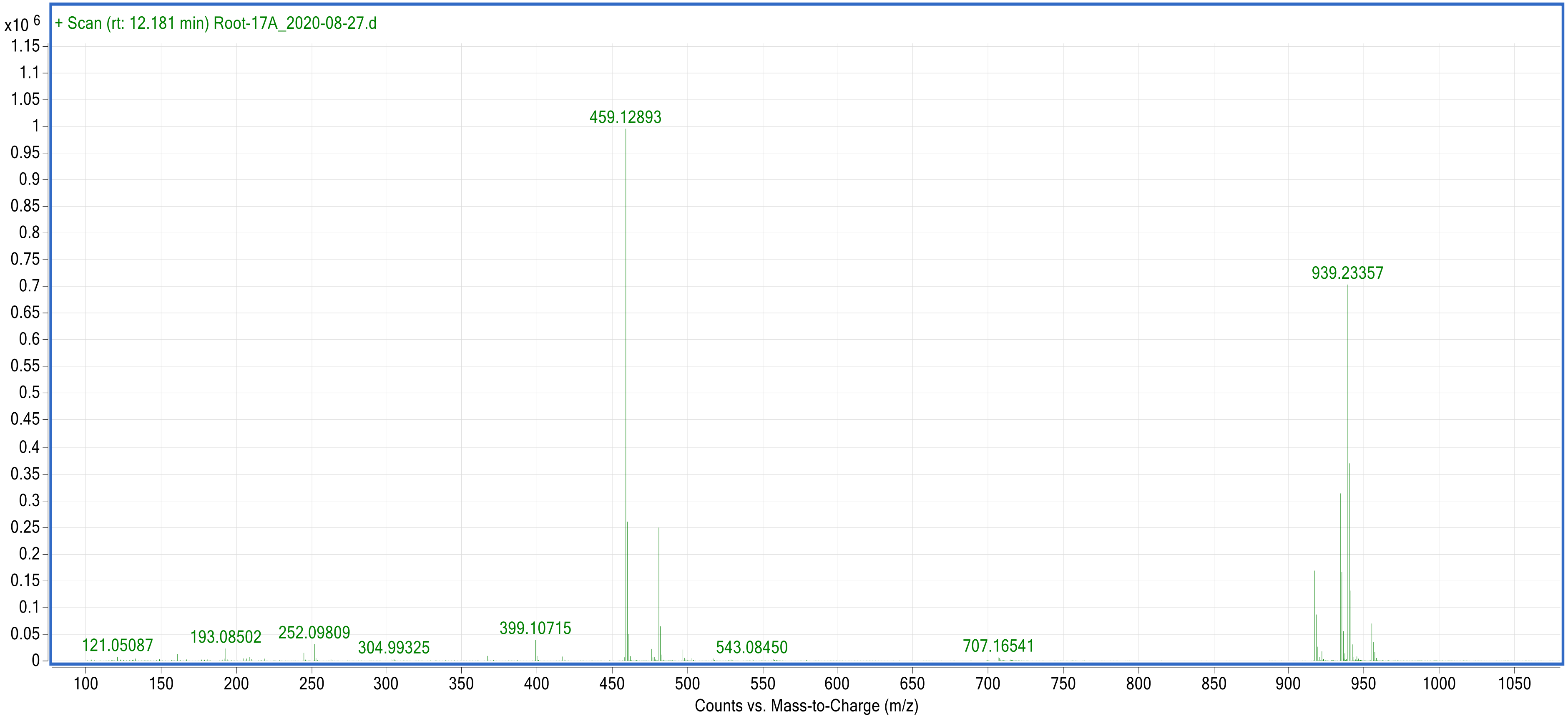

Figure S45. HRESI-MS spectrum of the new compound **5**

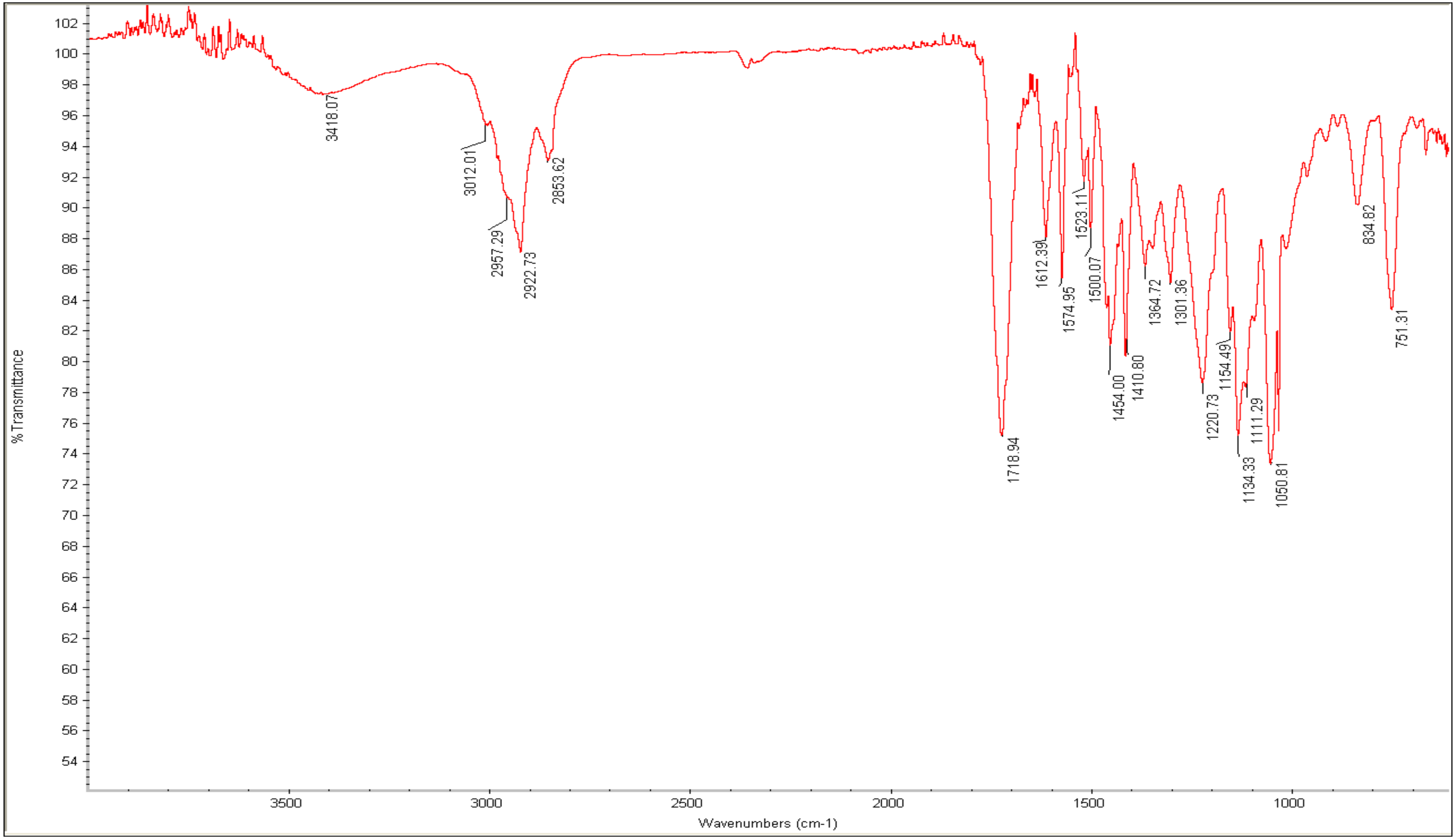

Figure S46. IR spectrum of the new compound **5**

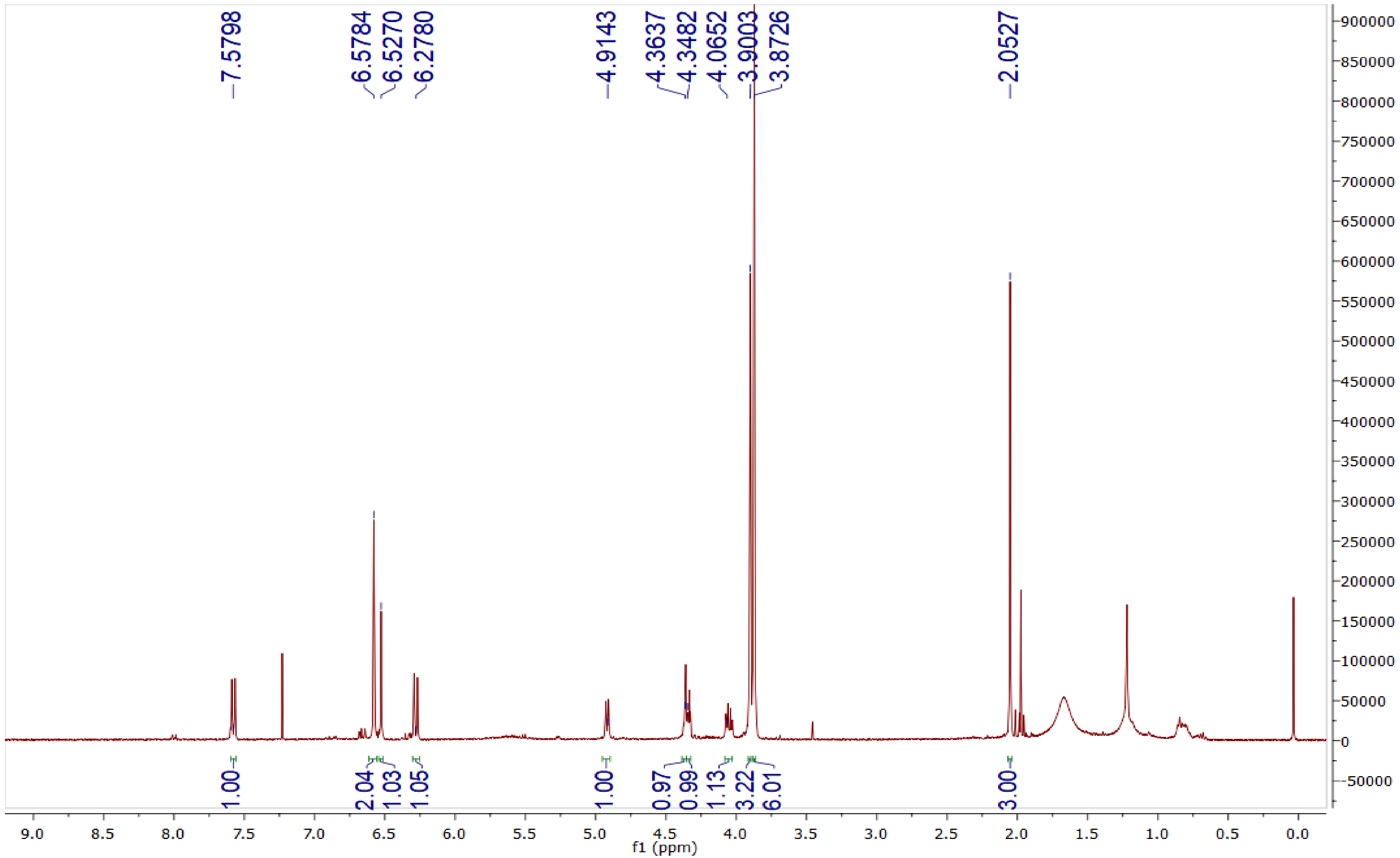

Figure S47. ^{1}H NMR (400 MHz, $CDCl_3$) spectrum of the new compound **5**

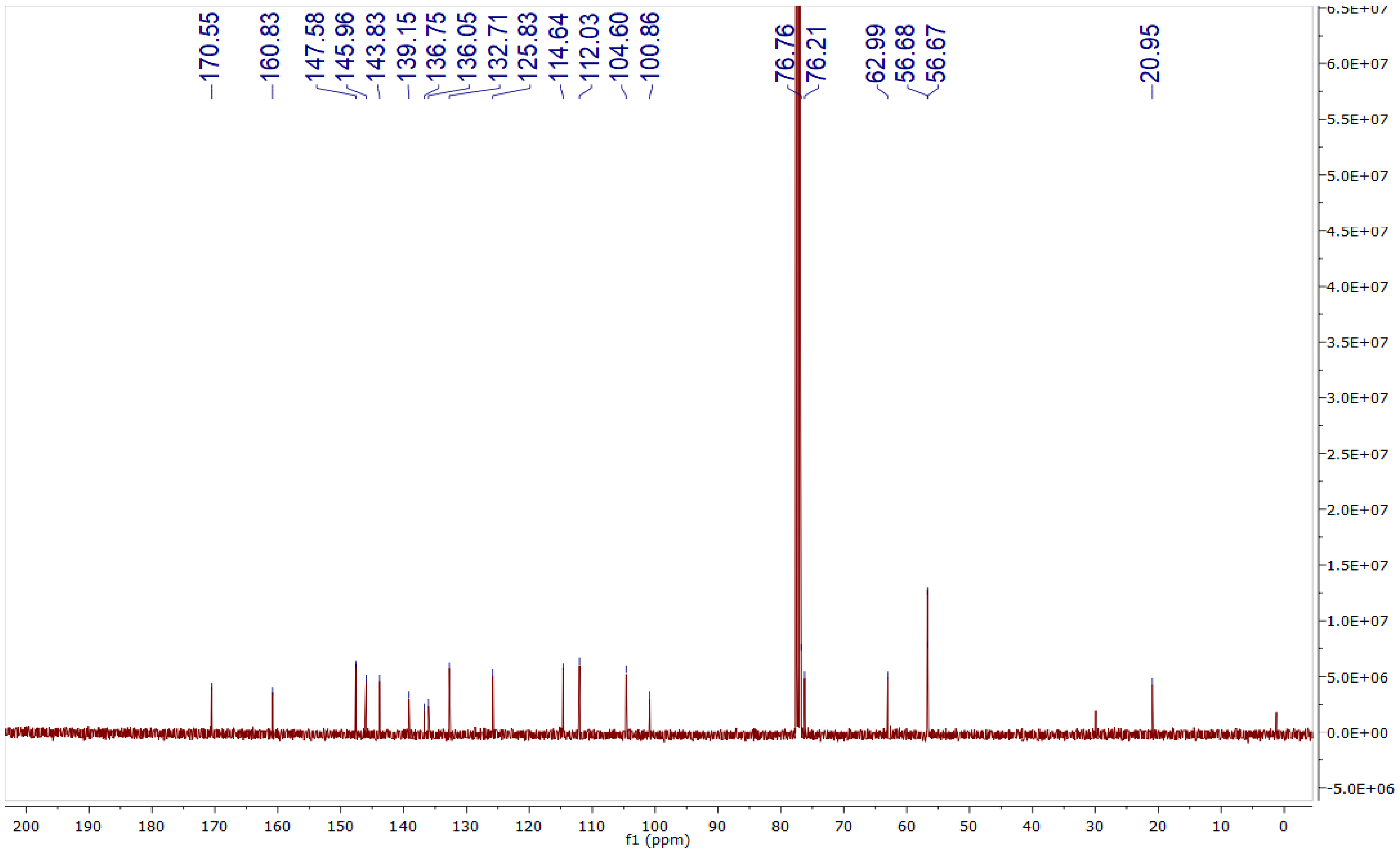

Figure S48. ^{13}C NMR (100 MHz, $CDCl_3$) spectrum of the new compound **5**

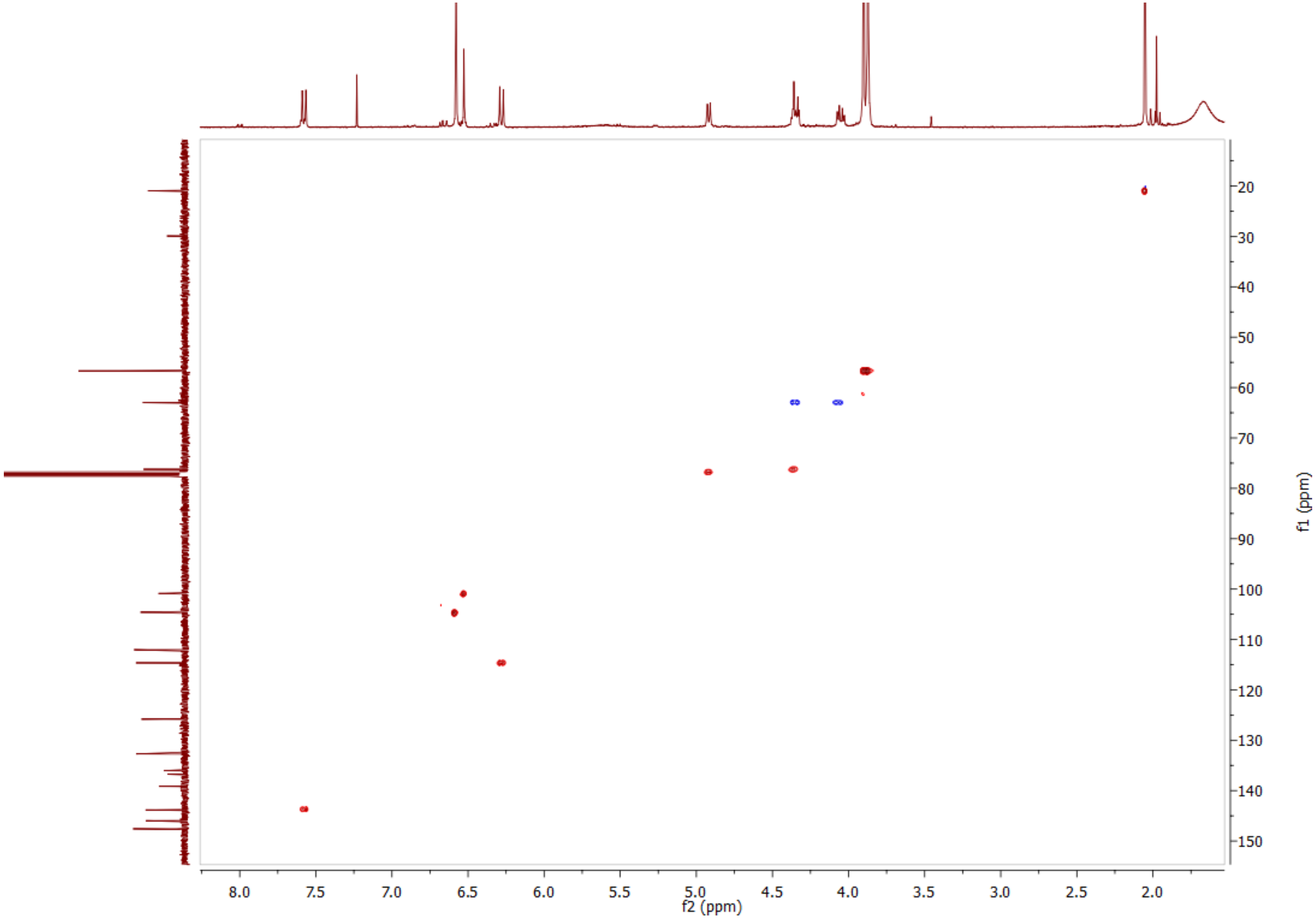

Figure S49. HSQC spectrum of the new compound **5**

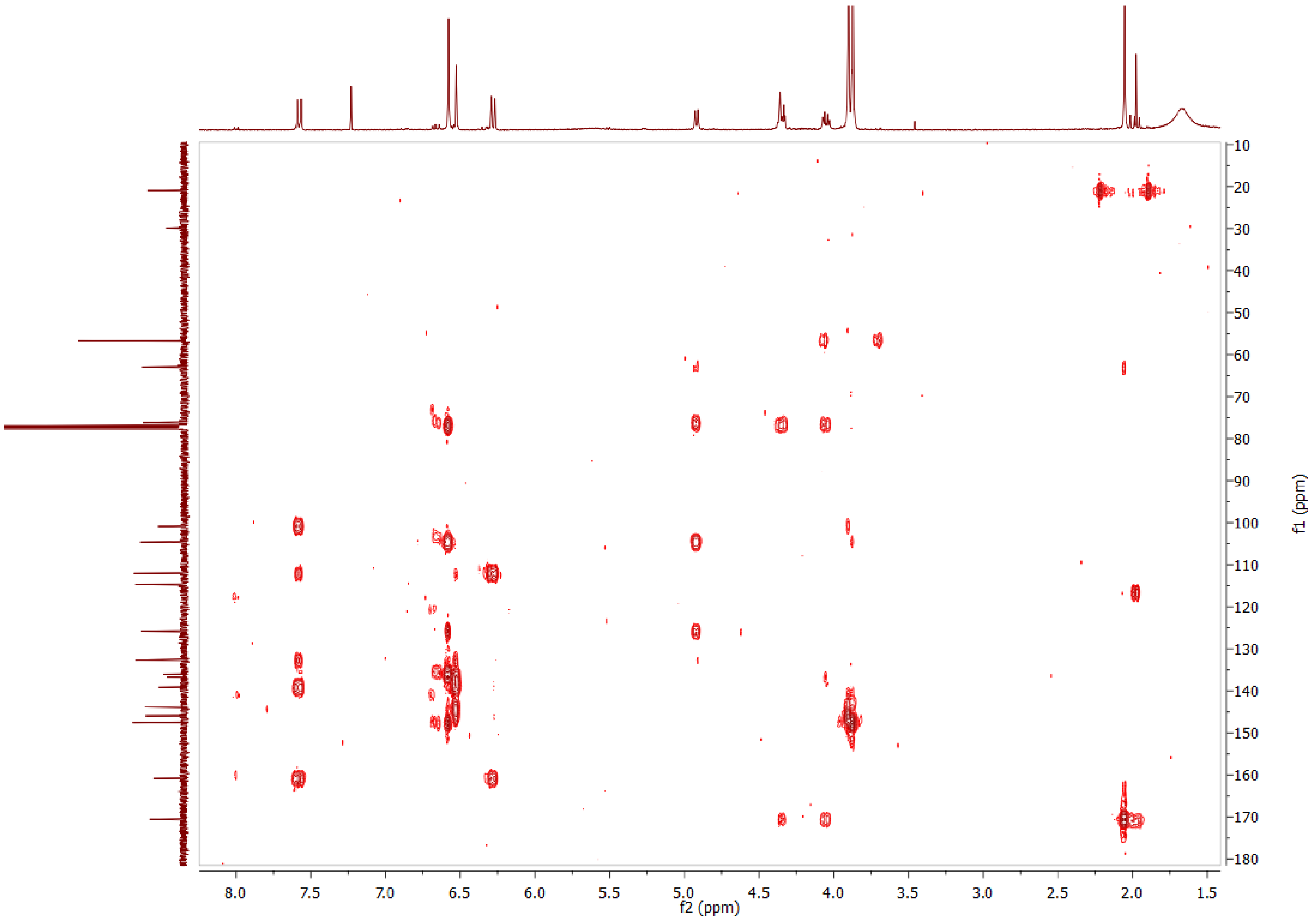

Figure S50. HMBC spectrum of the new compound **5**

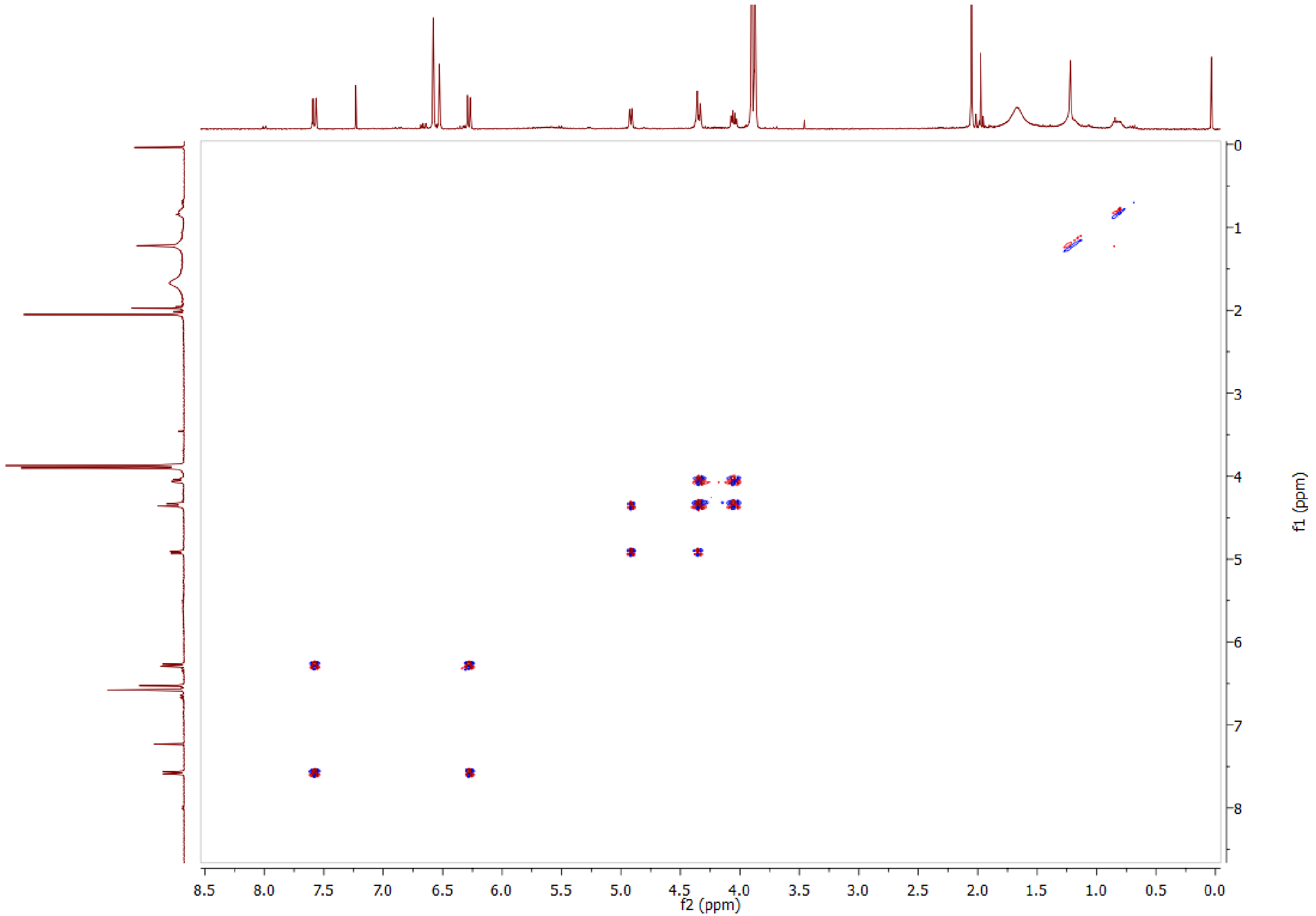

Figure S51. COSY spectrum of the new compound **5**

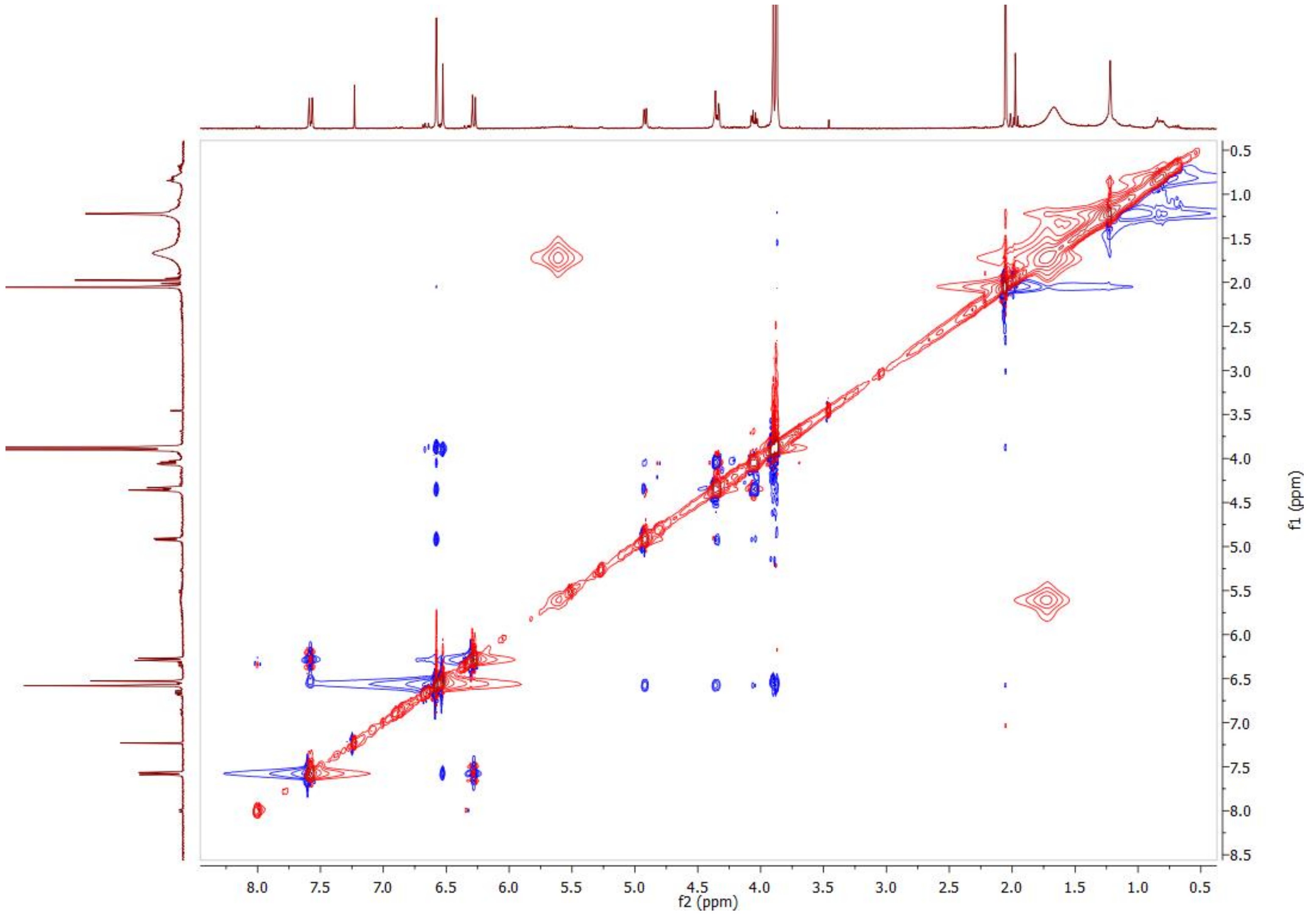

Figure S52. NOESY spectrum of the new compound **5**

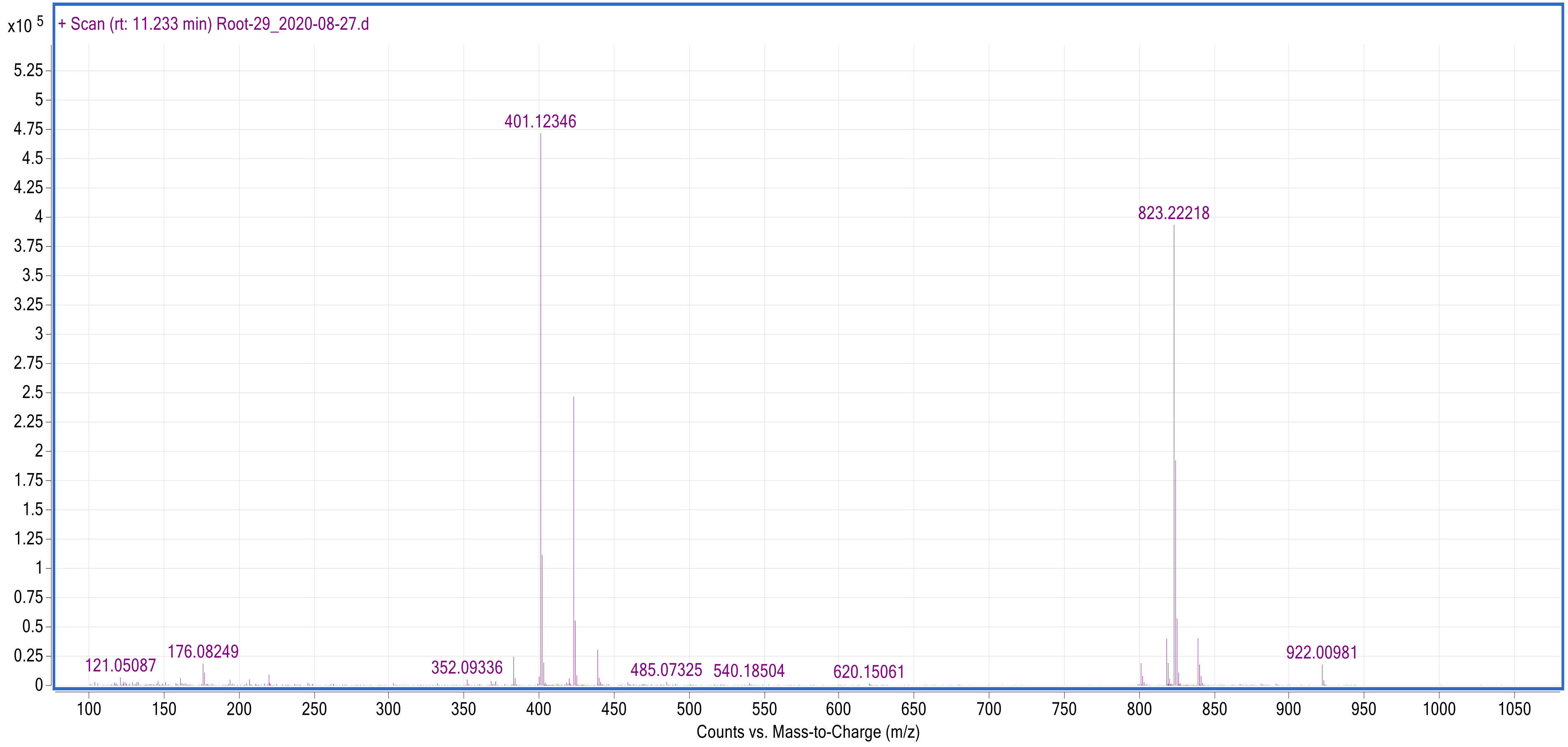

Figure S53. HRESI-MS spectrum of the new compound **7**

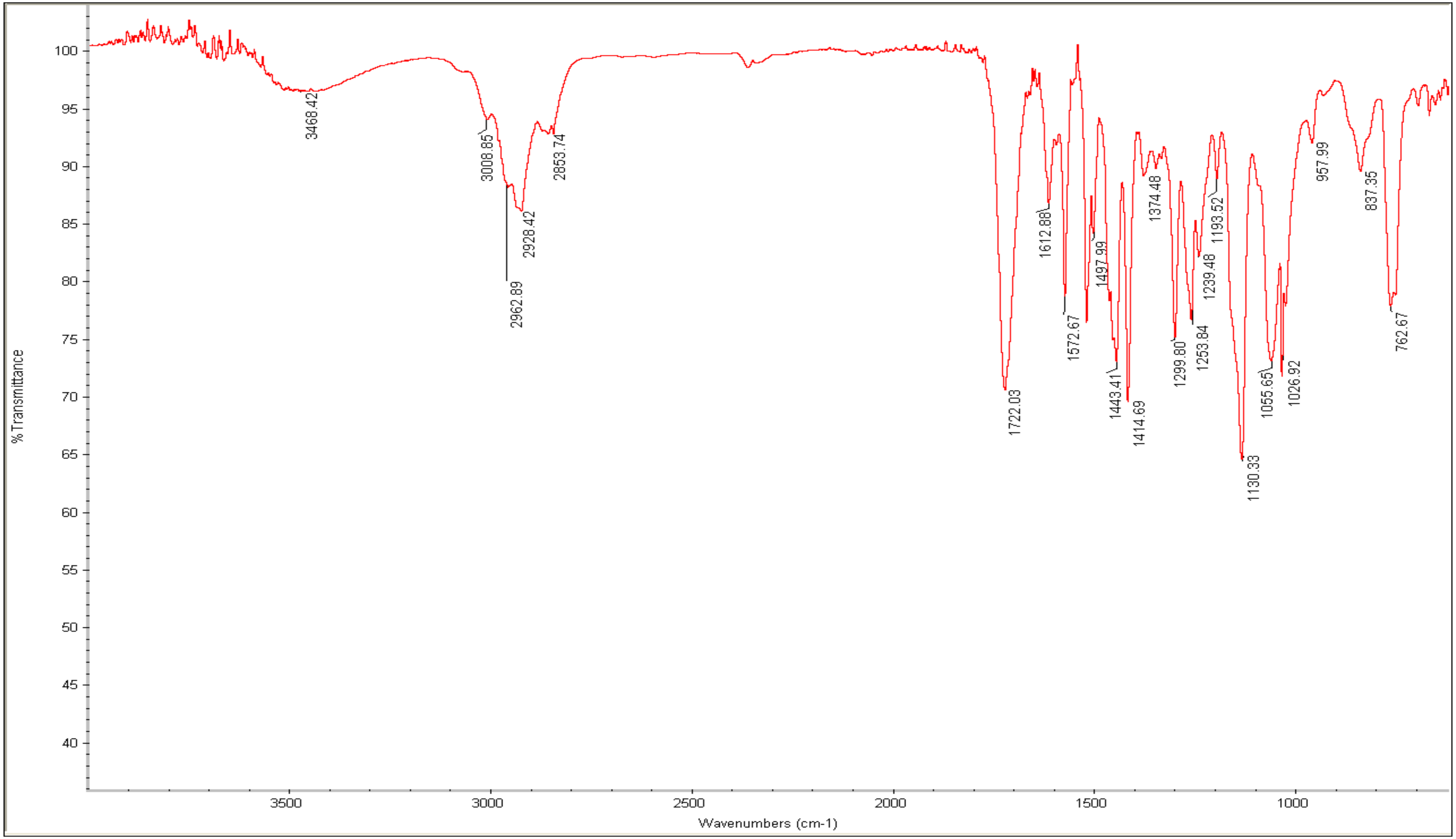

Figure S54. IR spectrum of the new compound **7**

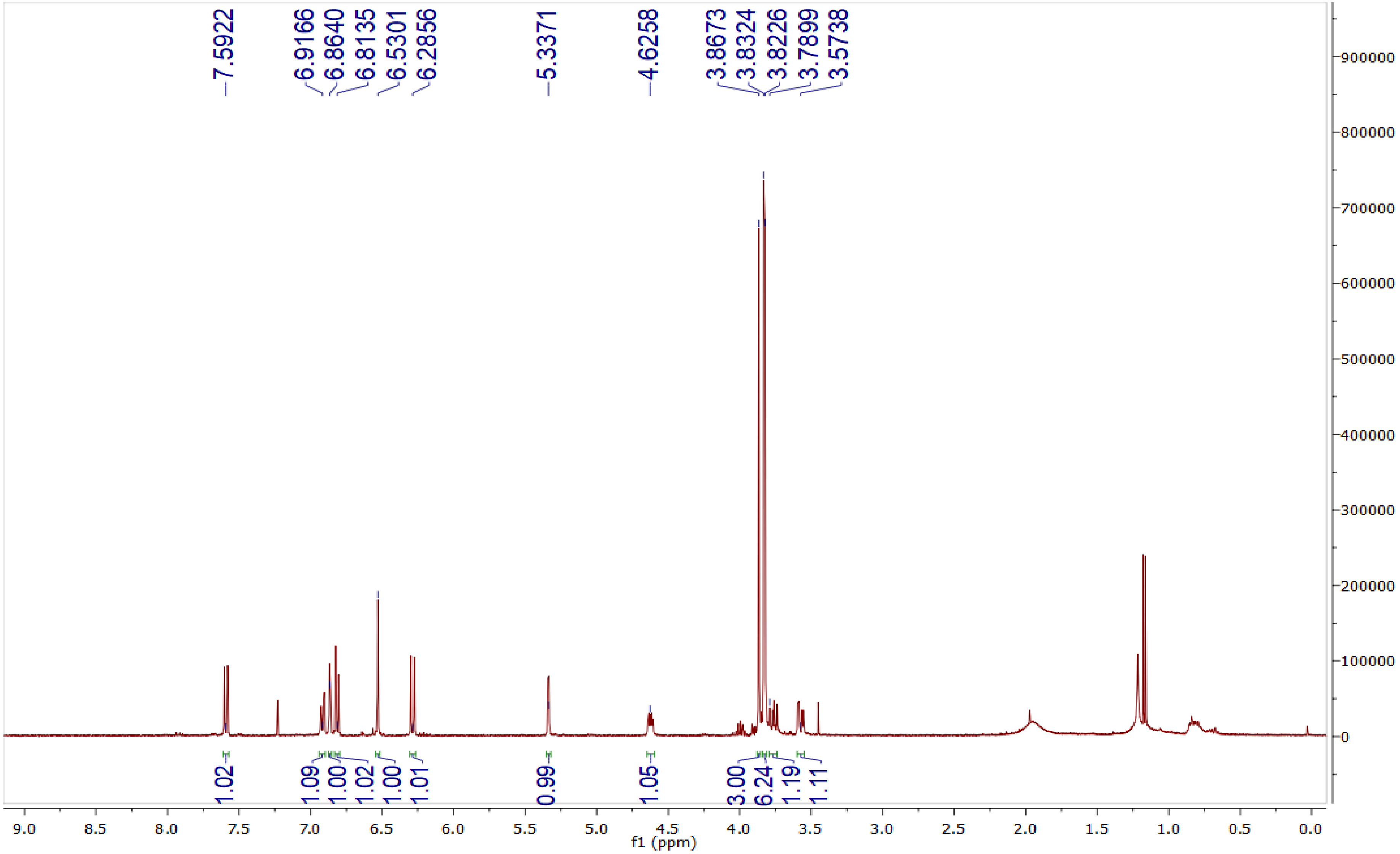

Figure S55. ^{1}H NMR (400 MHz, $CDCl_3$) spectrum of the new compound **7**

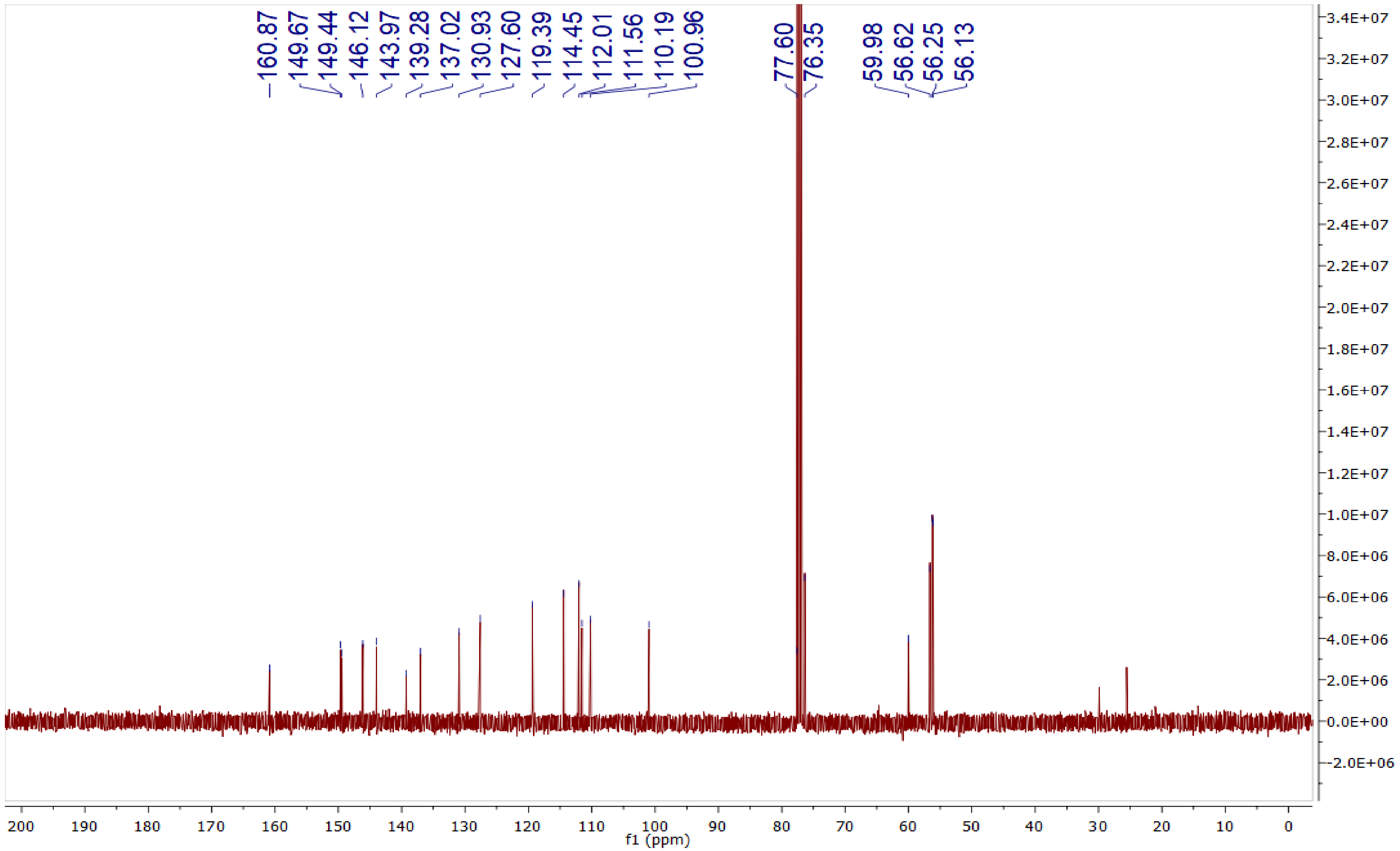

Figure S56. ^{13}C NMR (100 MHz, $CDCl_3$) spectrum of the new compound **7**

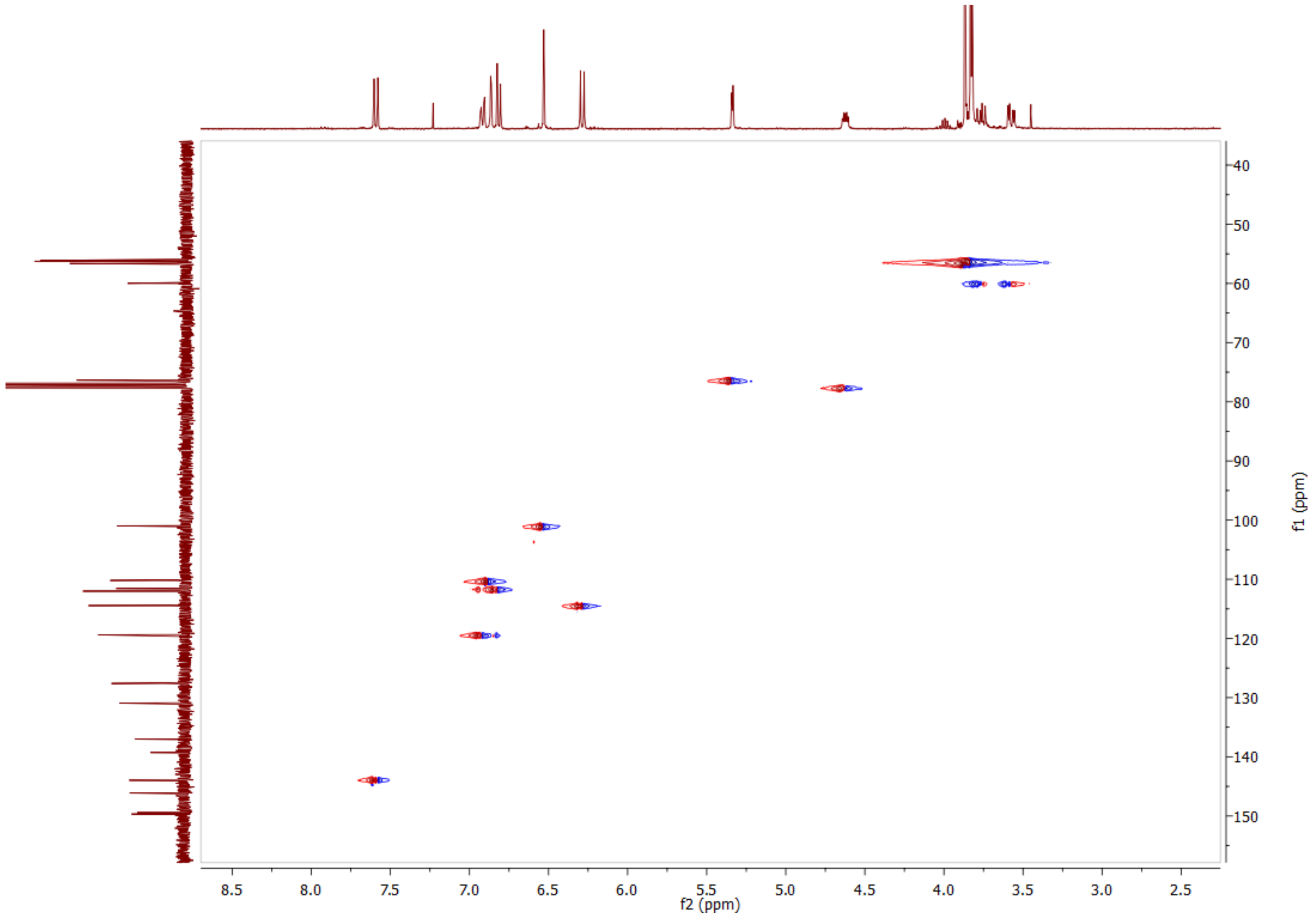

Figure S57. HSQC spectrum of the new compound **7**

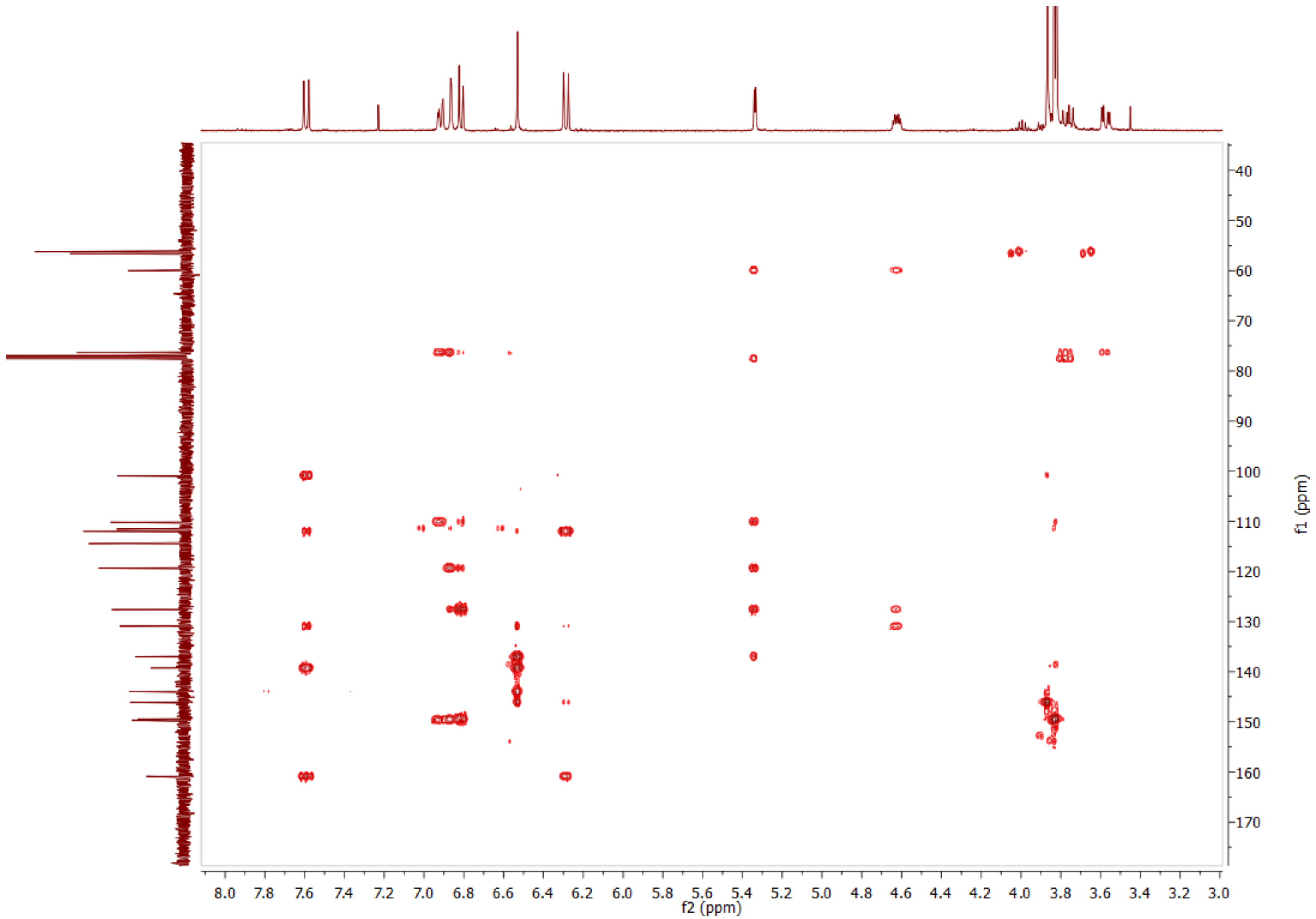

Figure S58. HMBC spectrum of the new compound **7**

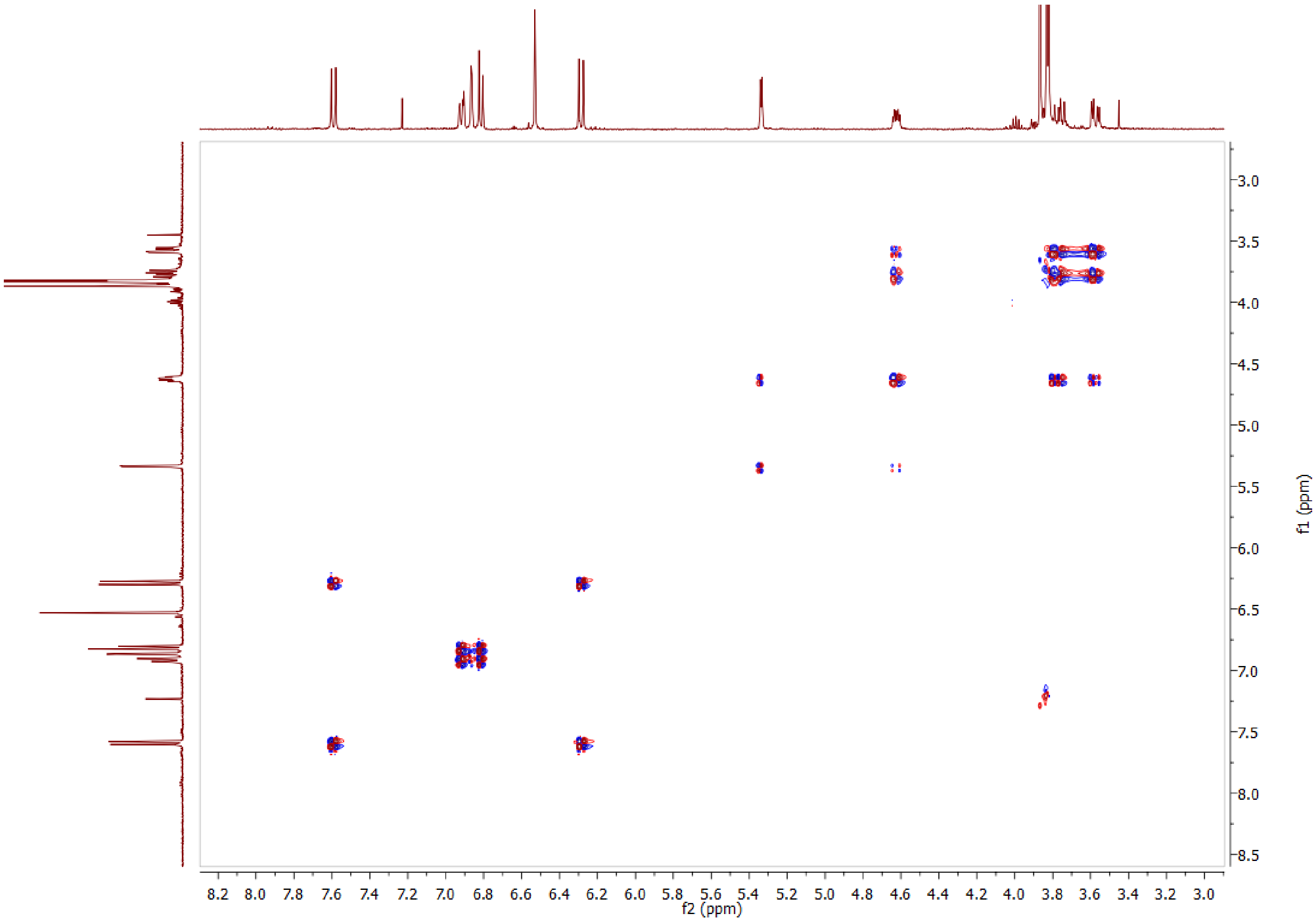

Figure S59. COSY spectrum of the new compound **7**

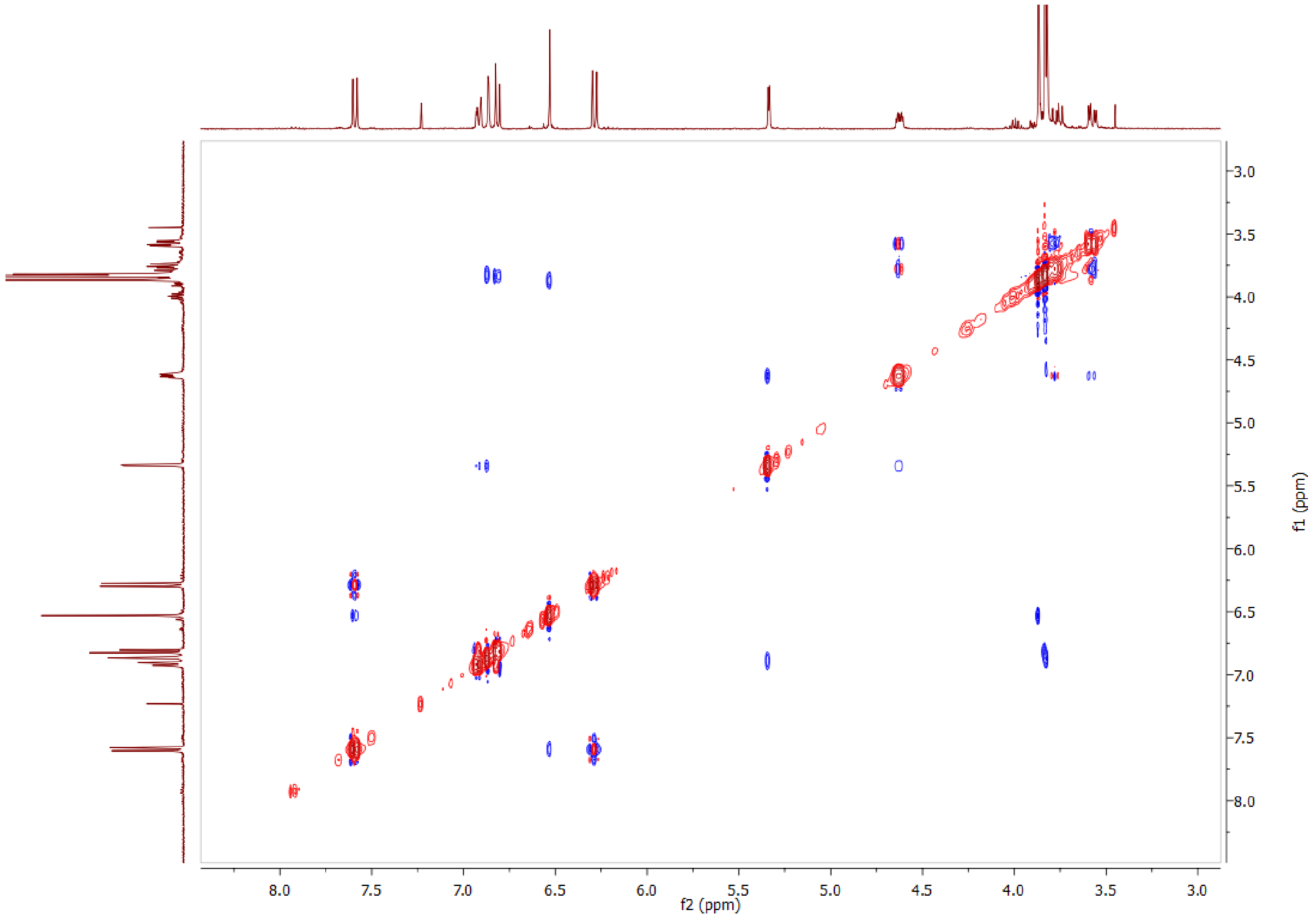

Figure S60. NOESY spectrum of the new compound **7**

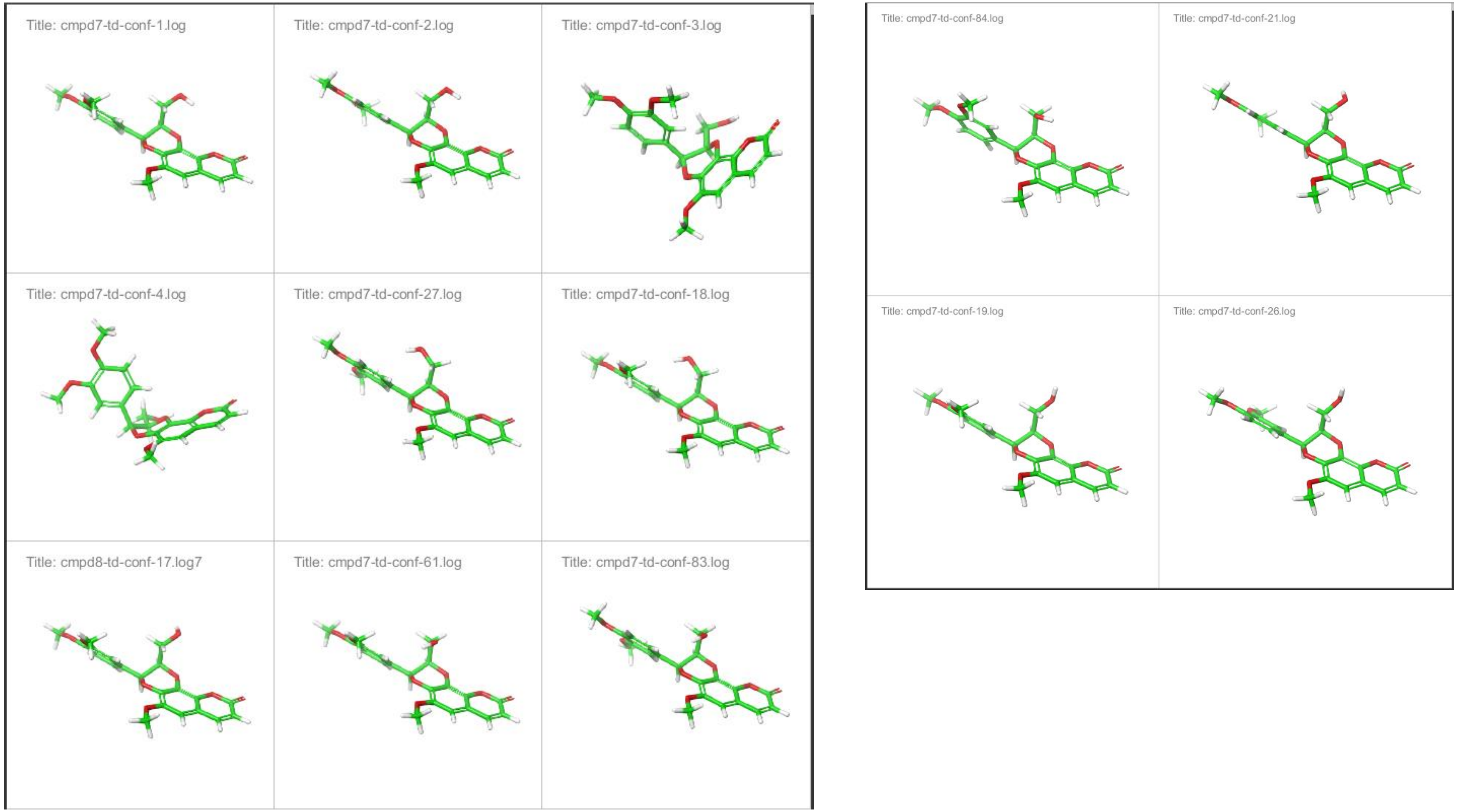

Figure S61. Images of conformers (>1%) for the new compound **7**
